# Supplementary material for: Dimerization of ADAR1 modulates site-specificity of RNA editing
Source: Nat Commun. 2024 Nov 21;15:10051. doi: 10.1038/s41467-024-53777-2 (PMC11582362; doi:10.1038/s41467-024-53777-2)
Supplement: Supplementary file 1 — Supplementary Information [file 41467_2024_53777_MOESM1_ESM.pdf]

Supplementary information

Dimerization of ADAR1 modulates site-specificity of RNA editing

Allegra Mboukou, Vinod Rajendra, Serafina Messmer, Therese C. Mandl, Marjorie Catala, Carine Tisné, Michael F. Jantsch & Pierre Barraud

Content:

Supplementary Tables S1 to S5 pages 2-6
Supplementary Figures S1 to S13 pages 7-18

Supplementary Table S1: SAXS data collection and analysis.

**(*a*) Sample details**						
	dsRBD3-long	dsRBD3-mid	dsRBD3-short	ADAR1-dsRBD3	Interface mutant	Chimeric ADAR1/Xlrbpa
Organism			*Human*			*Human/Xenopus*
Uniprot ID (range)	P55265 (688-817)	P55265 (708-801)	P55265 (716-797)	P55265 (708-801)	P55265 (708-801)	P55265/Q91836 (708-801)
Molecular weight (kDa)	14.7	10.8	9.2	12.6	12.5	12.8
Mode of measurement			Online SEC-SAXS			
SEC-column			BioSEC-3 column (Agilent)			
Flow rate (ml/min)			0.3			
Injection volume (μL)			50			
Temperature (°C)			15			
Protein [conc.] (mg/mL)			5			
Buffer	20 mM Na-HEPES pH 7.3, 55 mM KOAc, 10 mM NaCl, 1 mM TCEP			20 mM Na-phosphate pH 7.0, 100 mM NaCl, 2 mM 2-mercaptoethanol		

**(*b*) SAS data collection parameters**	
SAXS beamline	SWING, SOLEIL, Saint-Aubin, France
Wavelength (nm)	0.1033
Detector	EigerX4M (162.5 x 155.2 mm^2)
Detector distance (m)	2.00
s-measurement range ($Å^{-1}$)	0.005-0.55
Exposure time	0.99 s

**(*c*) Software employed for SAS data reduction, analysis and interpretation**	
SAS data reduction	FOXTROT v3.5.4
Basic analyses: Guinier, *P*(*r*), scattering particle volume, molecular weight	ATSAS v3.0.3, PRIMUS, GNOM
Shape/bead modelling	DAMMIN, GASBOR
Model validation	CRYSOL
Modelling of missing side chains in PDB file	COOT
Molecular graphics	PYMOL

**(*d*) Structural parameters**						
Guinier Analysis	dsRBD3-long	dsRBD3-mid	dsRBD3-short	ADAR1-dsRBD3	Interface mutant	Chimeric ADAR1/Xlrbpa
I(0) (arbitrary units)	0.02 ± 0.000038	0.013 ± 0.00003	0.017 ± 0.000029	0.015 ± 0.000041	0.01 ± 0.000023	0.011 ± 0.000019
R_g (Å)	25.5	20.1	19.2	24.4	21.3	20.2
s-range ($Å^{-1}$)	0.0215-0.0508	0.0160-0.0648	0.0114-0.0670	0.0306-0.0529	0.0297-0.0611	0.0219-0.0611
min < sRg < max limit	0.55-1.29	0.32-1.30	0.22-1.29	0.75-1.29	0.63-1.30	0.45-1.25
P(*r*) analysis	dsRBD3-long	dsRBD3-mid	dsRBD3-short	ADAR1-dsRBD3	Interface mutant	Chimeric ADAR1/Xlrbpa
I(0) (arbitrary units)	0.0195 ± 0.000034	0.0126 ± 0.000032	0.0166 ± 0.000027	0.0153 ± 0.000037	0.0102 ± 0.000022	0.0112 ± 0.000017
R_g (Å)	25.7	20.1	18.9	25.3	22.1	20.8
d_{max} (Å)	80	67	55	91	72	64
s-range ($Å^{-1}$)	0.0215-0.313	0.0160-0.3299	0.0114-0.4162	0.0306-0.3271	0.0297-0.330	0.0219-0.3299
Porod volume (nm^3)	49.6	26.7	26.8	40.0	19.6	18.7

**(*e*) Model validation**						
	dsRBD3-long	dsRBD3-mid	dsRBD3-short	ADAR1-dsRBD3	Interface mutant	Chimeric ADAR1/Xlrbpa
Method			CRYSOL			
s-range for fitting			0.0052-0.4810			
χ^2 value			1.30			
PDB coordinates			7ZJ1			

**(*f*) Molecular weight (kDa)**						
	dsRBD3-long	dsRBD3-mid	dsRBD3-short	ADAR1-dsRBD3	Interface mutant	Chimeric ADAR1/Xlrbpa
Molecular weight from sequence	14.7	10.8	9.2	12.6	12.5	12.8
Estimated molecular weight	30.9	18.7	21.2	24.3	12.0	11.2
Confidence interval (95%)	29.2-33.4	17.8-19.6	19.6-22.7	22.7-25.3	10.8-13.1	10.8-12.4
Oligomeric state	dimer	dimer	dimer	dimer	monomer	monomer

**(*g*) Data and model deposition IDs**						
	dsRBD3-long	dsRBD3-mid	dsRBD3-short	ADAR1-dsRBD3	Interface mutant	Chimeric ADAR1/Xlrbpa
SASBDB codes	SASDVF7	SASDVG7	SASDVH7	SASDVJ7	SASDVK7	SASDVL7

Supplementary Table S2: Crystallographic data collection and refinement statistics.

**Structure**	**ADAR1-dsRBD3 dimer**	**ADAR1-dsRBD3:dsRNA**
**PDB code**	7ZJ1	7ZLQ
**Data collection**		
**Beamline**	SOLEIL Proxima-2 (PX2)	SOLEIL Proxima-2 (PX2)
**Wavelength**	0.9801	0.9999
**Space group**	P 31 2 1	P 61 2 2
**Unit cell**	44.2353 44.2353 131.803 90 90 120	91.294 91.294 207.527 90 90 120
**Resolution range**	19.06–1.65 (1.71–1.65)	44.58–2.80 (2.99–2.80)
**Total reflections**	351229 (26321)	192634 (9181)
**Unique reflections**	18792 (1852)	10044 (503)
**Multiplicity**	18.7 (14.2)	19.2 (18.3)
**Completeness (%)**	99.26 (99.84)	75.9 (22.1) [94.9 (88.6)][a]
**Mean I/sigma(I)**	12.45 (1.53)	16.6 (1.2)
**R-merge**	0.1518 (0.8674)	0.163 (2.663)
**R-pim**	0.0354 (0.2381)	0.038 (0.633)
**$CC_{1/2}$**	0.995 (0.585)	1.000 (0.517)
**Refinement**		
**Reflections used in refinement**	18670 (1854)	10026 (503)
**Reflections used for R-free**	943 (92)	1078 (73)
**R-work**	0.1871 (0.2079)	0.2311 (0.3278)
**R-free**	0.2247 (0.2540)	0.2658 (0.3526)
**Model composition**		
**Number of non-hydrogen atoms**	1364	2079
**macromolecules**	1248	2079
**solvent**	116	0
**Protein residues**	166	164
**RNA residues**	0	39
**Model validation**		
**Clashscore**	3.25	7.99
**Rotamer outliers (%)**	0.00	0.00
**Average B-factor (Å^2)**	20.45	80.97
**protein**	19.87	79.95
**RNA**	–	82.54
**solvent**	26.67	–
**Number of TLS groups**	2	5
**Ramachandran statistics (%)**		
**Favored**	98.15	97.50
**Allowed**	1.85	2.50
**Outliers**	0.00	0.00
**RMS deviations**		
**Bond length (Å)**	0.010	0.007
**Bond angles (°)**	1.08	0.88

Statistics for the highest-resolution shell are shown in parentheses.

[a]Values in brackets correspond to the ellipsoidal completeness as reported by STARANISO

Supplementary Table S3: Oligonucleotides.

MJ8371	GATACCTGAACACCAACCCTGTGGGTGGCCTTTT GGAGTAC	Insert forward primer used for creating plasmids MJ1645,1648
MJ8372	CCCCAATCAAGACACGGAGAGCCGCATCTGCTGC TTCCTG	Insert reverse primer used for creating plasmids MJ1645,1648
MJ8373	GATGCGGCTCTCCGTGTCTTGATTGGGGAGAACG AGAAGG	Vector forward primer used for creating plasmids MJ1645,1648
MJ8374	CCACCCACAGGGTTGGTGTTCAGGTATCTCACGA GCTCGCC	Vector reverse primer used for creating plasmids MJ1645,1648
MJ8610	GGCCACCATGTACCCATACGATGTTCCAGATTAC GCTATGGCCGAGATCAAGG	Insert forward primer used for creating plasmids MJ1723, MJ1724
MJ8611	CCCTCTCCACTGCCGACTAGTACTGGGCAGAGAT AAAAGTTCTTTTCCTCCTG	Insert reverse primer used for creating plasmids MJ1723, MJ1724
MJ8612	CTGCCCAGTACTAGTCGGCAGTGGAGAGGGCAGA GGAAGTCTGCTAACATG	Vector forward primer used for creating plasmids MJ1723, MJ1724
MJ8613	GCGTAATCTGGAACATCGTATGGGTACATGGTGG CCAGATATCCAGCACAG	Vector reverse primer used for creating plasmids MJ1723, MJ1724
MJ8697	GCAGTGGAGAGGGCAGAGGAAGTCTGCTAACATG CGGTG	Vector forward primer used for creating plasmids MJ1731, MJ1732
MJ8698	GCAGACTTCCTCTGCCCTCTCCACTGCCGAGTGT CTTTGGCTG	Insert reverse primer used for creating plasmids MJ1731, MJ1732
MJ8749	GAAGGATCTGGTGTTAAGATAATTTCAGAACCCG	Forward primer Azin1
MJ8750	ACTGGAATGTTGACCAGACAAGCTTAACC	Reverse primer Azin1
MJ8755	CGAGCCGAGTATCCAGGATACAAC	Forward primer Gli1
MJ8756	CCCATATCCCAGAGTATCAGTAGGTGG	Reverse primer Gli1
MJ984	CCCACTCCTGGATCTTCAC	Forward primer Cflar
MJ985	CAGGTTGGTATGCAGTGGC	Reverse primer Cflar
MJ8936	CTGAGCATAAGAACTGGGACCCTTC	Forward primer for Nicn1
MJ8937	CTCATTATGCTGAGCATATCTCTTGG	Reverse primer for Nicn1

* MJ= oligo numbers according Jantsch lab oligo database

Supplementary Table S4: Mammalian expression plasmids.

MJ1209	FLAG-human ADAR1 p110-6X His in pCDNA 3.1, Gift from Mary O' Connell lab
MJ1210	FLAG-human ADAR1 p150-6X His in pCDNA 3.1, Gift from Mary O' Connell lab
MJ1645	FLAG-human ADAR1 p110 dsRBD3 mutant-6X His in pCDNA 3.1
MJ1648	FLAG-human ADAR1 p150 dsRBD3 mutant-6X His in pCDNA 3.1
MJ1723	HA-human ADAR1 p110 T2A eGFP in pCDNA 3.1
MJ1724	HA-human ADAR1 p110 dsRBD3 mutant T2A eGFP in pCDNA 3.1
MJ1731	HA-human ADAR1 p110 Δ deaminase T2A eGFP in pCDNA 3.1
MJ1732	HA-human ADAR1 p110 dsRBD3 mutant Δ deaminase T2A eGFP in pCDNA 3.1

* MJ= plasmid numbers according Jantsch lab plasmid database, MJ1731 and MJ1732 expresses amino acids from 296 to 833 (Methionine 296 is the start for p110 variant of ADAR1)

Supplementary Table S5: Genomic coordinates of editing sites.

Azin1 Site 1	chr8:102829408
Azin1 Site 2	chr8:102829879
Gli1	chr12:57470841
Cflar Site 1	chr2:201164032
Cflar Site 2	chr2:201164087
Cflar Site 3	chr2:201164088
Cflar Site 4	chr2:201164105
Cflar Site 5	chr2:201164112
Cflar Site 6	chr2:201164118
Nicn Site 2069	chr3:49423491
Nicn Site 2066	chr3:49423494
Nicn Site 2062	chr3:49423498
Nicn Site 2060	chr3:49423500
Nicn Site 2048	chr3:49423512
Nicn Site 2047	chr3:49423513
Nicn Site 2046	chr3:49423514
Nicn Site 2025	chr3:49423535
Nicn Site 2020	chr3:49423540
Nicn Site 2015	chr3:49423545
Nicn Site 2008	chr3:49423552
Nicn Site 2007	chr3:49423553
Nicn Site 2006	chr3:49423554
Nicn Site 1995	chr3:49423565
Nicn Site 1969	chr3:49423591
Nicn Site 1966	chr3:49423594
Nicn Site 1960	chr3:49423600
Nicn Site 1953	chr3:49423607
Nicn Site 1952	chr3:49423608
Nicn Site 1926	chr3:49423634
Nicn Site 1916	chr3:49423644
Nicn Site 1904	chr3:49423656
Nicn Site 1887	chr3:49423673
Nicn Site 1886	chr3:49423674
Nicn Site 1880	chr3:49423680
Nicn Site 1867	chr3:49423693

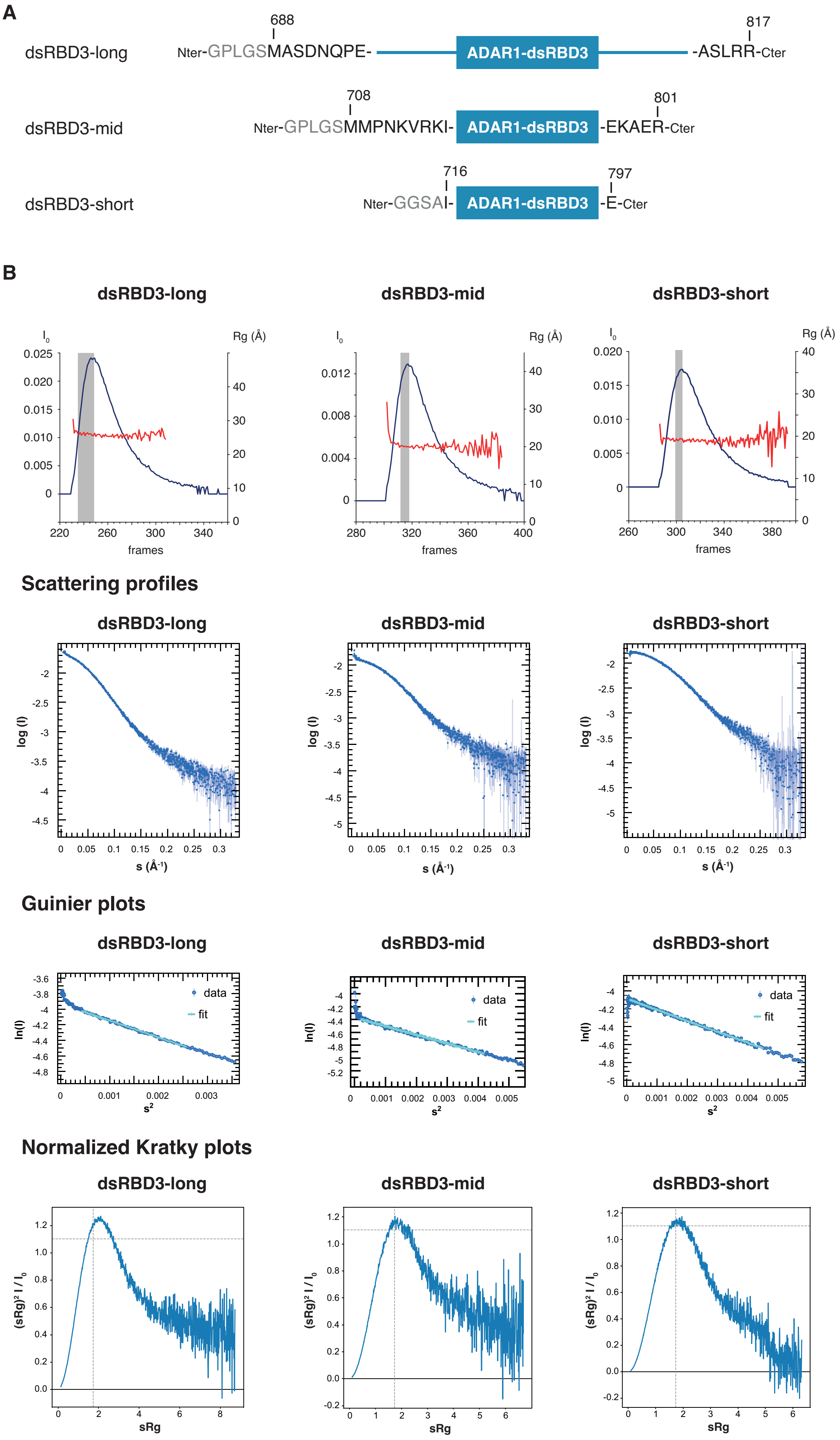

Supplementary Figure S1

Supplementary Figure S1: Characterization of various ADAR1-dsRBD3 constructs with different length of N- and C-terminal flanking tails by SAXS.

(**A**) Schematic representation of ADAR1-dsRBD3 constructs: dsRBD3-long (residues 688-817), dsRBD3-mid (residues 708-801) and dsRBD3-short (residues 716-797). Constructs used here had their purification tags cleaved off. Remaining residues after tag-cleavage are shown in grey. (**B**) SAXS data are presented for the ADAR1-dsRBD3 constructs displayed on panel A. I_0 and Rg values obtained around the elution peak of HPLC are displayed in the upper panel. Selected frames are in shown with a gray background and the corresponding scattering profiles are shown in upper panel. Regions used for the Guinier approximation are shown in the middle panel (Guinier plots). Normalized Kratky plots are shown in the lower panel. Derived parameters are reported on Table 1. See also Supplementary Table S1. Scattering data have been deposited to the SASBDB (accession codes SASDVF7, SASDVG7, and SASDVH7).

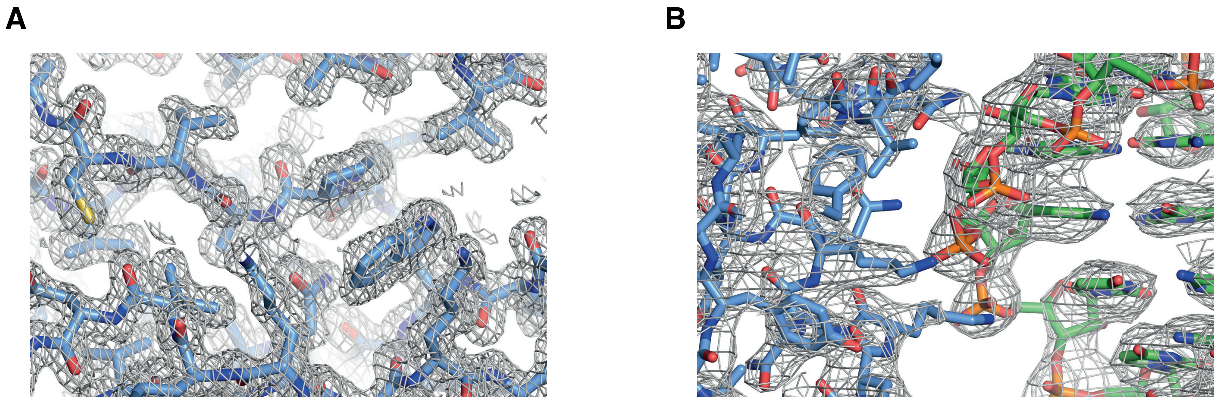

Supplementary Figure S2: Electron density maps ($2F_o{-}F_c$ at 1σ) of ADAR1-dsRBD3 dimer free and bound to dsRNA.

(**A**) Close-up view of the structure and electron density map ($2F_o{-}F_c$ at 1σ) of the free ADAR1-dsRBD3 dimer (PDB code 7ZJ1). (**B**) Close-up view of the structure and electron density map ($2F_o{-}F_c$ at 1σ) of the ADAR1-dsRBD3 dimer bound to dsRNA (PDB code 7ZLQ). The protein residues and the RNA nucleotides are shown as blue and green sticks, respectively. The electron density map is displayed as a grey mesh. See also Supplementary Table S2. Data have been deposited to the PDB (accession codes 7ZJ1 and 7ZLQ).

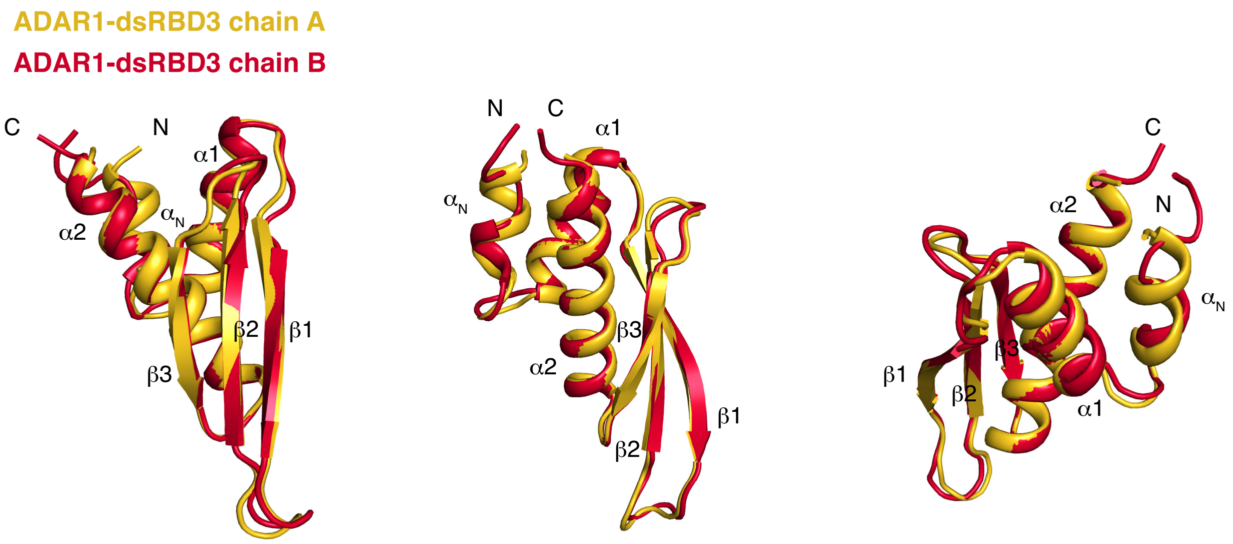

Supplementary Figure S3: Comparison of ADAR1-dsRBD3 chain A and chain B in the asymmetric unit.
ADAR1-dsRBD3 chain A (*in yellow*) and chain B (*in red*) are shown as cartoon. Both chains were superimposed over Cα atoms over the entire domain. Secondary structure elements are labelled. Most differences occur at the level of the N-terminal helix α_N, where the distortion on chain B is likely caused by packing interactions with symmetrical molecules. Small structural differences also occur at the level of loop 1 (α1-β1), loop 2 (β1-β2), and loop 3 (β2-β3). Data have been deposited to the PDB (accession code 7ZJ1).

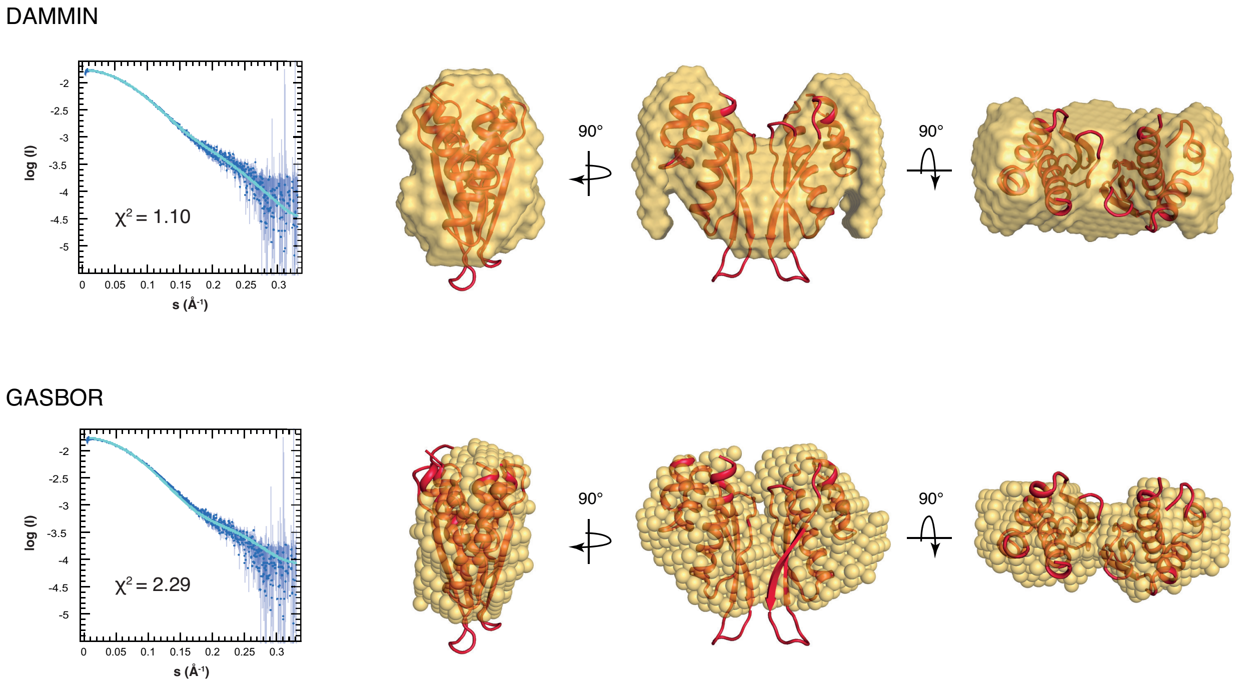

Supplementary Figure S4: *Ab initio* shape reconstruction of ADAR1-dsRBD3.
Shape reconstructions of ADAR1-dsRBD3 (dsRBD-short construct) with averaged DAMMIN (top) and GASBOR (bottom) models. The DAMMIN shape is shown as a semi-transparent surface (*in light yellow*), while the dummy residue beads are shown as semi-transparent spheres for the GASBOR shape (*in light yellow*). The crystal structure of ADAR1-dsRBD3 dimer was aligned to the *ab initio* models using CIFSUP and in depicted as a cartoon (*in red*) fitting into the *ab initio* models. Fits for the most representative model of the most populated cluster determined by DAMAVER are shown over the raw scattering data. The corresponding goodness of fit (χ^2) are reported in each case. Scattering data have been deposited to the SASBDB (accession codes SASDVH7).

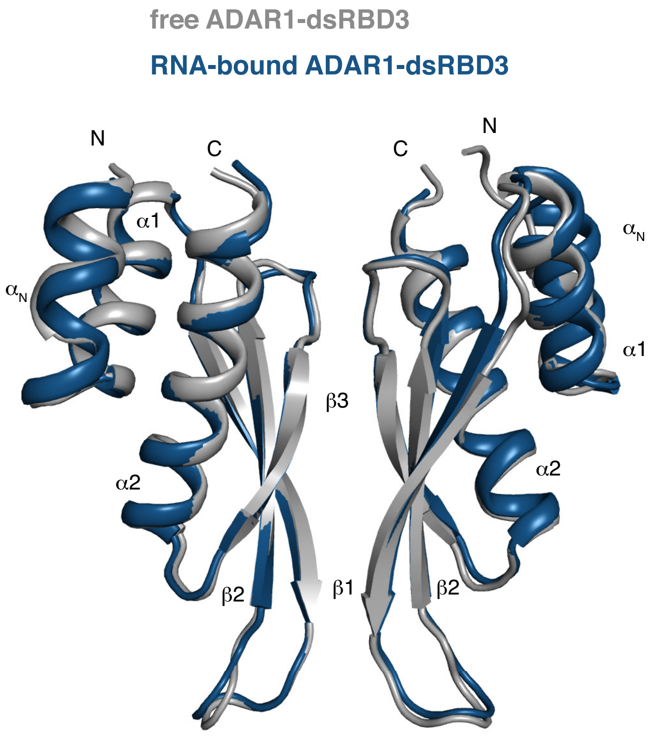

Supplementary Figure S5: Comparison of free ADAR1-dsRBD3 dimer and RNA-bound ADAR1-dsRBD3 dimer.

Free ADAR1-dsRBD3 (*in grey* – PDB code 7ZJ1) and RNA-bound ADAR1-dsRBD3 (*in blue* – PDB code 7ZLQ) are shown as cartoon. Both structures were superimposed over Cα atoms over the entire domains. Secondary structure elements are labelled. The two structures are perfectly superimposable, meaning that the dimer organization is identical in both structures. Data have been deposited to the PDB (accession codes 7ZJ1 and 7ZLQ).

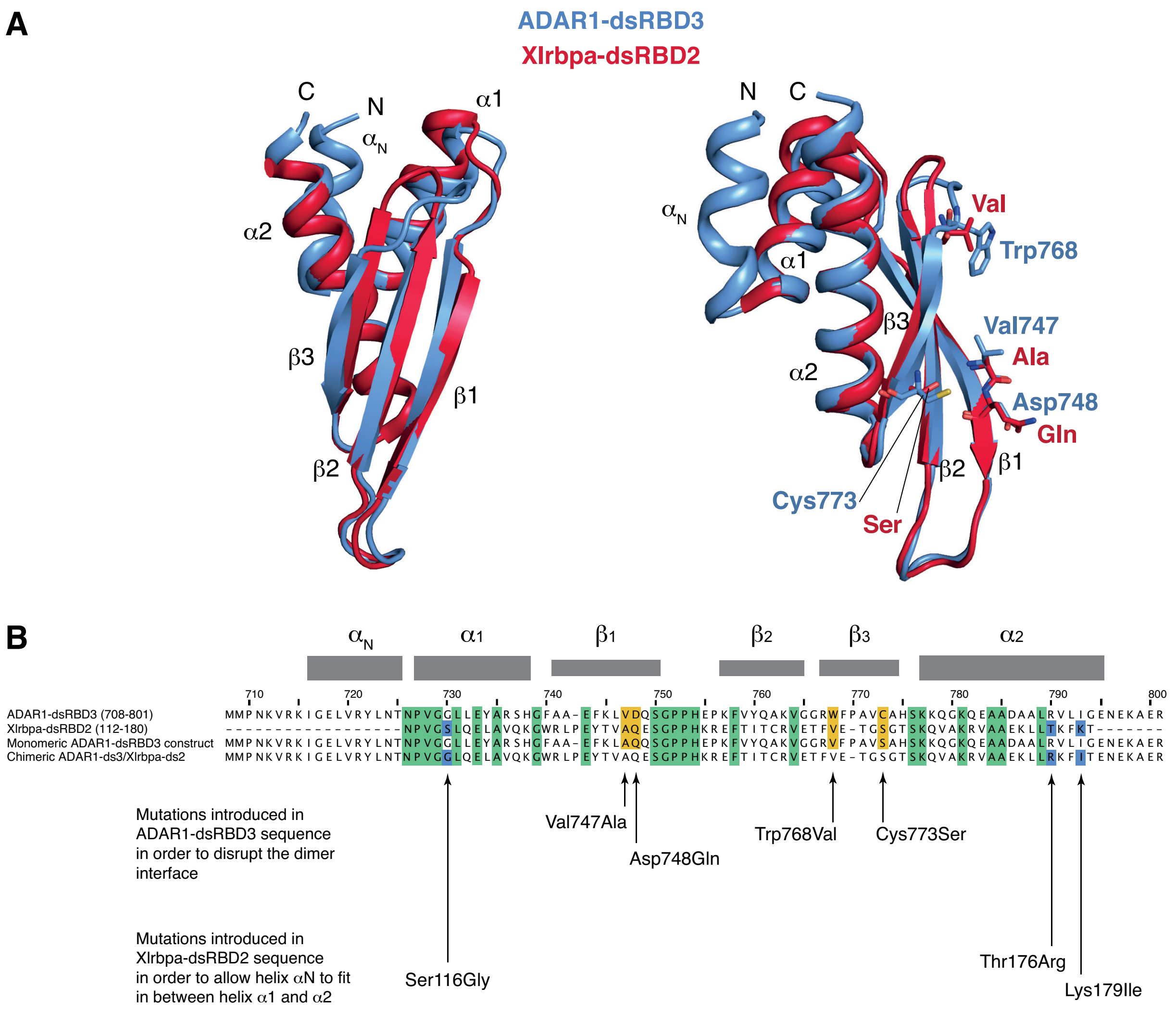

Supplementary Figure S6: Rational design of ADAR1-dsRBD3 mutants to disrupt the dimer interface. (**A**) ADAR1-dsRBD3 (*in light blue*) and Xlrbpa-dsRBD2 (*in red*) are shown as cartoon. Both chains were superimposed over Cα atoms over the entire domains. Secondary structure elements are labelled. Residues at the β-sheet interface involved in the monomer-monomer interaction (see Figure 1) and that differ between ADAR1-dsRBD3 and Xlrbpa-dsRBD2 (see the sequence alignment on panel B), are shown as sticks. (**B**) Sequence alignment of ADAR1-dsRBD3 (708-801), Xlrbpa-dsRBD2 (112-180), the monomeric ADAR1-dsRBD3 construct that include four point-mutations at the dimer interface in which ADAR1-dsRBD3 residues are mutated into those found in Xlrbpa-dsRBD2 (i.e. V747A and D748Q in strand β1 and W768V and C773S in strand β3), and the chimeric construct resulting from the combination of ADAR1-dsRBD3 N- and C-terminal fragments flanking the Xlrbpa-dsRBD2 domain. Residue numbering and secondary structure elements of ADAR1-dsRBD3 are shown above the alignment. Identical residues between ADAR1-dsRBD3 and Xlrbpa-dsRBD2 are highlighted *in green*. The four mutations introduced in ADAR1-dsRBD3 in order to disrupt the dimer interface (i.e. V747A, D748Q, W768V and C773S) are shown *in yellow*. The three mutations introduced in Xlrbpa-dsRBD2 in order to adapt the surface of Xlrbpa-dsRBD2 for enabling the tight interaction of helix αN in between helices α1 and α2 (i.e. S116G, T176R and K179I) are shown *in blue*.

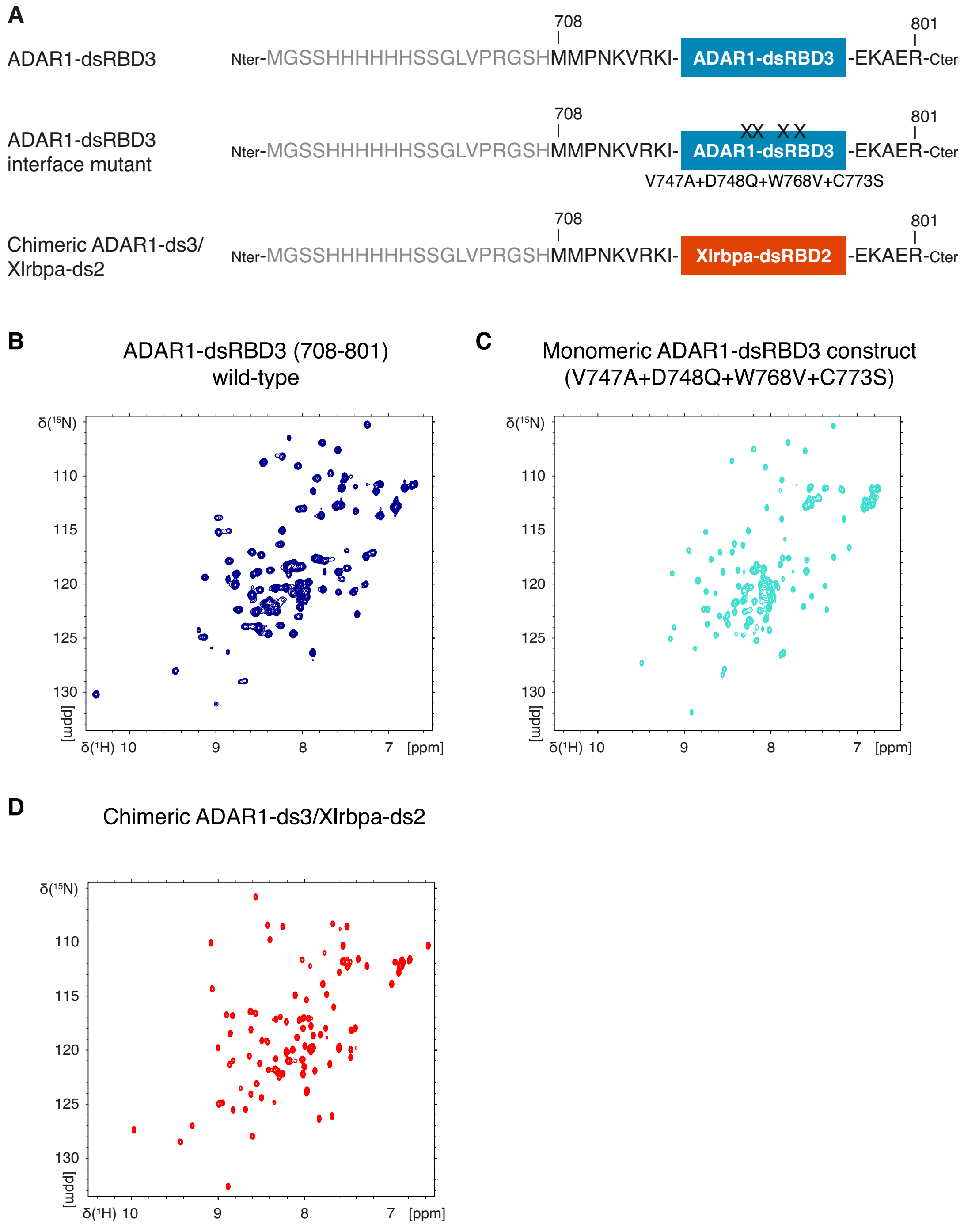

Supplementary Figure S7: Monomeric ADAR1-dsRBD3 mutant and chimeric ADAR1-dsRBD3/Xlrbpa-dsRBD2 are well-folded domains.
(**A**) Schematic representation of ADAR1-dsRBD3 constructs: ADAR1-dsRBD3 (residues 708-801), ADAR1-dsRBD3 interface mutant (residues 708-801; V747A+D748Q+W768V+C773S), and chimeric ADAR1-ds3/Xlrbpa-ds2 (see Supplementary Figure S4). Constructs used here retained their purification tags. Residues of the N-terminal His_6-tag are shown in grey. (**B**) (^1H,^{15}N)-HSQC spectra from ADAR1-dsRBD3 wild-type (*in deep blue*). (**C**) (^1H,^{15}N)-HSQC spectra from monomeric ADAR1-dsRBD3 (mutant V747A+D748Q+W768V+C773S) (*in light blue*). (**D**) (^1H,^{15}N)-HSQC spectra from chimeric ADAR1-dsRBD3/Xlrbpa-dsRBD2 (*in red*). The dispersion of amide signals in all constructs (panel B-D) is the sign of well-folded domains. Source data are provided as Source Data files.

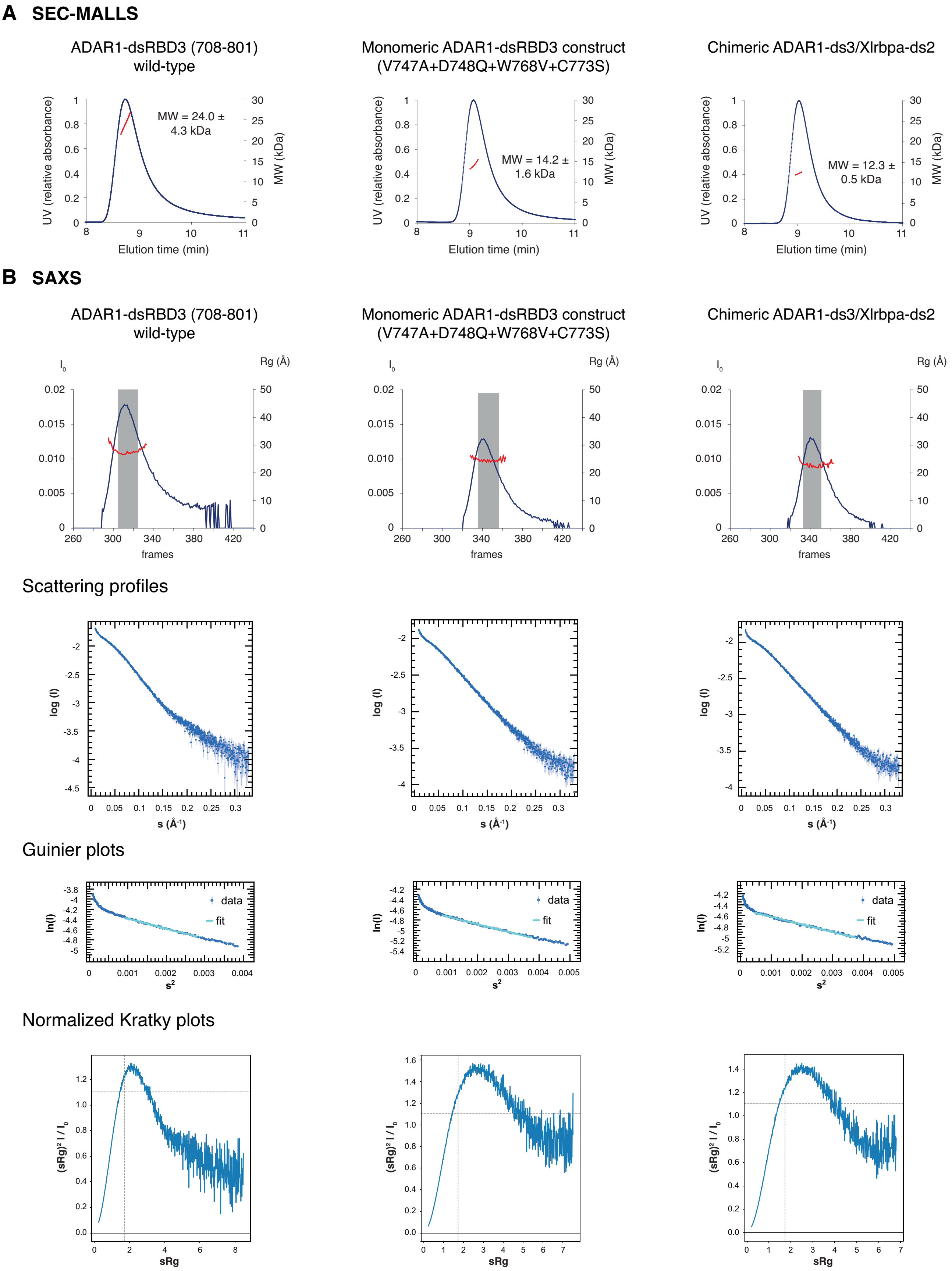

Supplementary Figure S8

Supplementary Figure S8: Characterization of monomeric ADAR1-dsRBD3 and chimeric ADAR1-dsRBD3/Xlrbpa-dsRBD2 constructs by SEC-MALLS and SAXS.
SEC-MALLS and SAXS data are presented for the following ADAR1-dsRBD3 constructs: ADAR1-dsRBD3 wild-type (residues 708-801), ADAR1-dsRBD3 interface mutant (residues 708-801; V747A+D748Q+W768V+C773S), and chimeric ADAR1-ds3/Xlrbpa-ds2. Constructs used here retained their purification tags (see Supplementary Figures S4 and S5). (**A**) SEC-MALLS analysis of the constructs on a BioSEC-3 column. The UV relative absorbance and the estimated molecular weight (MW) obtained around the elution peak of HPLC are displayed in blue and in red, respectively. Molecular weights values are directly reported on the figure for each construct. Source data are provided as Source Data files. (**B**) I_0 and Rg values obtained around the elution peak of HPLC are displayed in the upper panel. Selected frames are in shown with a gray background and the corresponding scattering profiles are shown in upper panel. Regions used for the Guinier approximation are shown in the middle panel (Guinier plots). Normalized Kratky plots are shown in the lower panel. Derived parameters are reported on Table 2. See also Supplementary Table S1. Scattering data have been deposited to the SASBDB (accession codes SASDVJ7, SASDVK7, and SASDVL7).

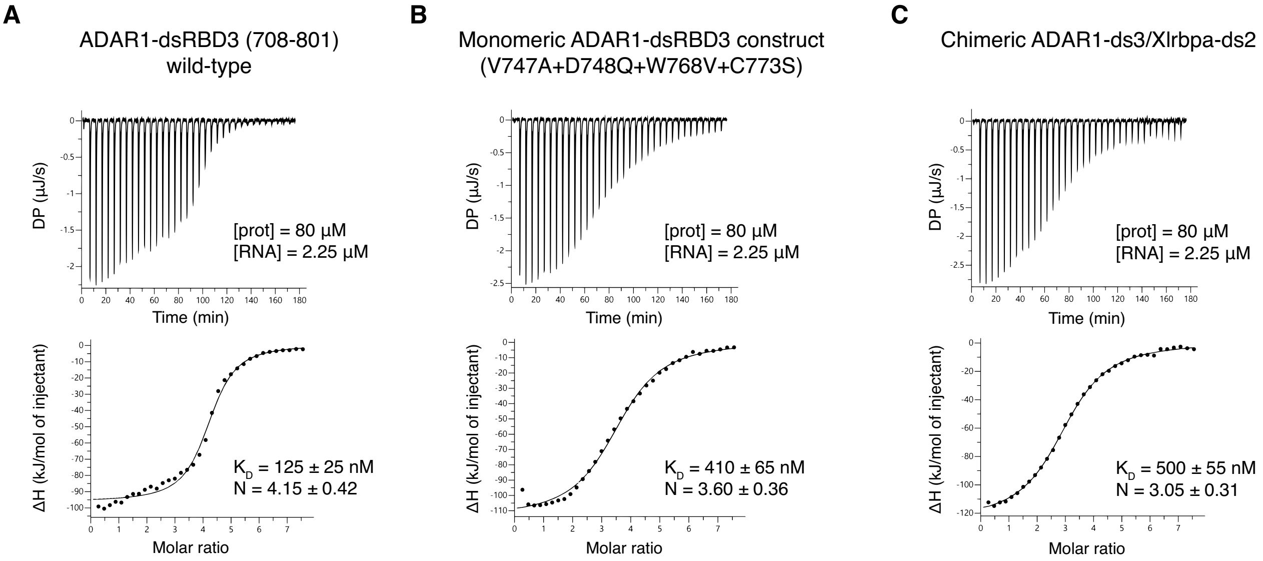

Supplementary Figure S9: Monomeric ADAR1-dsRBD3 remains competent for RNA-binding.
Isothermal titration calorimetry monitoring the interaction between ADAR1-dsRBD3 wild-type (**A**), monomeric ADAR1-dsRBD3 construct (**B**), and chimeric ADAR1-dsRBD3/Xlrbpa-dsRBD2 construct (**C**) and a 24 bp dsRNA duplex. K_D: dissociation constant in nM. N: number of sites. Values are reported as means ± standard error (S.E.). The uncertainties on the fitted parameters were estimated from the data spread and from the uncertainty of the protein concentration determination (10%). Source data are provided as Source Data files.

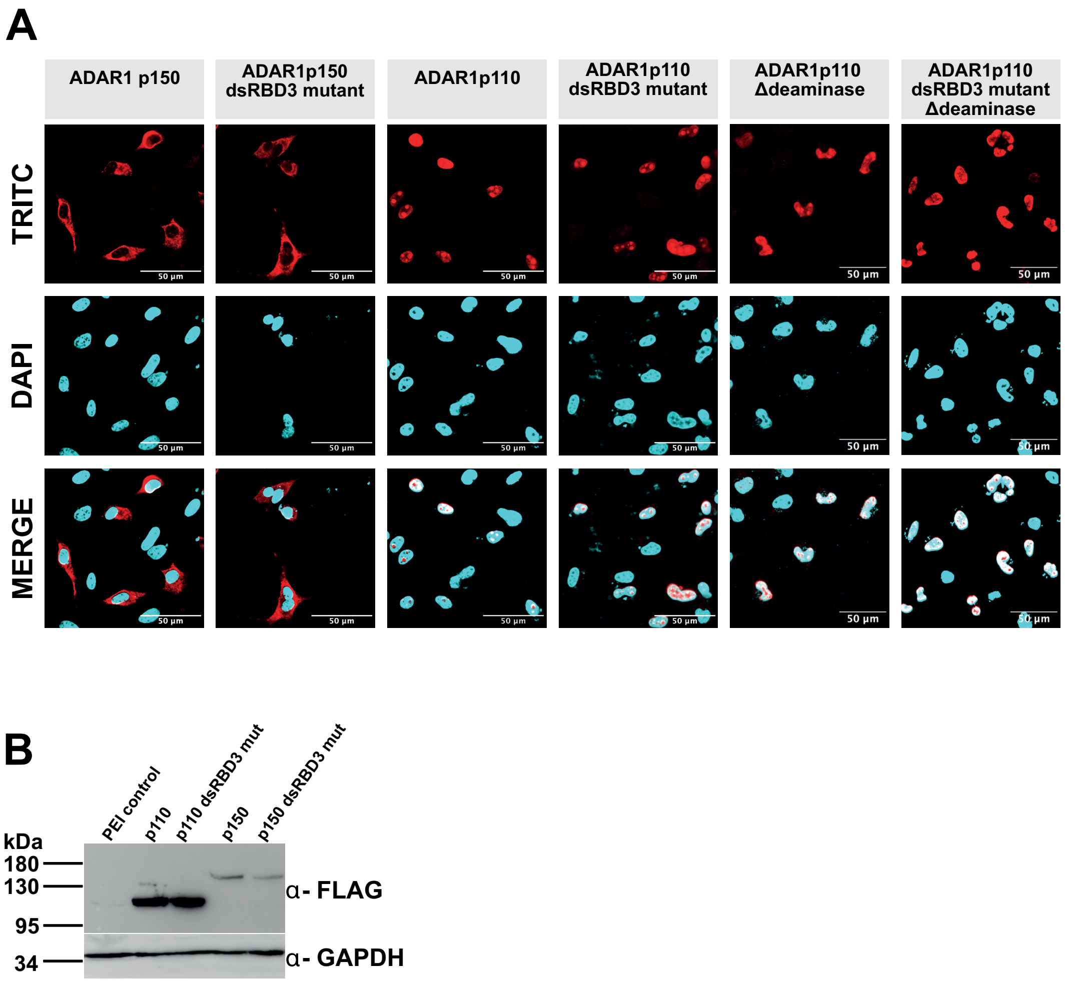

Supplementary Figure S10: ADAR1-dsRBD3 interface mutations and deletion of the deaminase domain does not affect cellular localization.

(**A**) Confocal microscopy images confirm the cellular localization of ADAR1 variants. Confocal sections of transfected constructs are visualized under TRITC channel. DAPI is used for nuclear staining. ADAR1 p150 localizes to the cytoplasm (scale bar: 50 µm). (**B**) Western blots confirm the similar level of expression of ADAR1 p110 with respect to its dsRBD3 dimerization mutant (p110 dsRBD3 mut) and ADAR1 p150 (p150) with respect to its dsRBD3 dimerization mutant (p150 dsRBD3 mut). Expression of proteins detected by anti-FLAG antibodies. GAPDH serves as a loading control and PEI control indicate transfected cells without expression plasmids. Source data are provided as Source Data files.

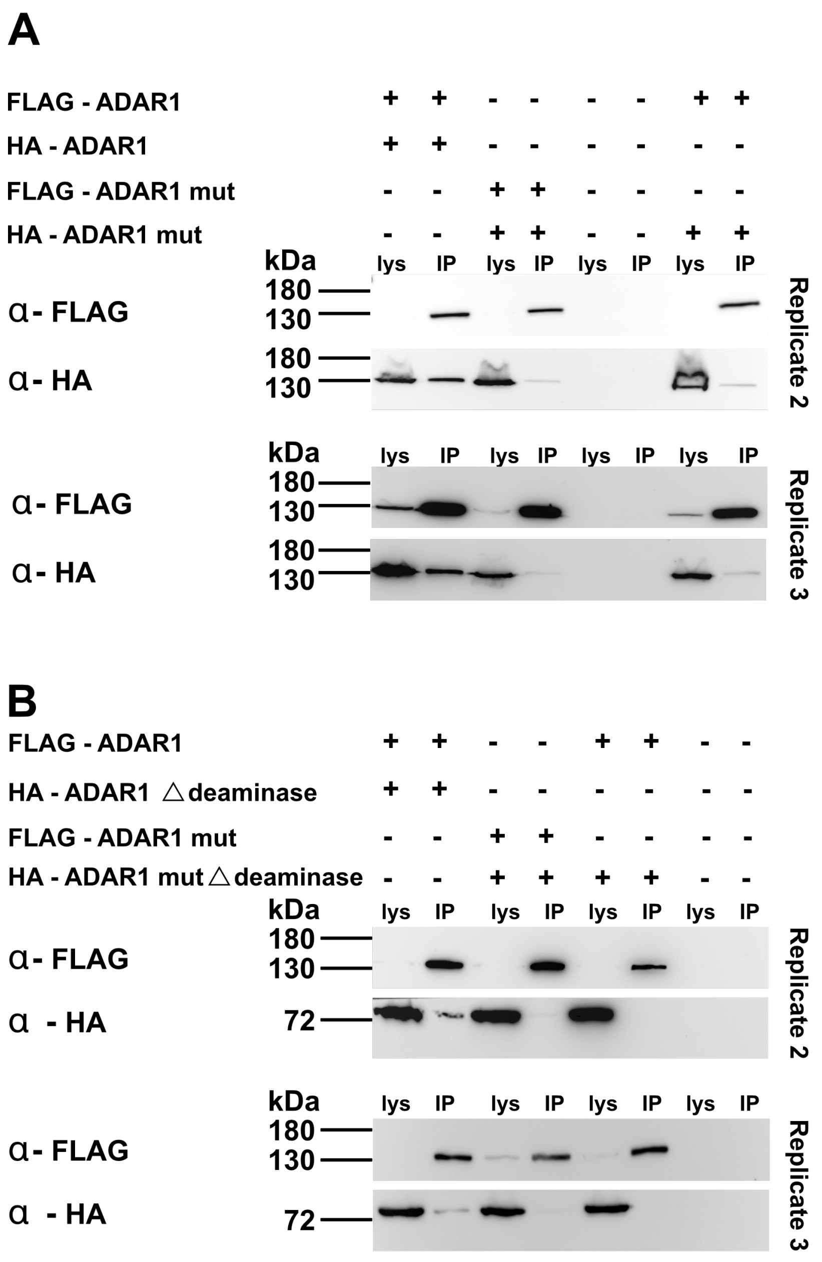

Supplementary Figure S11: ADAR1-dsRBD3 interface mutations disrupt dimer formation in vivo.
(**A**) Western blot images used for quantification of immunoprecipitation of full-length proteins (for main Figure 4B) (**B**) Western blot images used for quantification of immunoprecipitation of deaminase deletion variants (for main Figure 4D). lys: lysate; IP: immunoprecipitation. Blots are probed with anti-FLAG (α-FLAG) and anti-HA (α-HA) antibodies. Replicates 2 and 3 are shown. Replicate 1 is shown in Figure 4. Source data are provided as Source Data files.

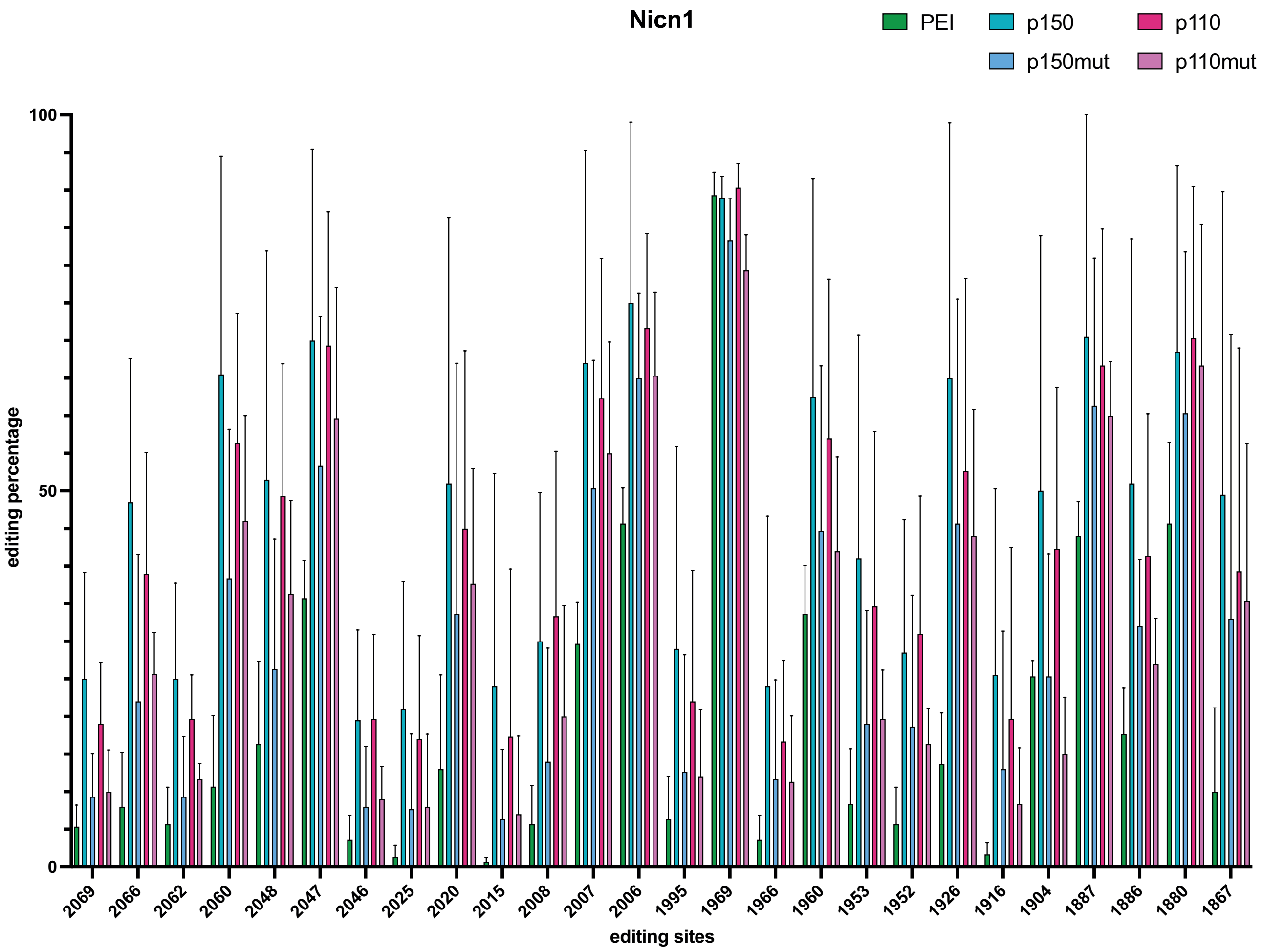

Supplementary Figure S12: Editing levels at 26 sites in the 3' UTR of Nicn1.
PEI shows endogenous editing levels of cells only exposed to the transfection reagent. p110 and p110mut indicates editing levels in cells transfected with wild-type p110 or the corresponding dimerization mutant, respectively. Likewise, p150 and p150mut indicates editing levels of wild type p150 and the corresponding dimerization mutant. Data height and error bars correspond to the mean values and standard deviation of three biological replicates (n=3) for PEI, p110, p110mut and p150mut, or two biological replicates (n=2) for p150. Source data are provided as a Source Data file.

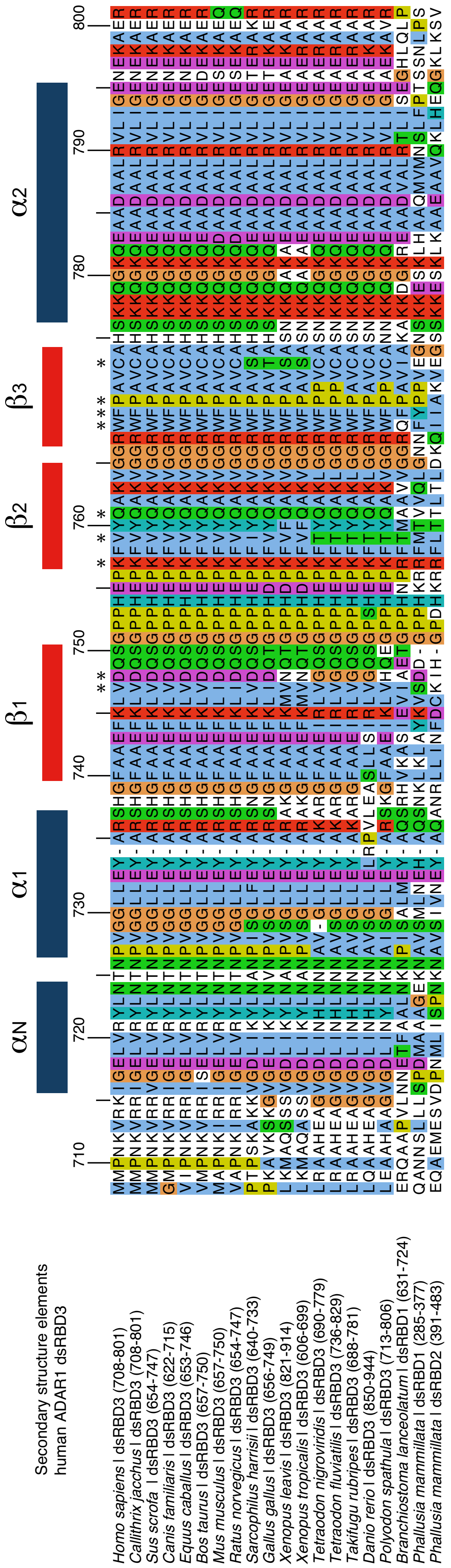

Figure S13: Dimerization of ADAR1-dsRBD3 is likely conserved throughout vertebrates. Sequence alignment of various ADAR1-dsRBD3 from *Homo sapiens, Callithrix jacchus, Sus scrofa, Canis familiaris, Equus caballus, Bos taurus, Mus musculus, Rattus norvegicus, Sarcophilus harrisii, Gallus gallus, Xenopus leavis, Xenopus tropicalis, Tetraodon nigroviridis, Tetraodon fluviatilis, Takifugu rubripes, Danio rerio,* and *Polyodon spathula*. In addition, sequences of ADAR's dsRBDs of *Branchiostoma lanceolatum* and *Phallusia mammillata*, that carry only one or two dsRBDs are shown. Alignment was done with ClustalW. For each sequence, the name of the species is given in the first item, the dsRBD's number in the second item, and the range of amino-acid composing the dsRBD in the numbering of the full-length protein in the third item. The alignment is colored by amino-acid conservation and properties. Human ADAR1-dsRBD3 amino-acid numbering and secondary structure elements are shown on top of the alignment. Residues at the dimerization interface are marked with an asterisk above the alignment. Key residues for dimerization are absolutely conserved in mammals, and almost entirely conserved (with only 1 or 2 conservative or semi-conservative mutations) in all vertebrates down to *Polyodon spathula*. Non vertebrate organisms belonging to the Cephalochordata and Tunicata subphylums, which have ADARs with only one or two dsRBDs, do not retain the identified elements involved in dimerization (more than 6 mutations at key positions, including W768 and Q761).
