# Supplementary figures and images for: Dimerization of ADAR1 modulates site-specificity of RNA editing
Source: Nat Commun. 2024 Nov 21;15:10051. doi: 10.1038/s41467-024-53777-2 (PMC11582362; doi:10.1038/s41467-024-53777-2)

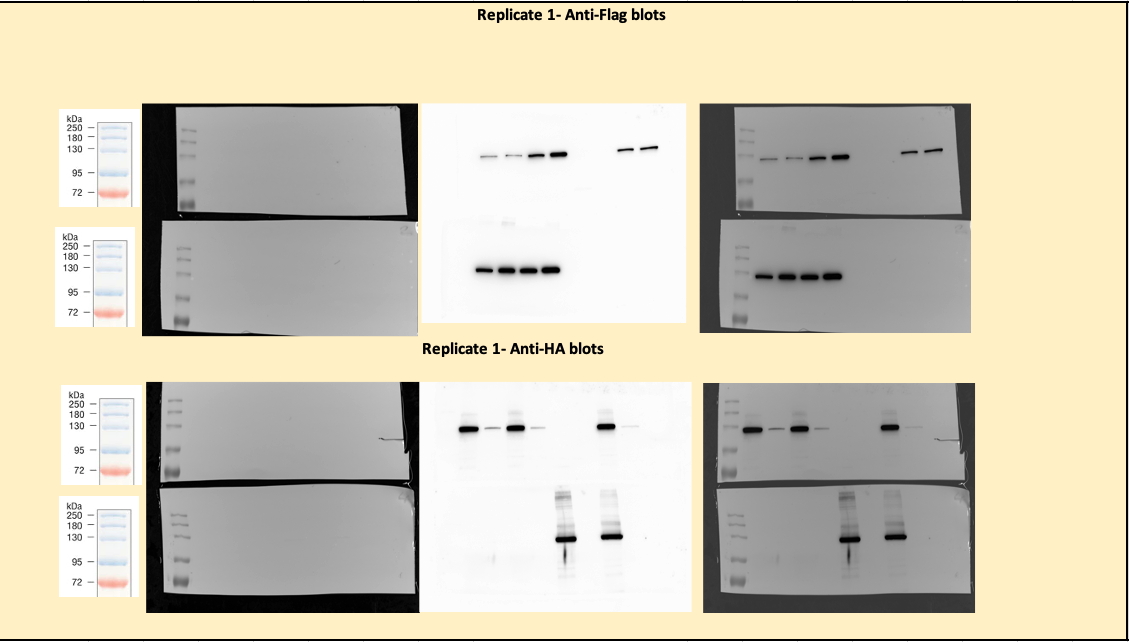

Supplement: Supplementary file 4 — Source data [file 41467_2024_53777_MOESM4_ESM.zip › Source-Data/Figure4/source-data-Figure4a-all-blots-with-MW-ladder.jpg]

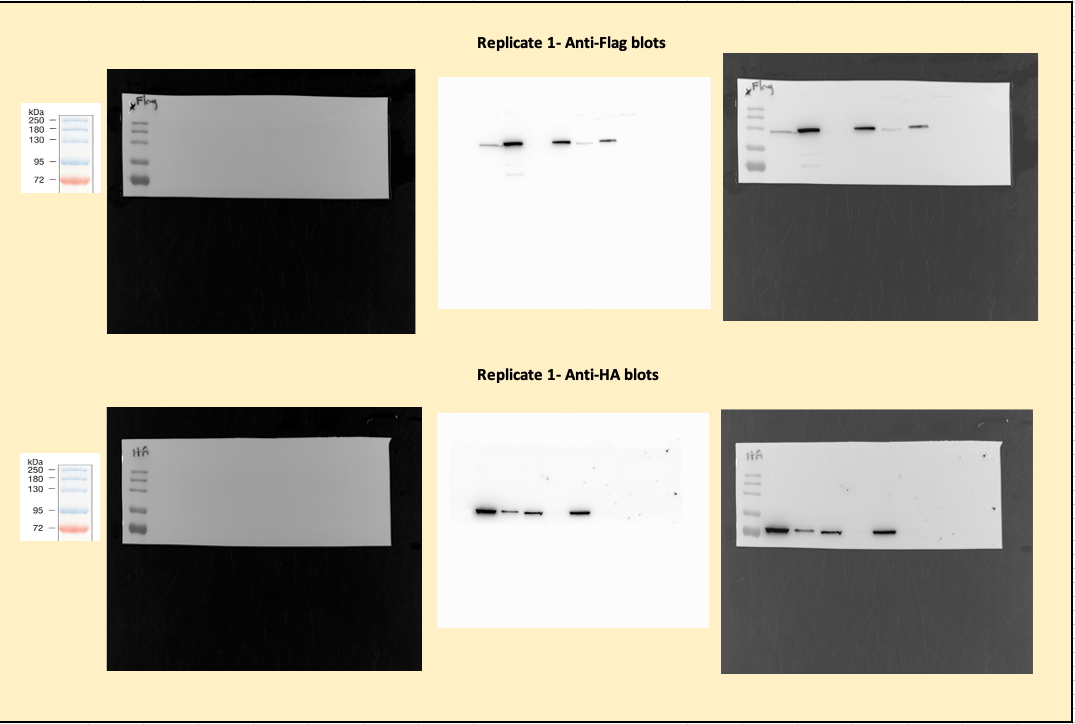

Supplement: Supplementary file 4 — Source data [file 41467_2024_53777_MOESM4_ESM.zip › Source-Data/Figure4/source-data-Figure4c-all-blots-with-MW-ladder.jpg]

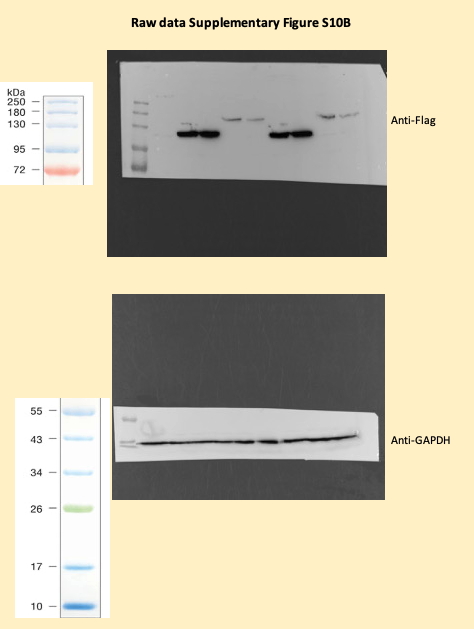

Supplement: Supplementary file 4 — Source data [file 41467_2024_53777_MOESM4_ESM.zip › Source-Data/FigureS10/source-data-Supplementary-Figure-S10B.jpg]

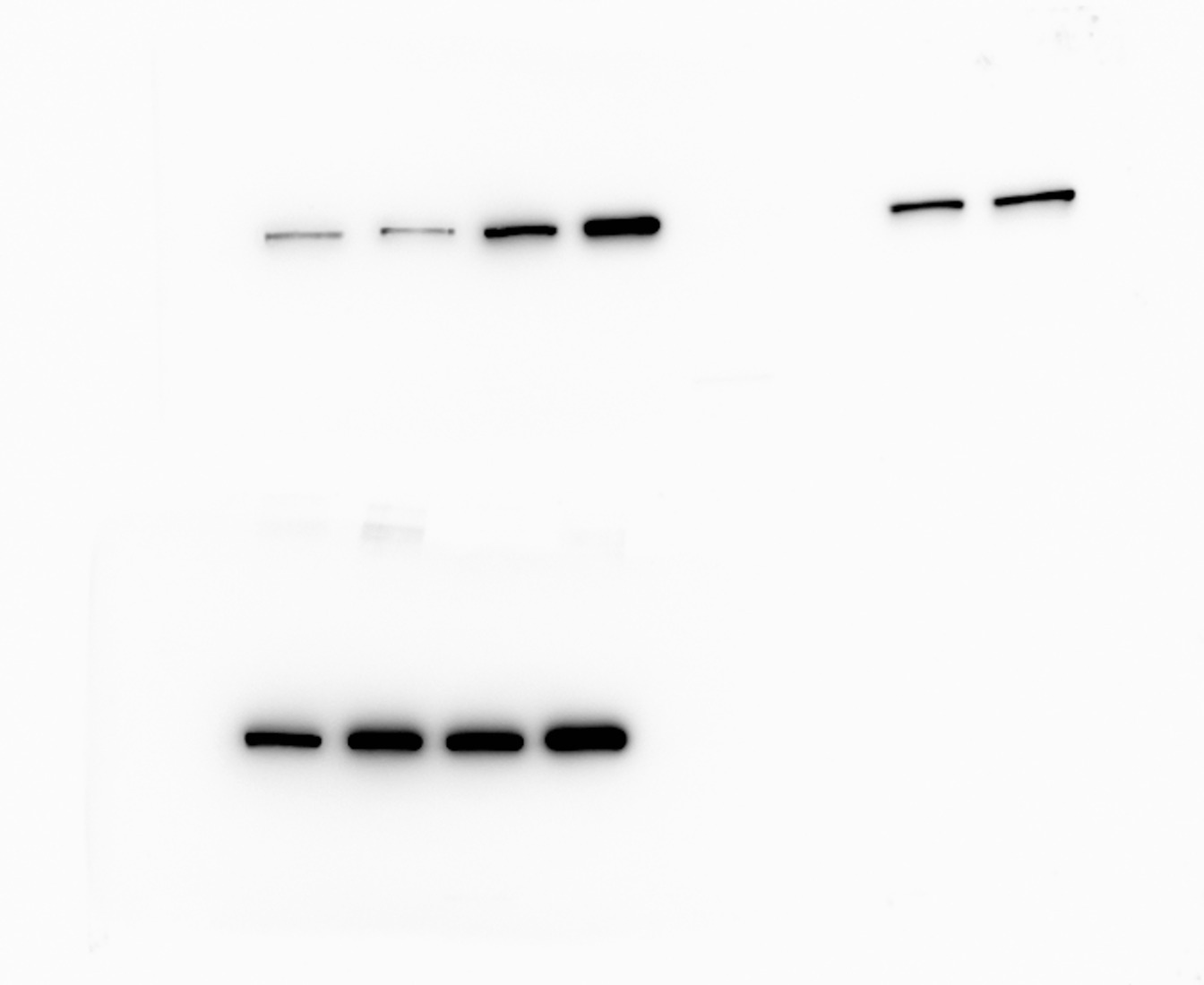

Supplement: Supplementary file 4 — Source data [file 41467_2024_53777_MOESM4_ESM.zip › Source-Data/Figure4/Raw-data-Figure-4a/Replicate_1_Flag_Bands.jpg]

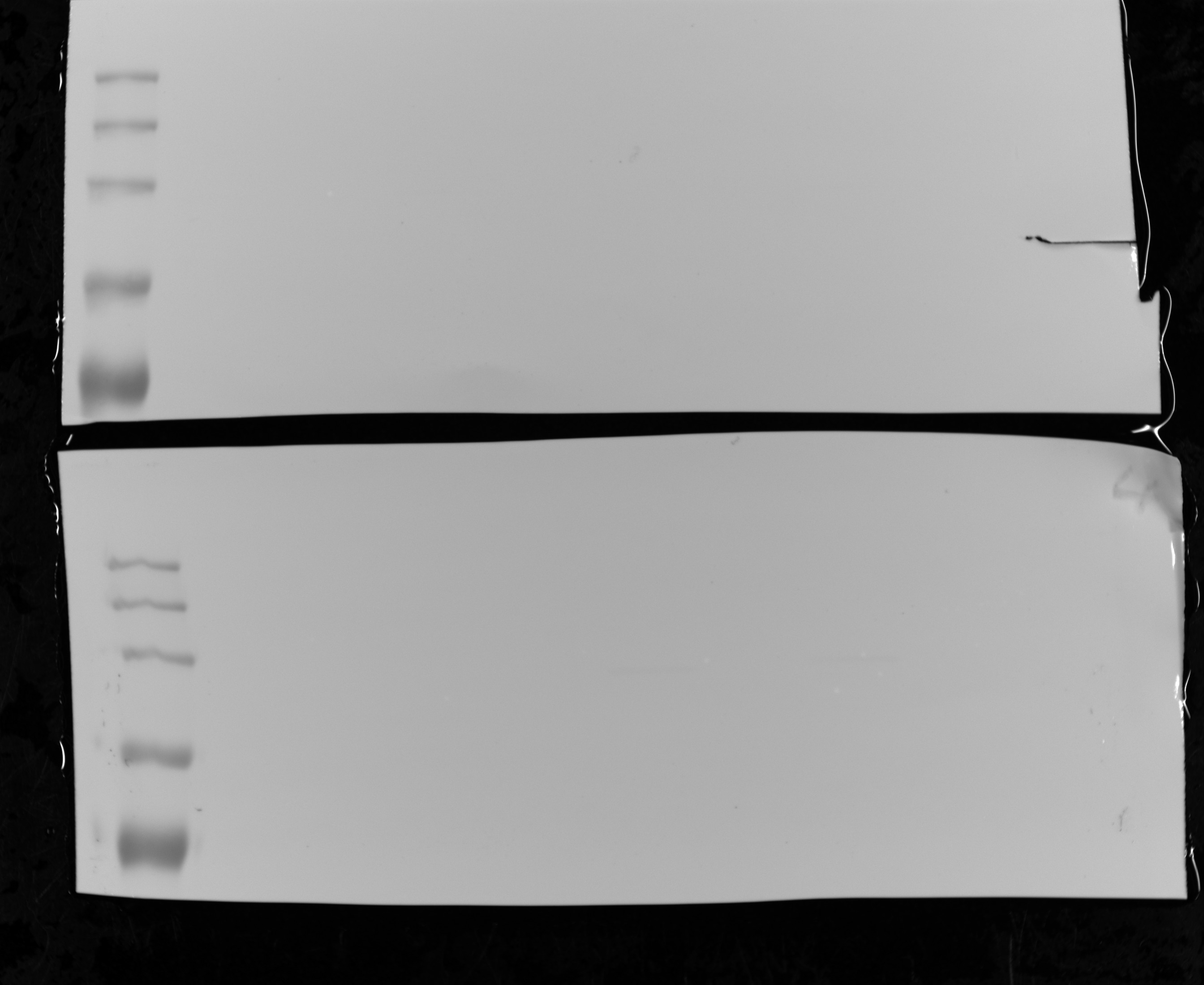

Supplement: Supplementary file 4 — Source data [file 41467_2024_53777_MOESM4_ESM.zip › Source-Data/Figure4/Raw-data-Figure-4a/Replicate_1_HA_Marker.jpg]

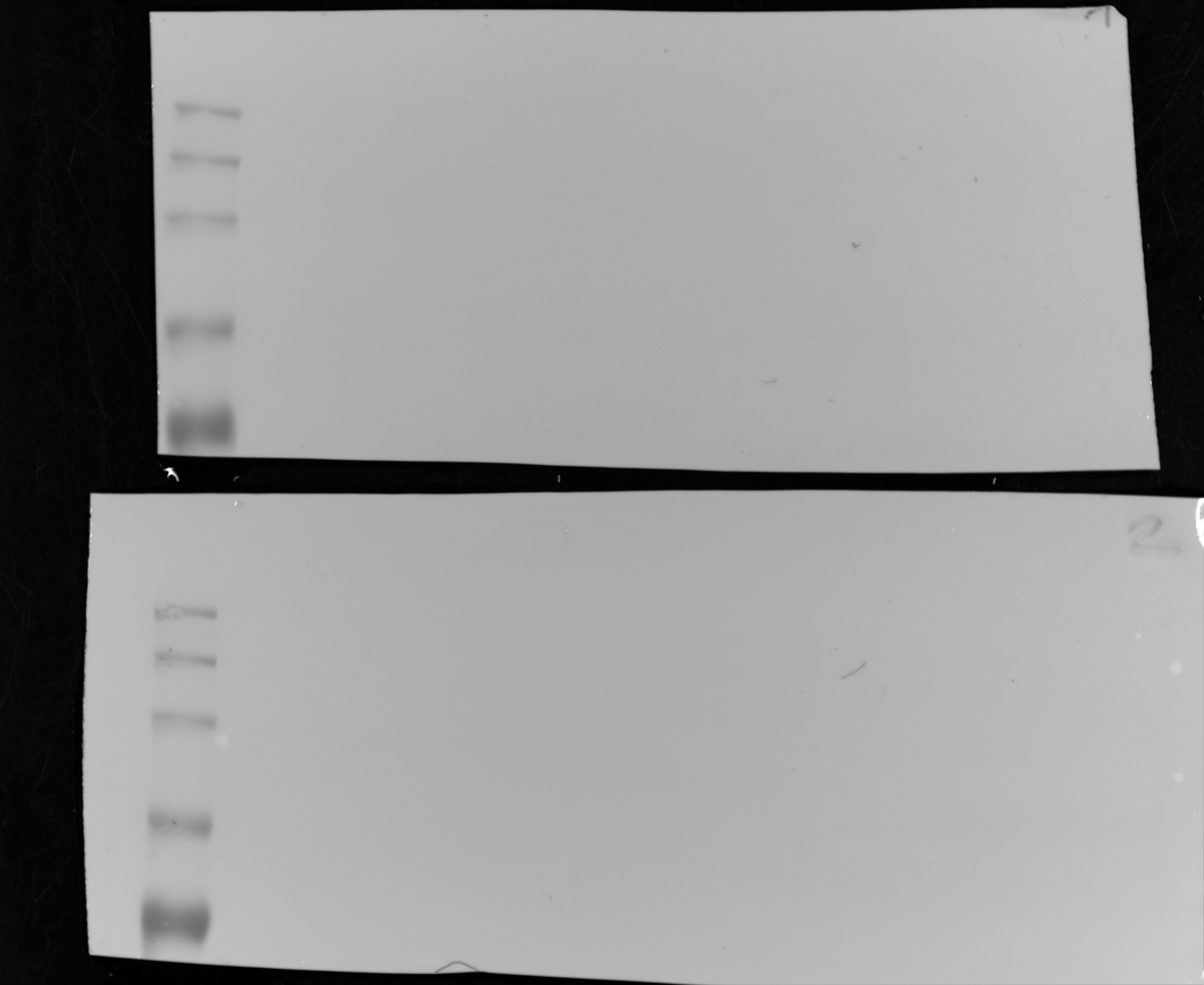

Supplement: Supplementary file 4 — Source data [file 41467_2024_53777_MOESM4_ESM.zip › Source-Data/Figure4/Raw-data-Figure-4a/Replicate_1_Flag_Marker.jpg]

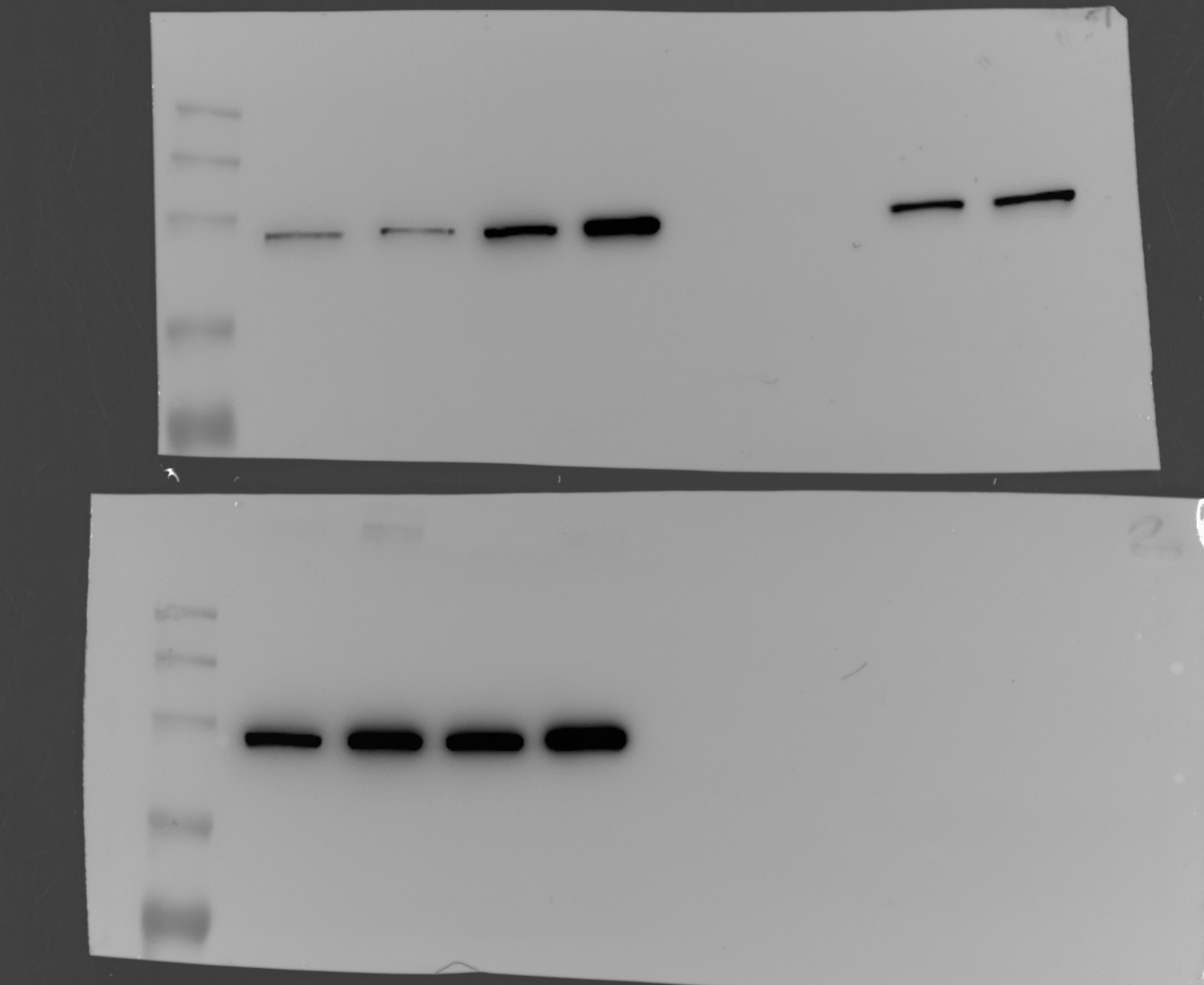

Supplement: Supplementary file 4 — Source data [file 41467_2024_53777_MOESM4_ESM.zip › Source-Data/Figure4/Raw-data-Figure-4a/Replicate_1_Flag_Merge.jpg]

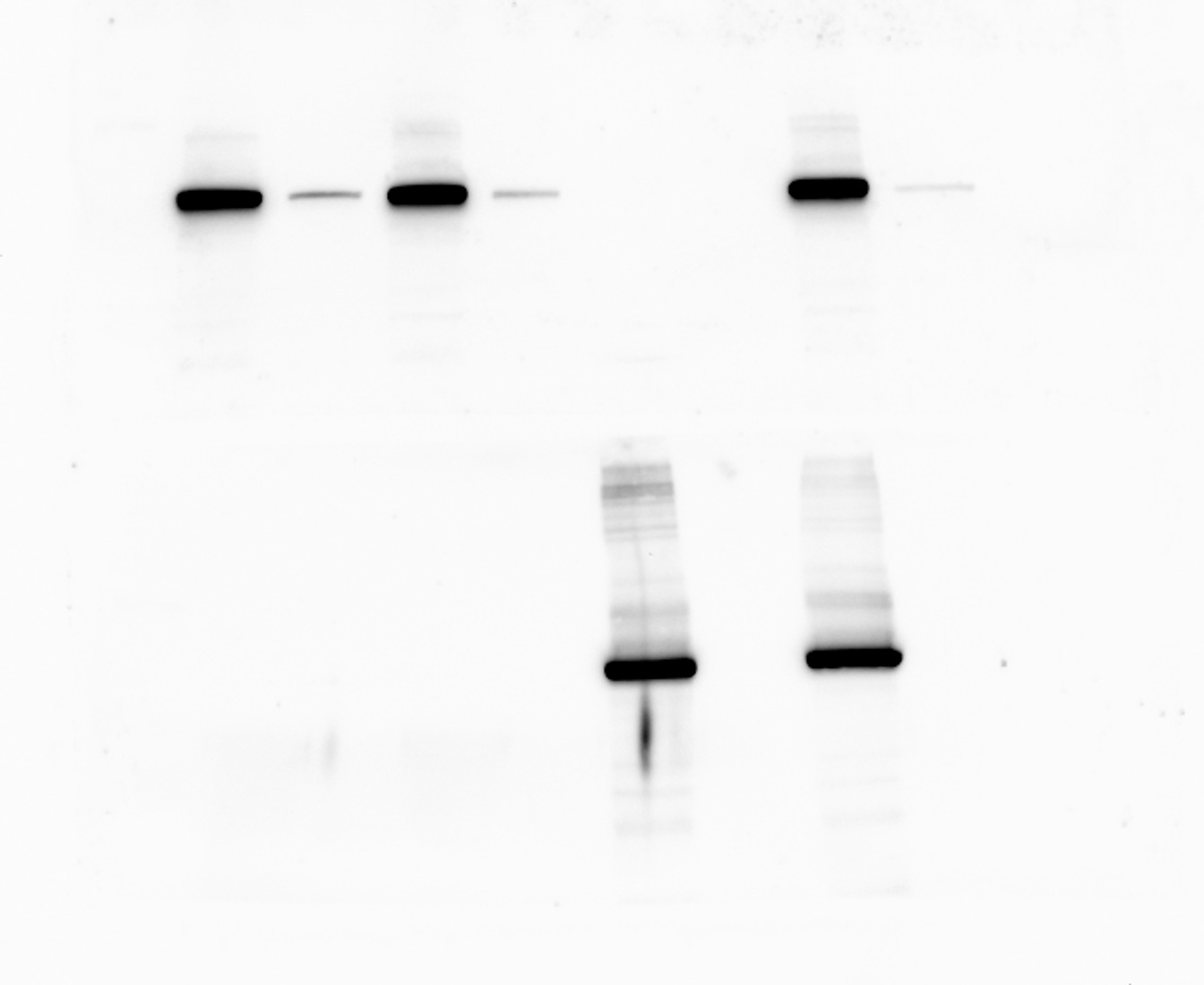

Supplement: Supplementary file 4 — Source data [file 41467_2024_53777_MOESM4_ESM.zip › Source-Data/Figure4/Raw-data-Figure-4a/Replicate_1_HA_Bands.jpg]

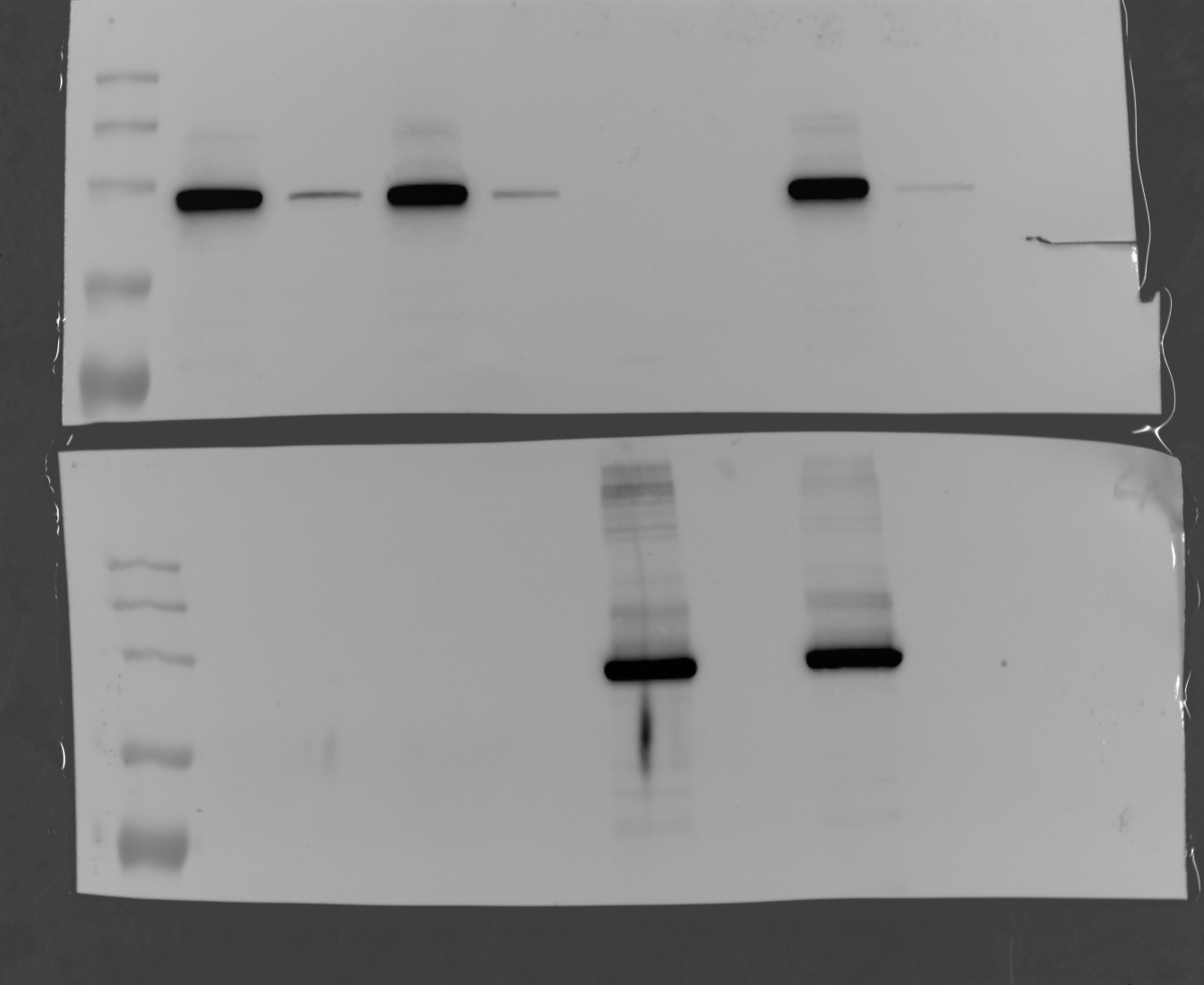

Supplement: Supplementary file 4 — Source data [file 41467_2024_53777_MOESM4_ESM.zip › Source-Data/Figure4/Raw-data-Figure-4a/Replicate_1_HA_Merge.jpg]

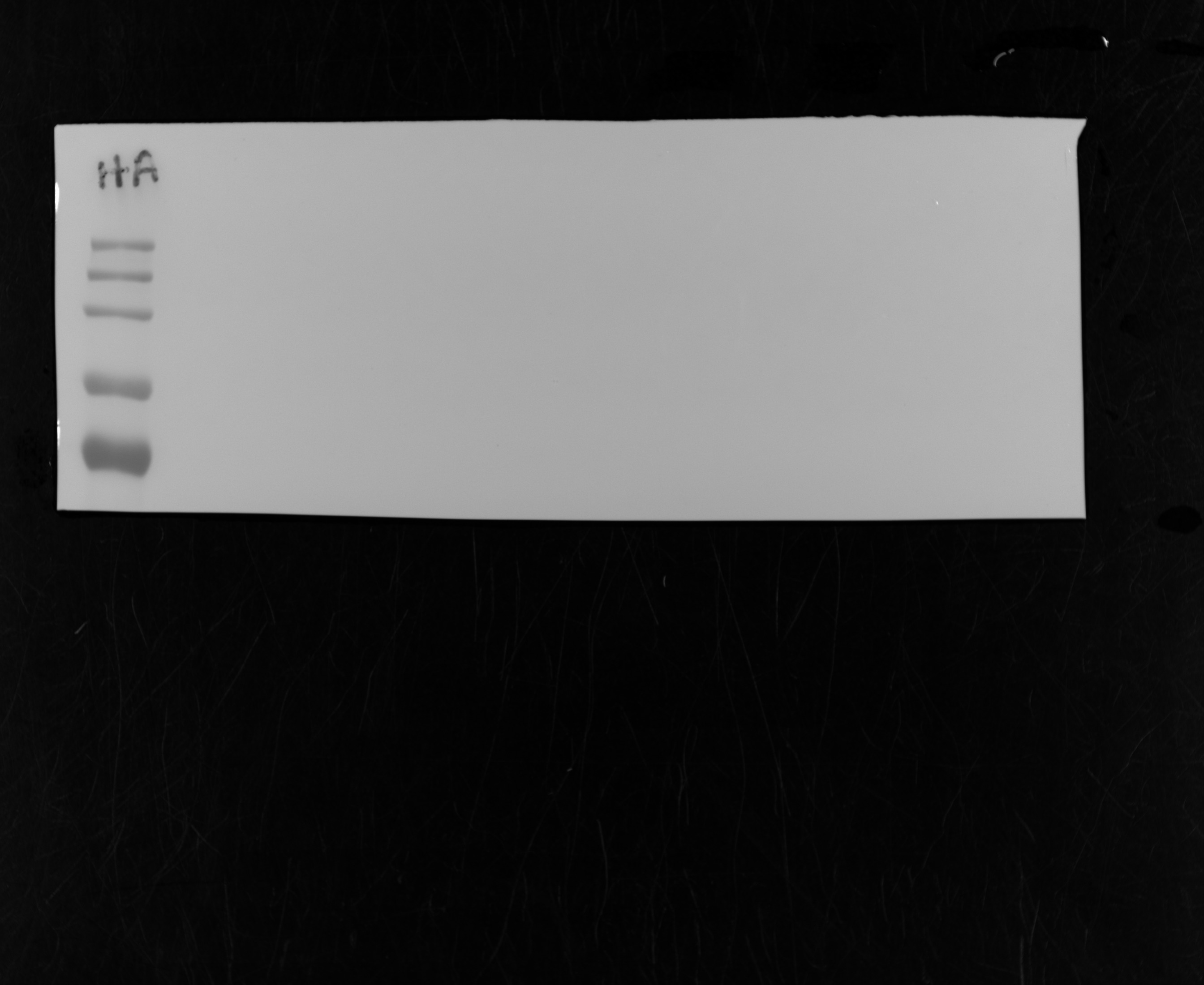

Supplement: Supplementary file 4 — Source data [file 41467_2024_53777_MOESM4_ESM.zip › Source-Data/Figure4/Raw-data-Figure-4c/Replicate_1_HA-delta-deaminase_Marker.jpg]

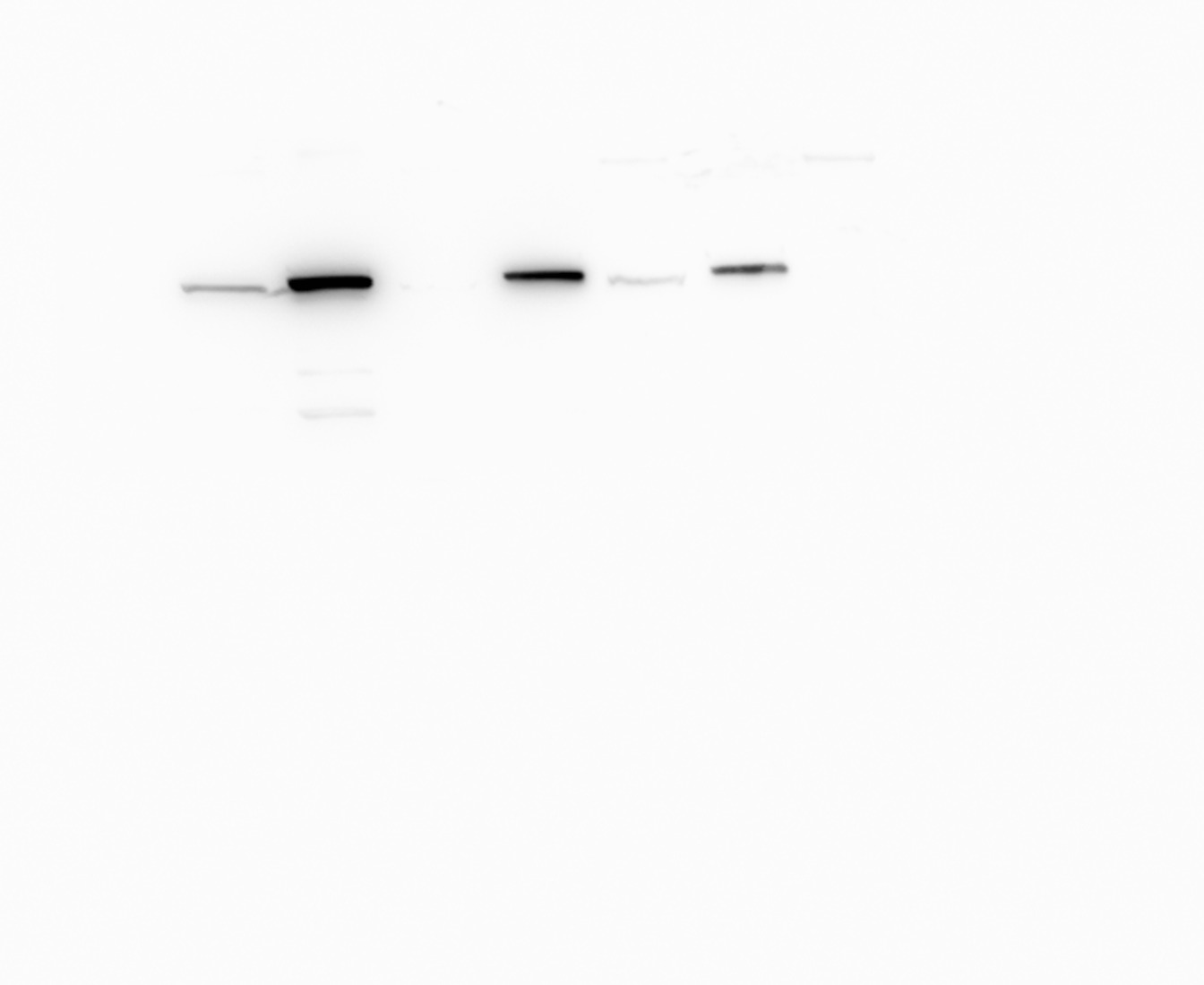

Supplement: Supplementary file 4 — Source data [file 41467_2024_53777_MOESM4_ESM.zip › Source-Data/Figure4/Raw-data-Figure-4c/Replicate_1_Flag_Bands.jpg]

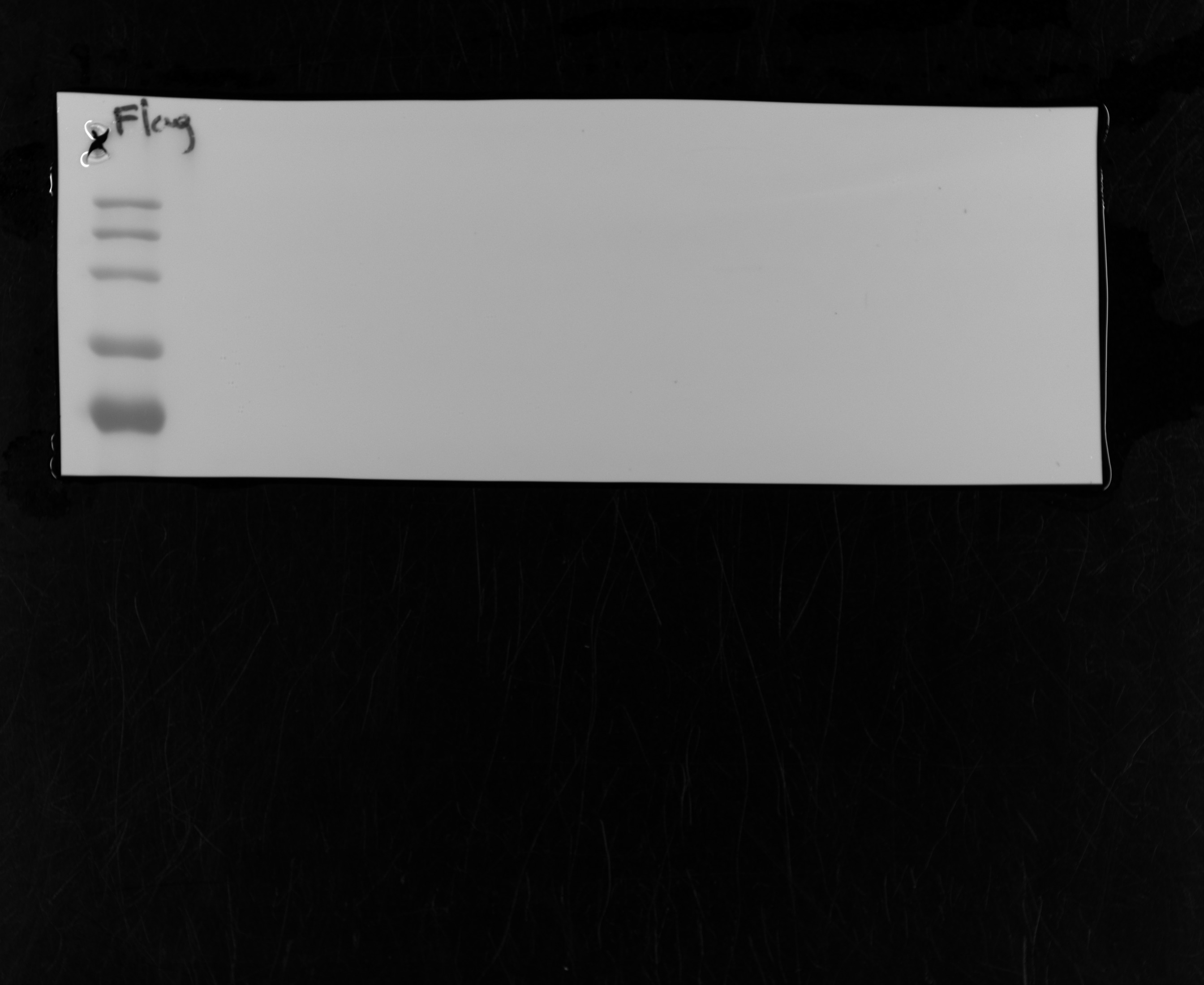

Supplement: Supplementary file 4 — Source data [file 41467_2024_53777_MOESM4_ESM.zip › Source-Data/Figure4/Raw-data-Figure-4c/Replicate_1_Flag_Marker.jpg]

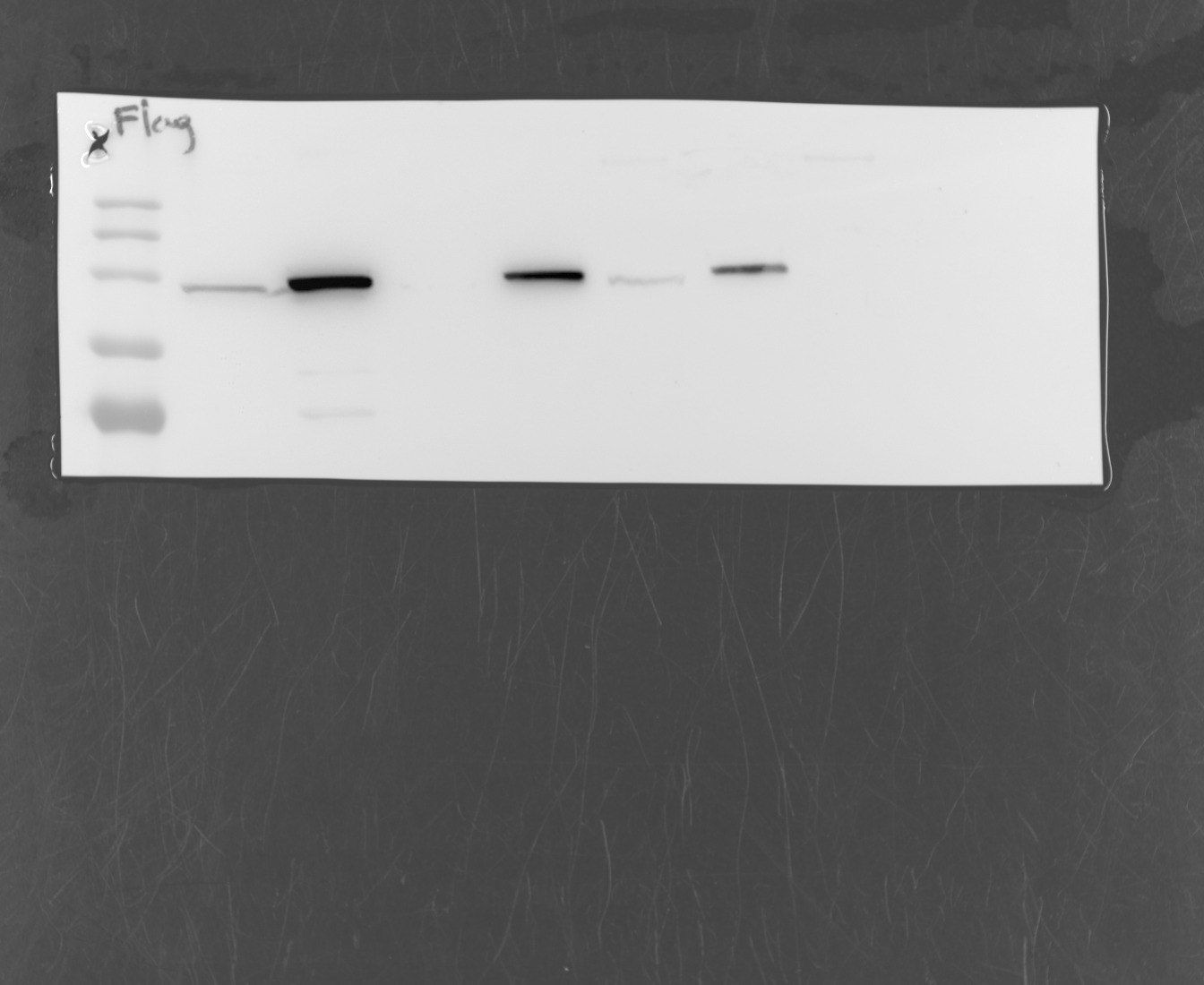

Supplement: Supplementary file 4 — Source data [file 41467_2024_53777_MOESM4_ESM.zip › Source-Data/Figure4/Raw-data-Figure-4c/Replicate_1_Flag_Merge.jpg]

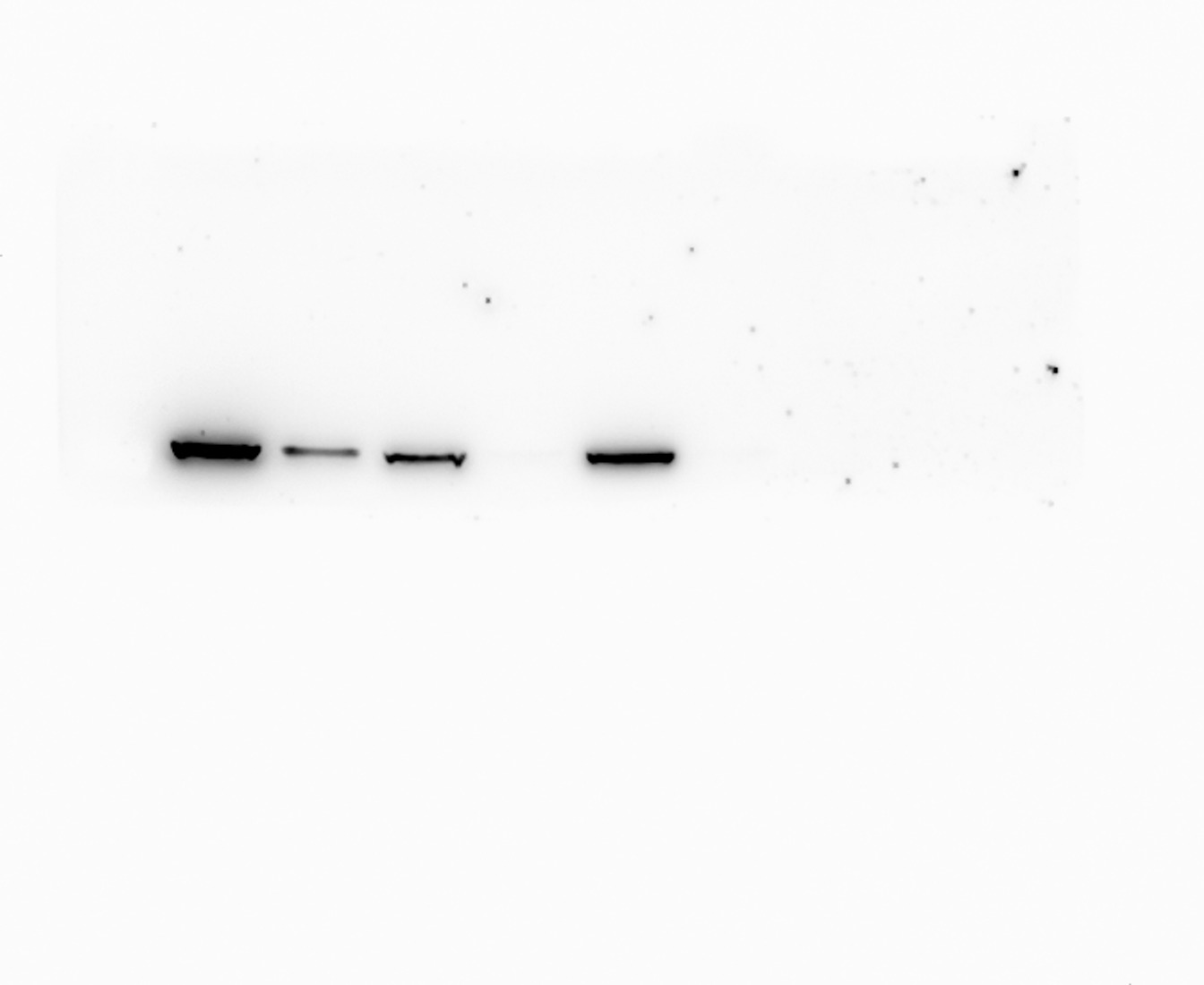

Supplement: Supplementary file 4 — Source data [file 41467_2024_53777_MOESM4_ESM.zip › Source-Data/Figure4/Raw-data-Figure-4c/Replicate_1_HA-delta-deaminase_Bands.jpg]

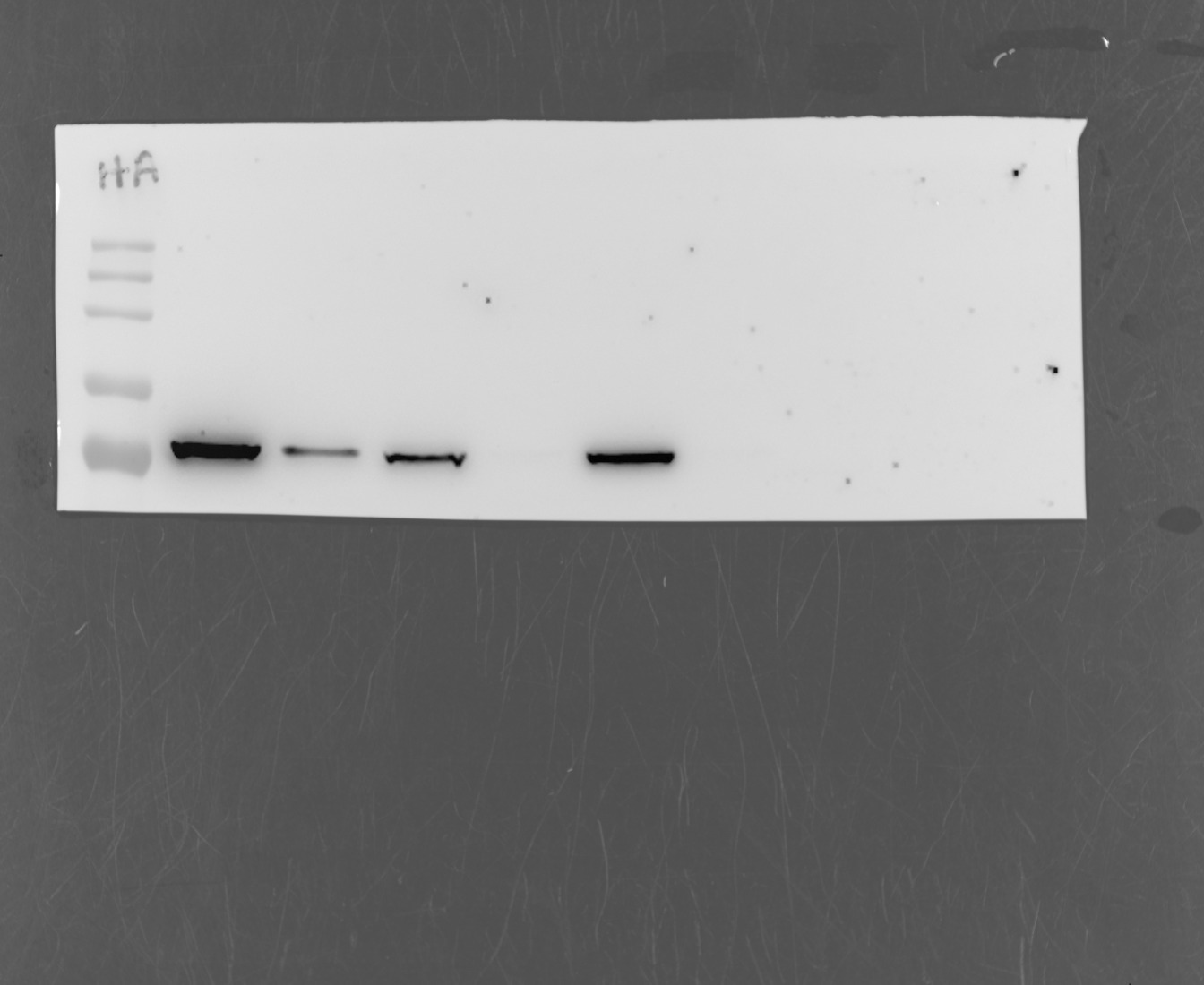

Supplement: Supplementary file 4 — Source data [file 41467_2024_53777_MOESM4_ESM.zip › Source-Data/Figure4/Raw-data-Figure-4c/Replicate_1_HA-delta-deaminase_Merge.jpg]

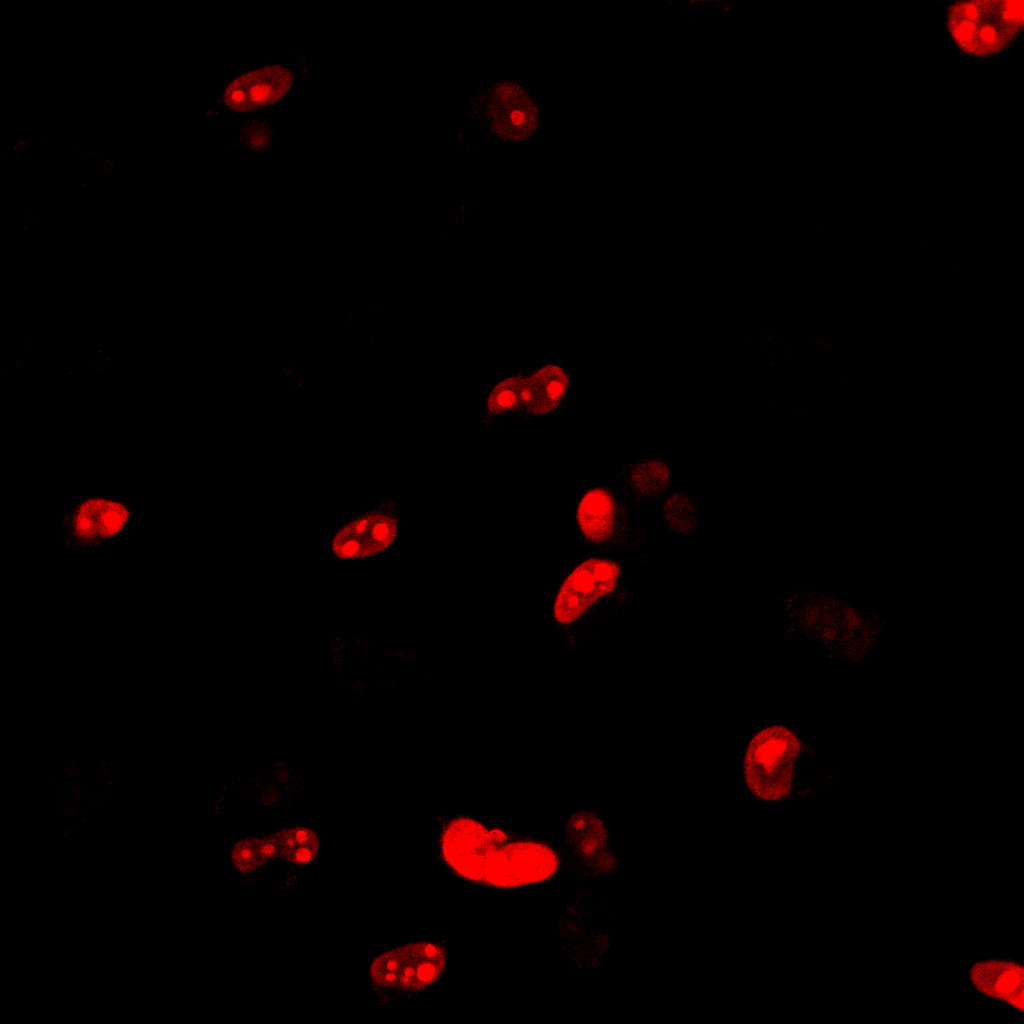

Supplement: Supplementary file 4 — Source data [file 41467_2024_53777_MOESM4_ESM.zip › Source-Data/FigureS10/source-data-Supplementary-Figure-S10A/Flag_p110dsRBD3-interface-mutant_TritC.jpg]

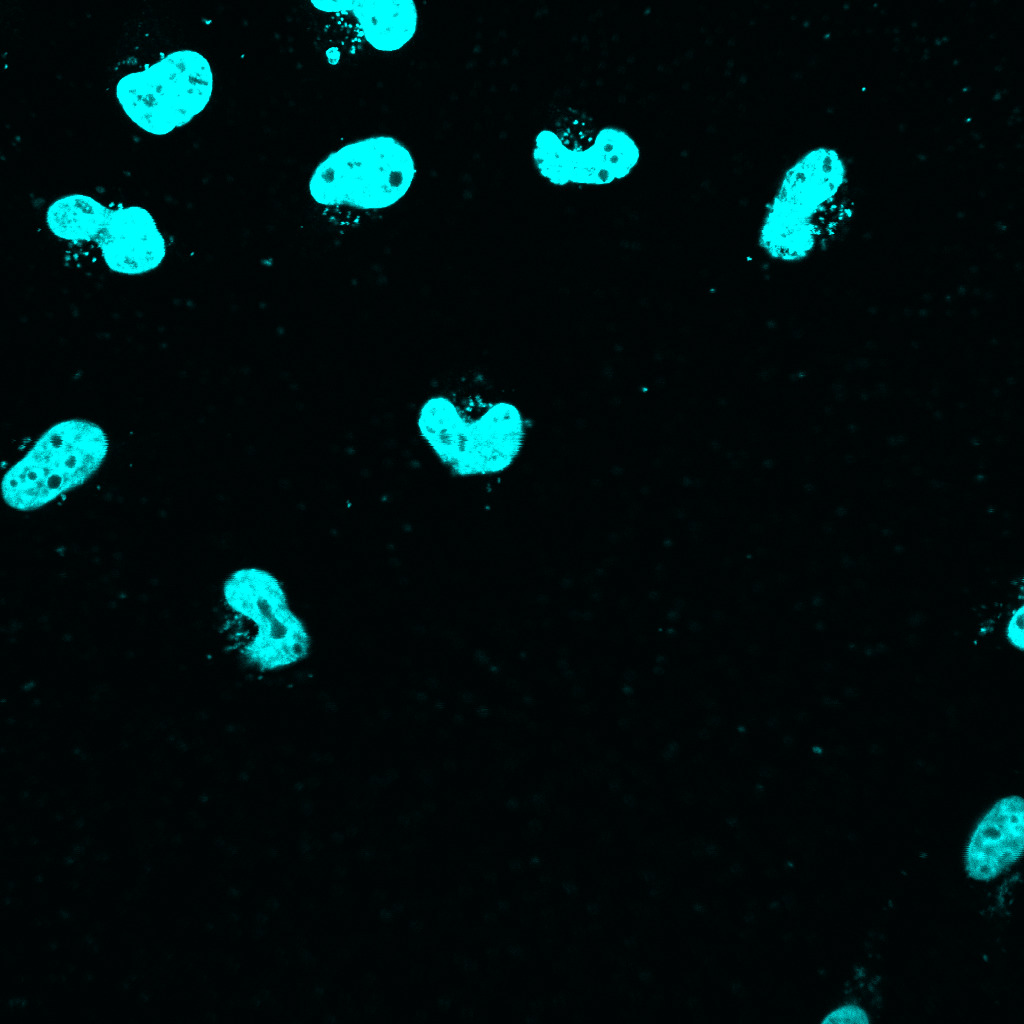

Supplement: Supplementary file 4 — Source data [file 41467_2024_53777_MOESM4_ESM.zip › Source-Data/FigureS10/source-data-Supplementary-Figure-S10A/HA_p110-delta-deaminase_DAPI.jpg]

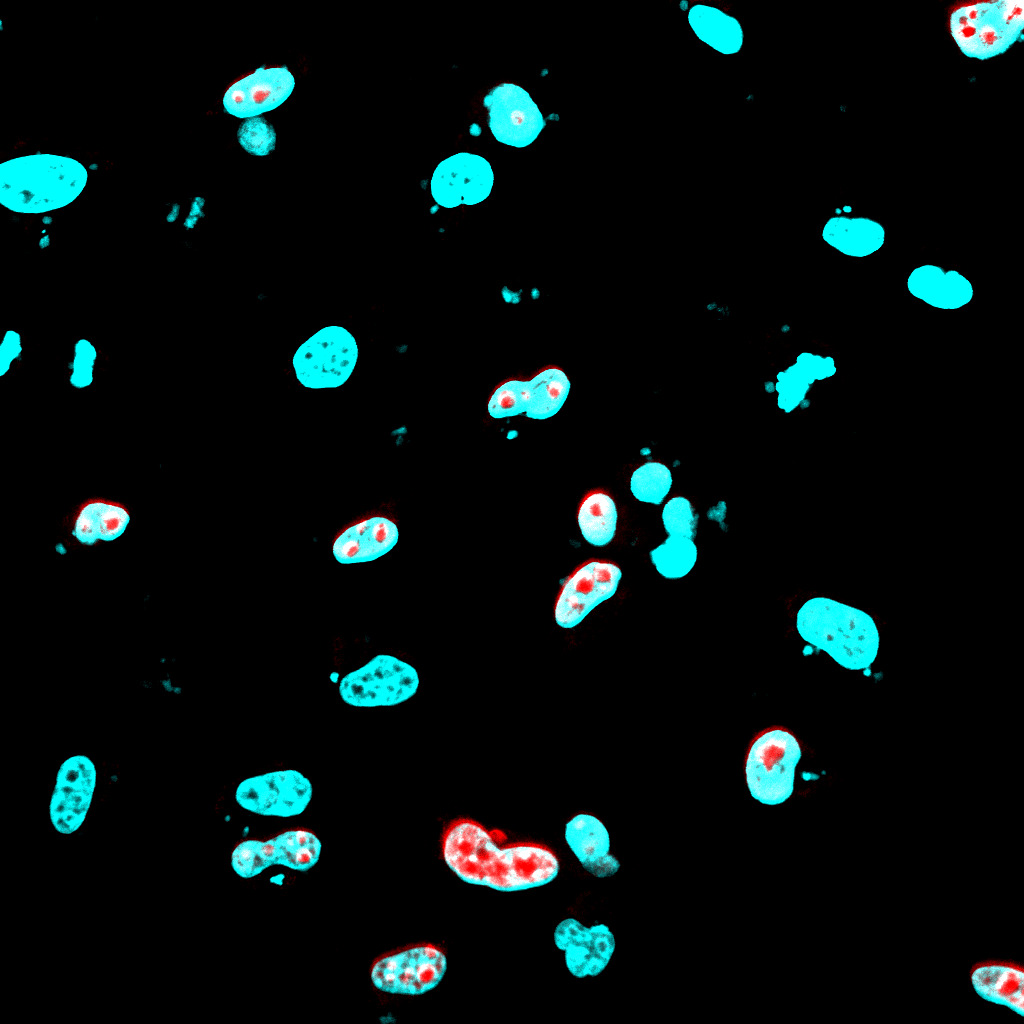

Supplement: Supplementary file 4 — Source data [file 41467_2024_53777_MOESM4_ESM.zip › Source-Data/FigureS10/source-data-Supplementary-Figure-S10A/Flag_p110dsRBD3-interface-mutant_merge.jpg]

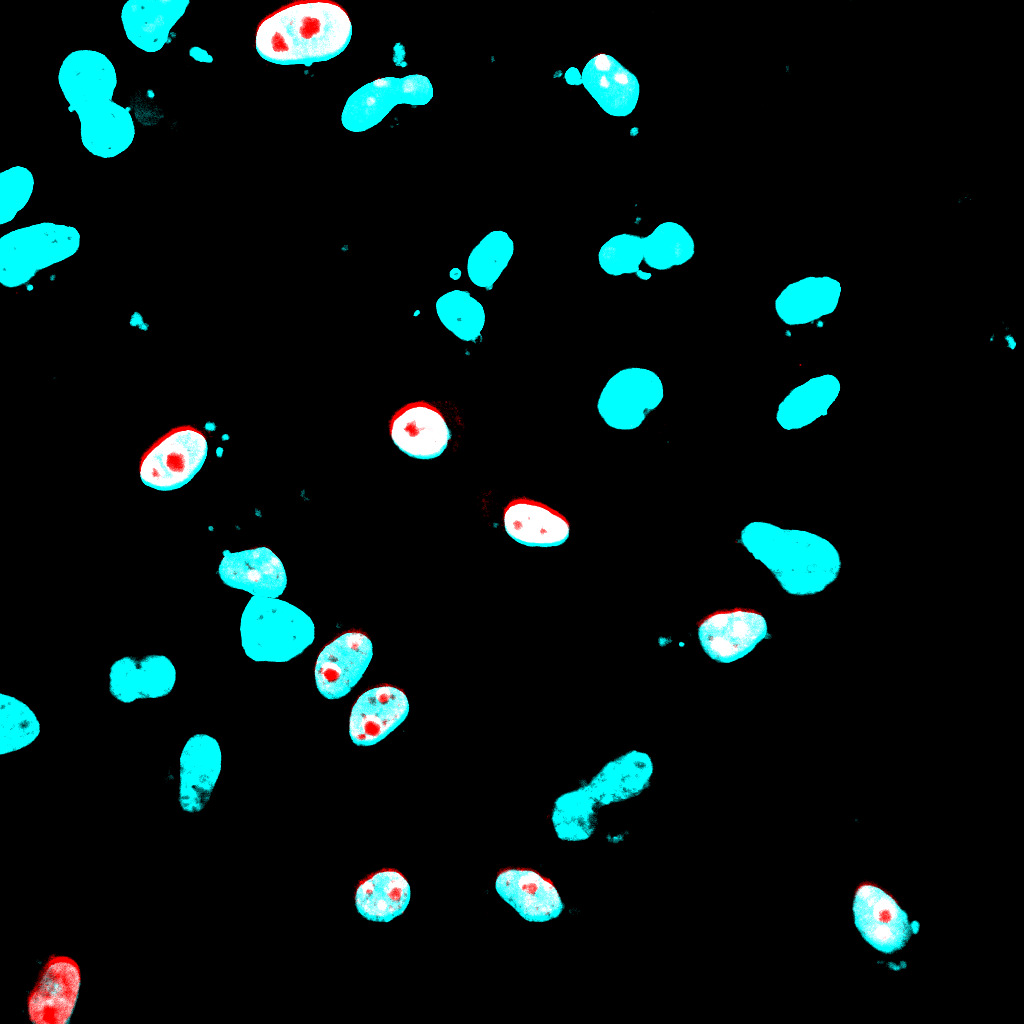

Supplement: Supplementary file 4 — Source data [file 41467_2024_53777_MOESM4_ESM.zip › Source-Data/FigureS10/source-data-Supplementary-Figure-S10A/Flag_p110_merge.jpg]

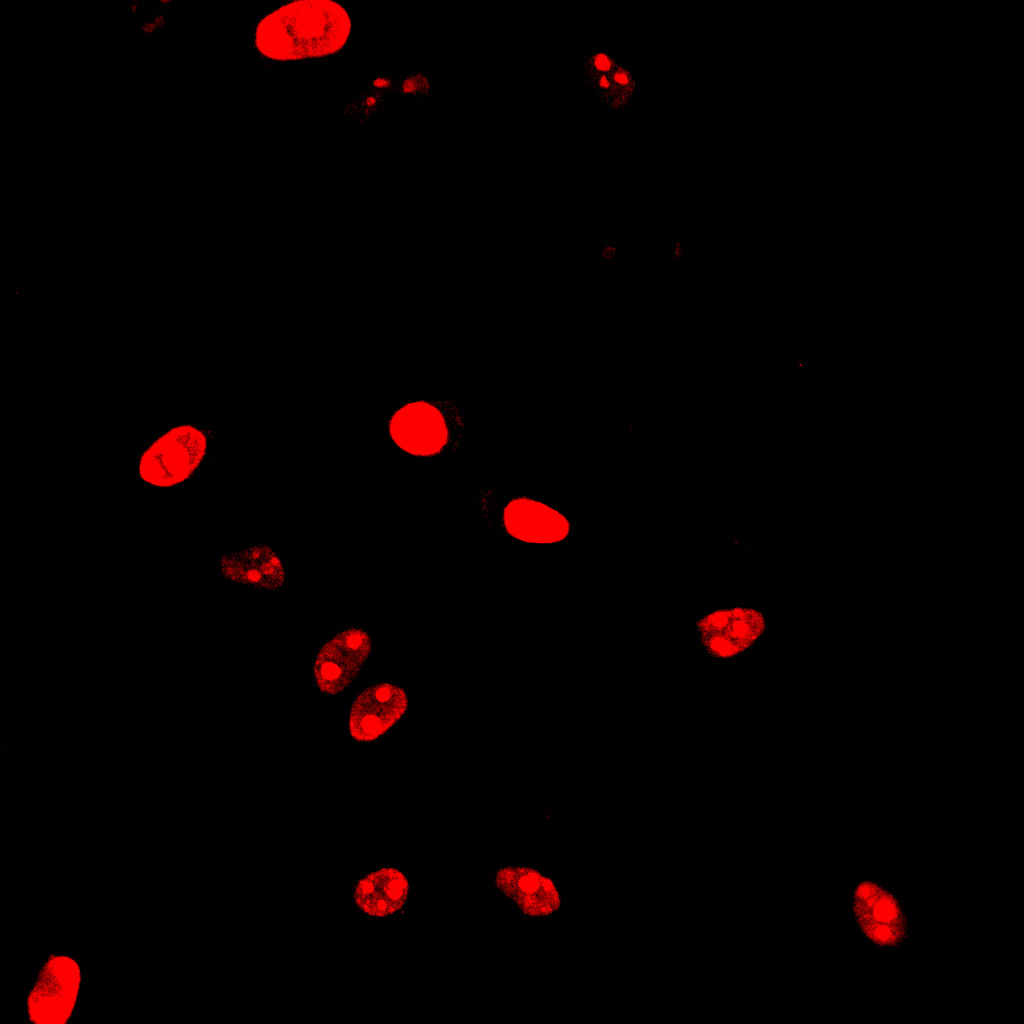

Supplement: Supplementary file 4 — Source data [file 41467_2024_53777_MOESM4_ESM.zip › Source-Data/FigureS10/source-data-Supplementary-Figure-S10A/Flag_p110_TritC.jpg]

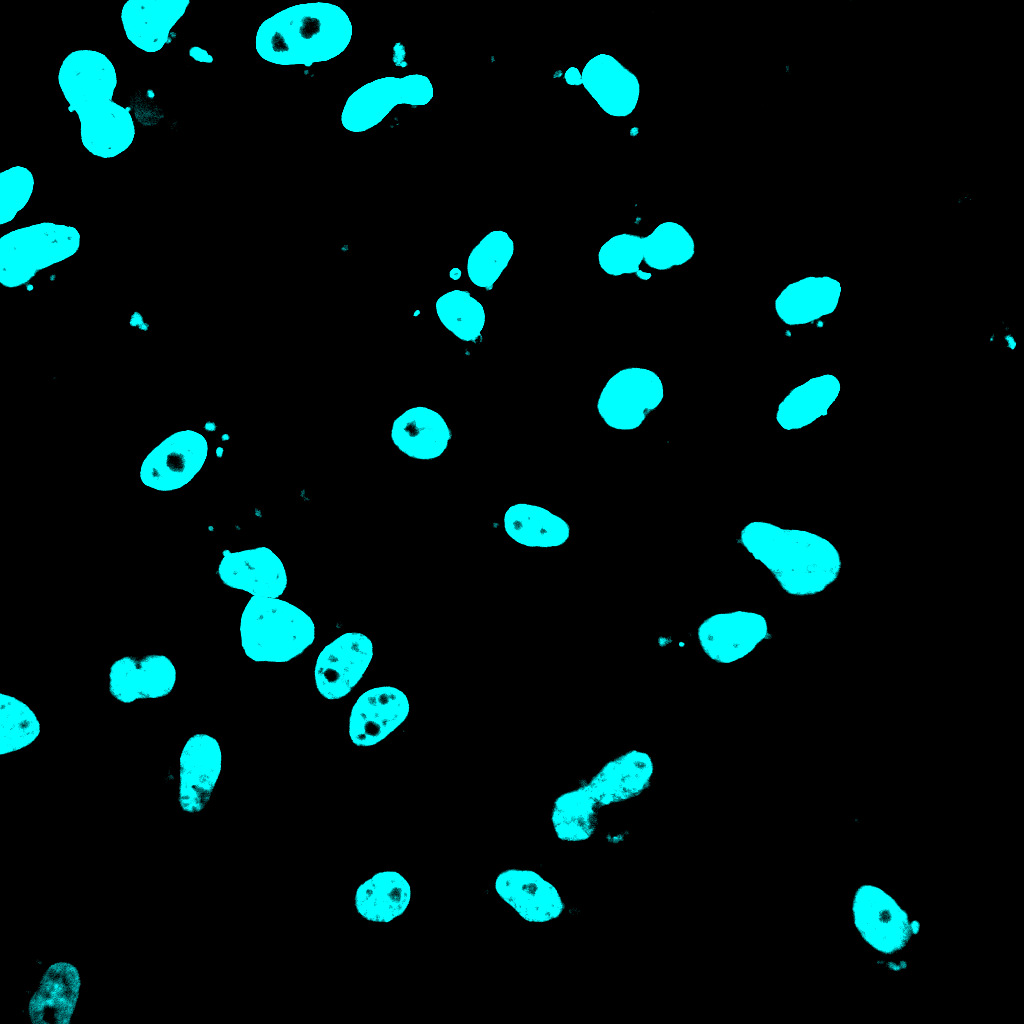

Supplement: Supplementary file 4 — Source data [file 41467_2024_53777_MOESM4_ESM.zip › Source-Data/FigureS10/source-data-Supplementary-Figure-S10A/Flag_p110_DAPI.jpg]

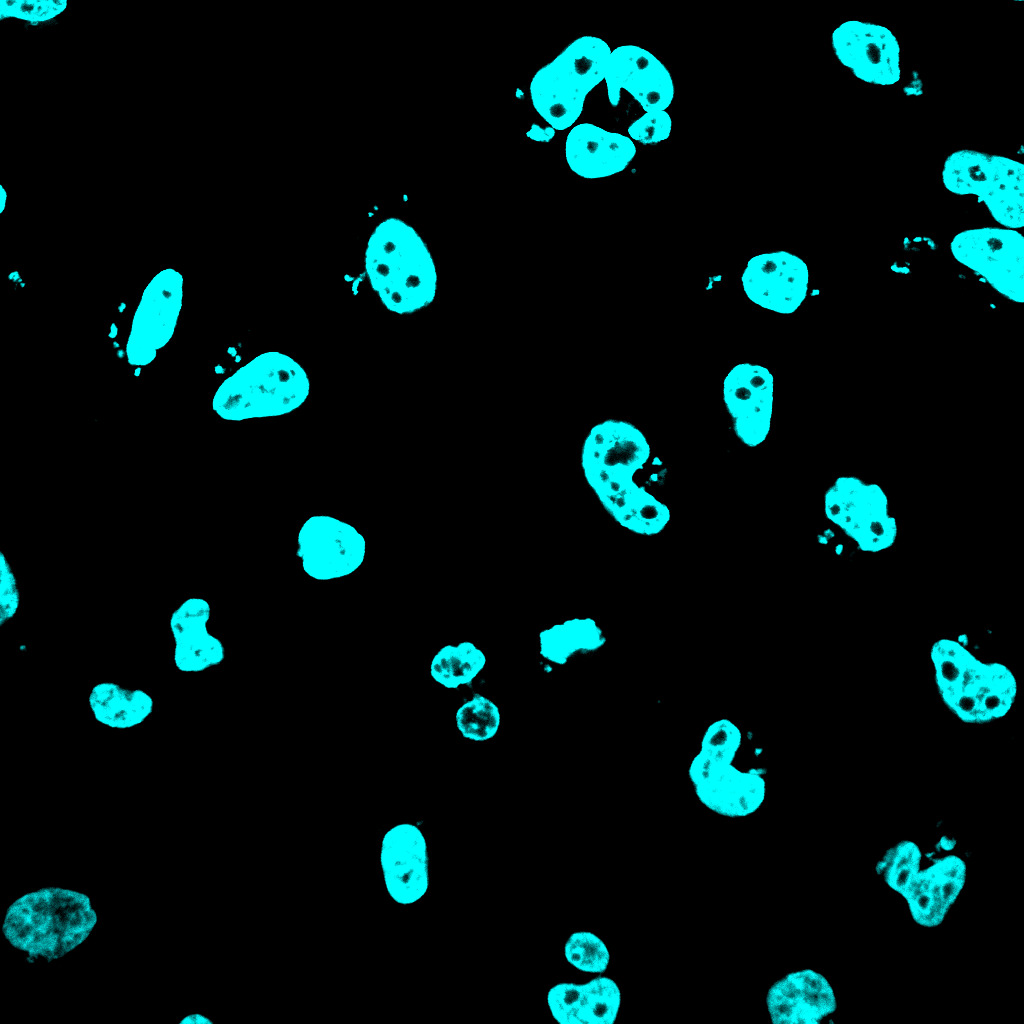

Supplement: Supplementary file 4 — Source data [file 41467_2024_53777_MOESM4_ESM.zip › Source-Data/FigureS10/source-data-Supplementary-Figure-S10A/HA_p110dsRBD3-interface-mutant-delta-deaminase_DAPI.jpg]

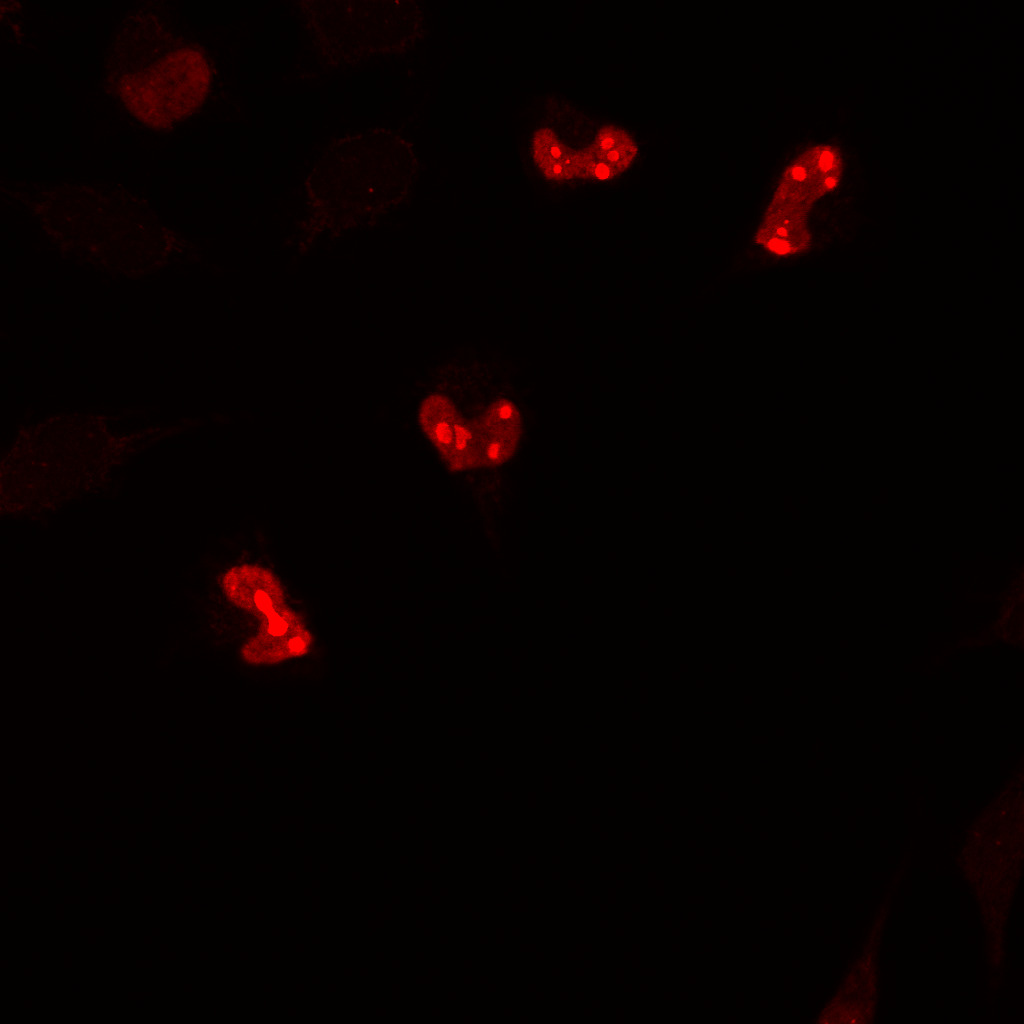

Supplement: Supplementary file 4 — Source data [file 41467_2024_53777_MOESM4_ESM.zip › Source-Data/FigureS10/source-data-Supplementary-Figure-S10A/HA_p110-delta-deaminase_TritC.jpg]

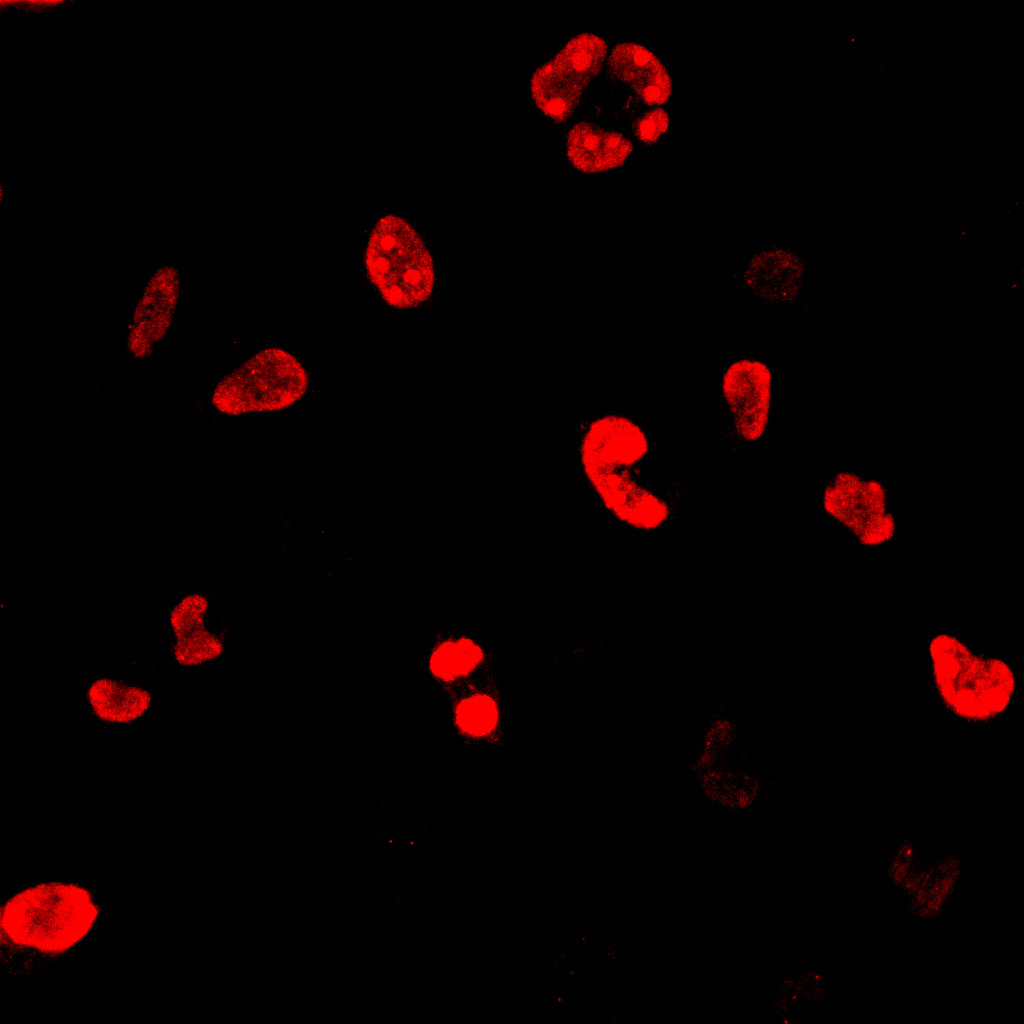

Supplement: Supplementary file 4 — Source data [file 41467_2024_53777_MOESM4_ESM.zip › Source-Data/FigureS10/source-data-Supplementary-Figure-S10A/HA_p110dsRBD3-interface-mutant-delta-deaminase_TritC.jpg]

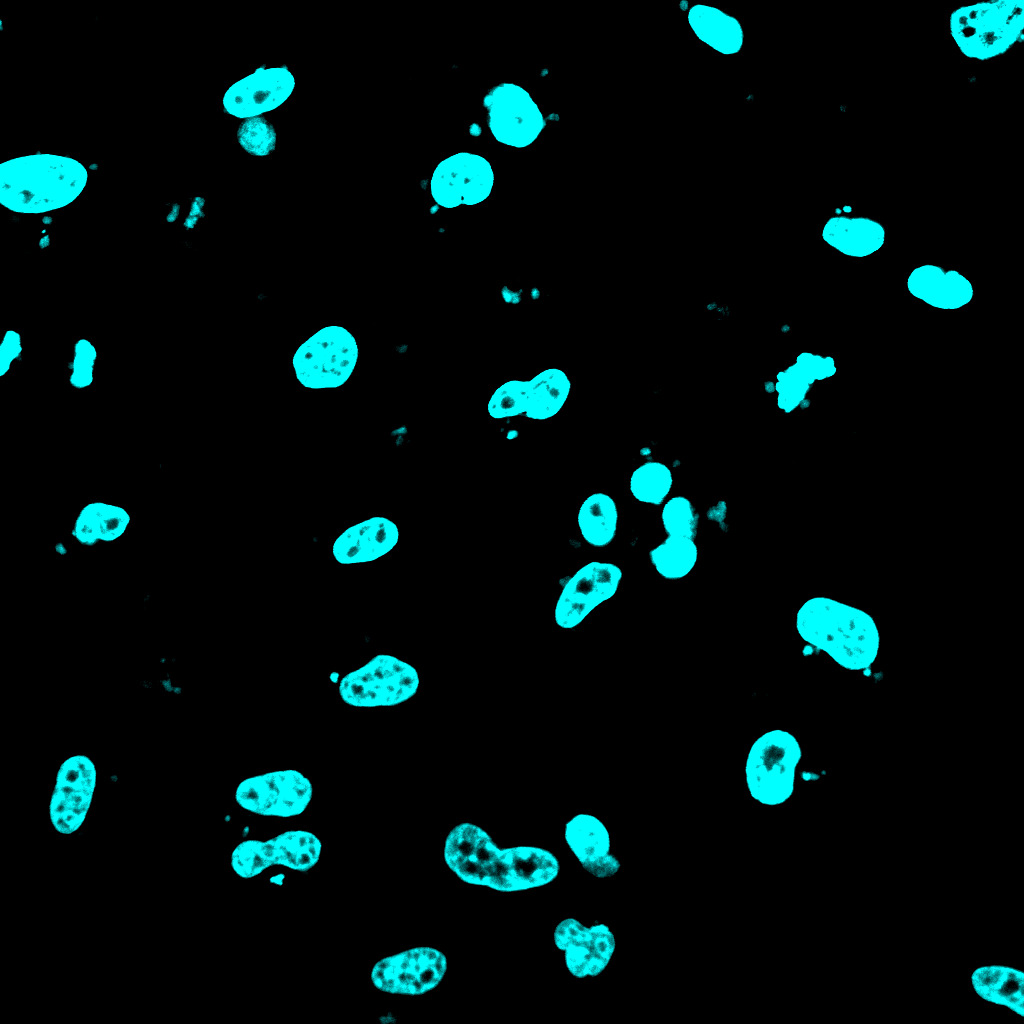

Supplement: Supplementary file 4 — Source data [file 41467_2024_53777_MOESM4_ESM.zip › Source-Data/FigureS10/source-data-Supplementary-Figure-S10A/Flag_p110dsRBD3-interface-mutant_DAPI.jpg]

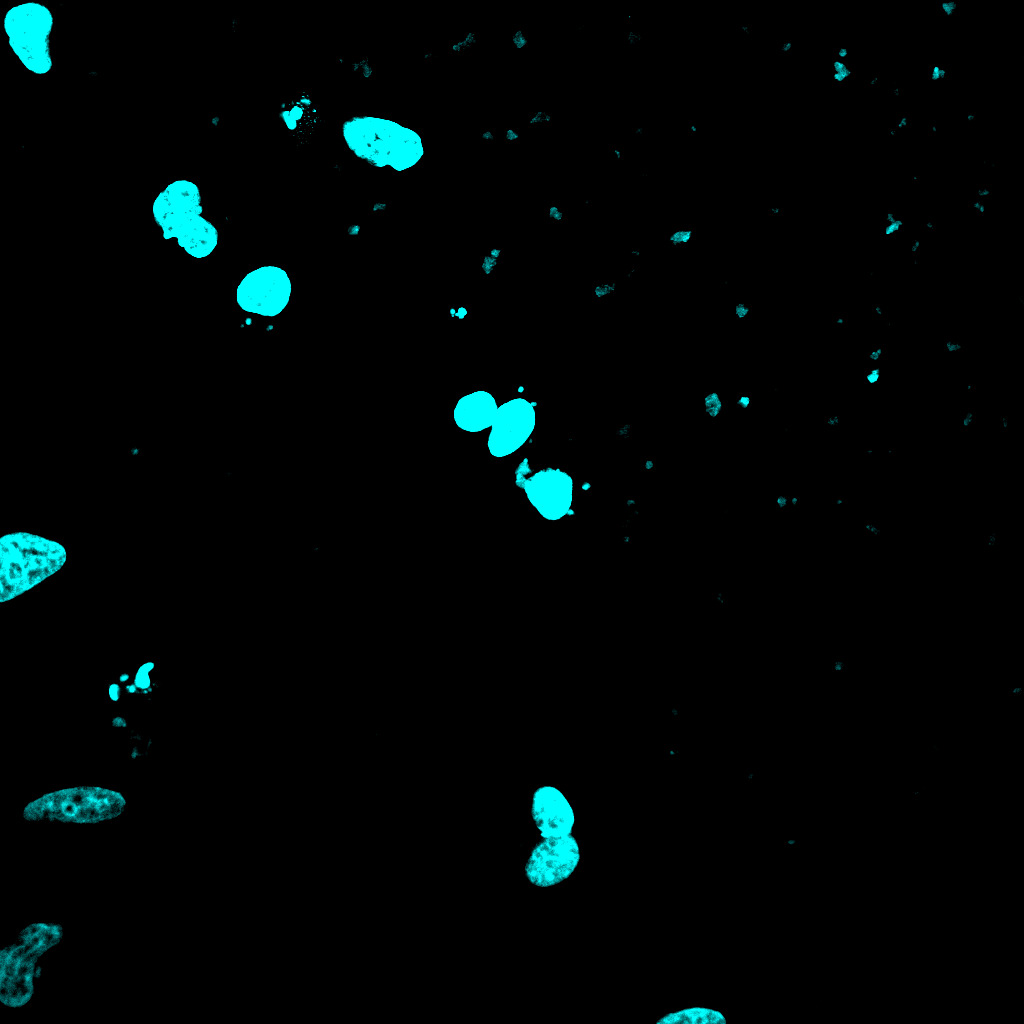

Supplement: Supplementary file 4 — Source data [file 41467_2024_53777_MOESM4_ESM.zip › Source-Data/FigureS10/source-data-Supplementary-Figure-S10A/Flag_p150dsRBD3-interface-mutant_DAPI.jpg]

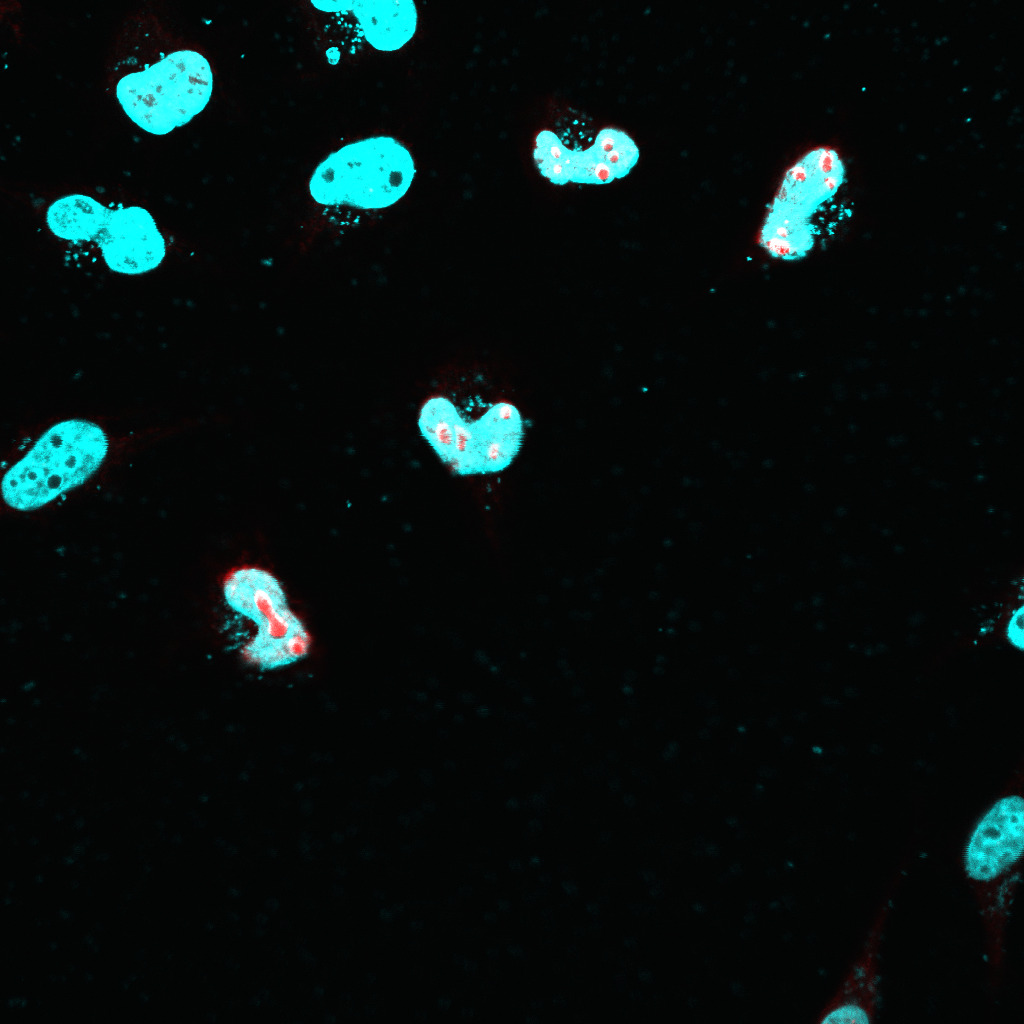

Supplement: Supplementary file 4 — Source data [file 41467_2024_53777_MOESM4_ESM.zip › Source-Data/FigureS10/source-data-Supplementary-Figure-S10A/HA_p110-delta-deaminase_merge.jpg]

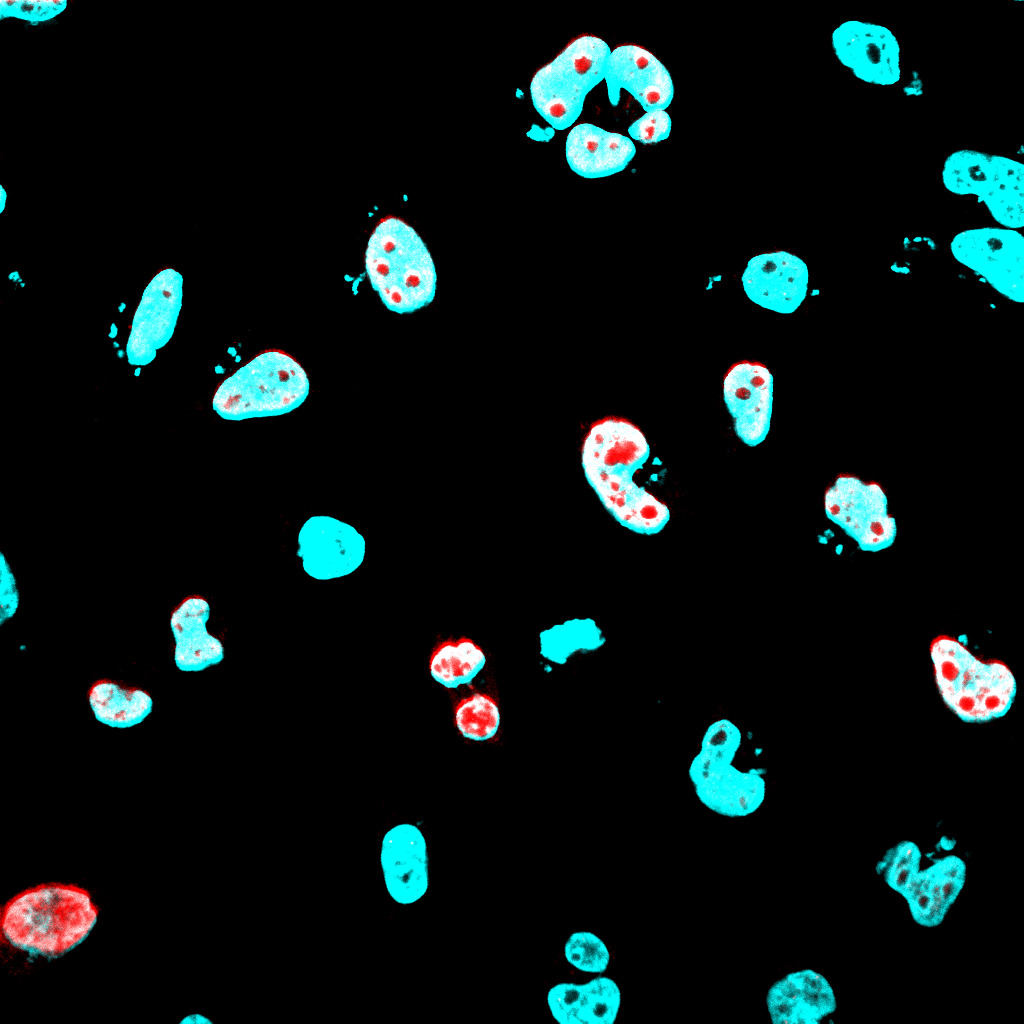

Supplement: Supplementary file 4 — Source data [file 41467_2024_53777_MOESM4_ESM.zip › Source-Data/FigureS10/source-data-Supplementary-Figure-S10A/HA_p110dsRBD3-interface-mutant-delta-deaminase_merge.jpg]

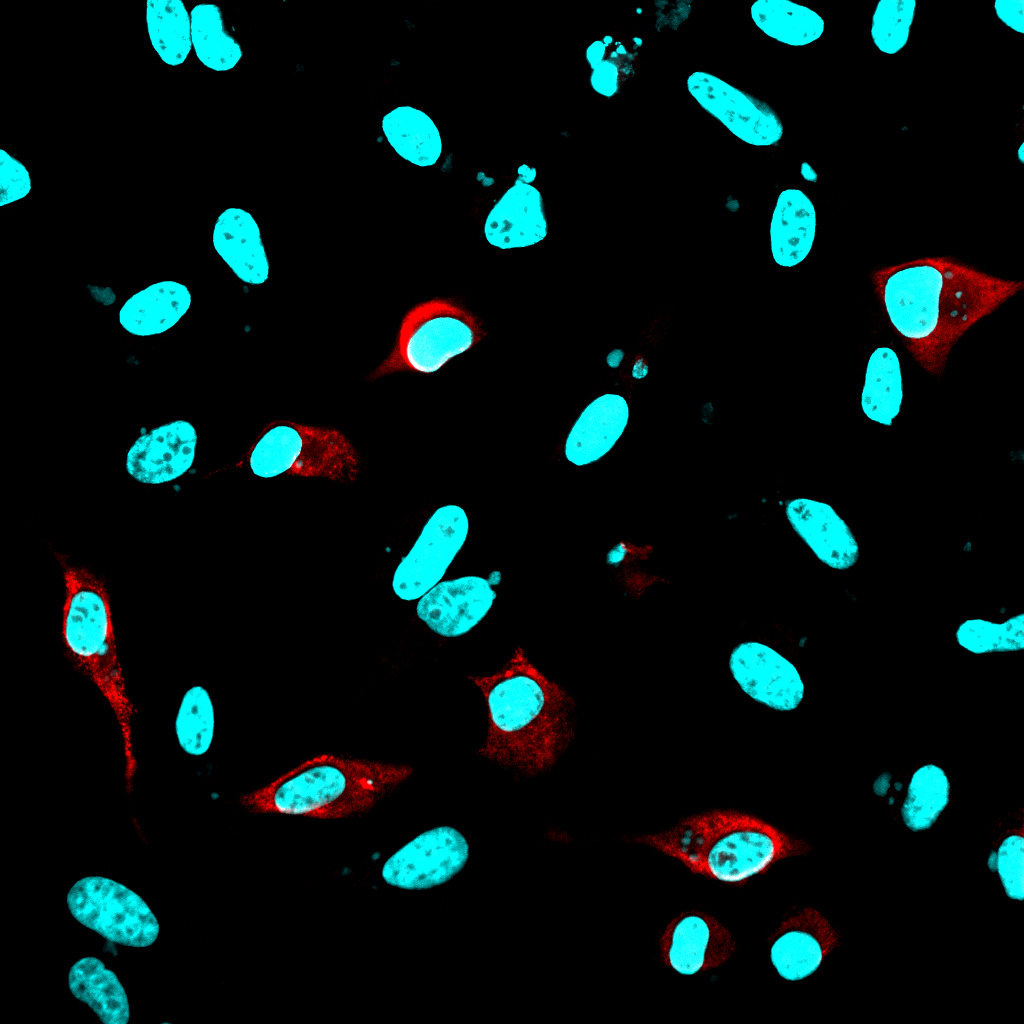

Supplement: Supplementary file 4 — Source data [file 41467_2024_53777_MOESM4_ESM.zip › Source-Data/FigureS10/source-data-Supplementary-Figure-S10A/Flag_p150_merge.jpg]

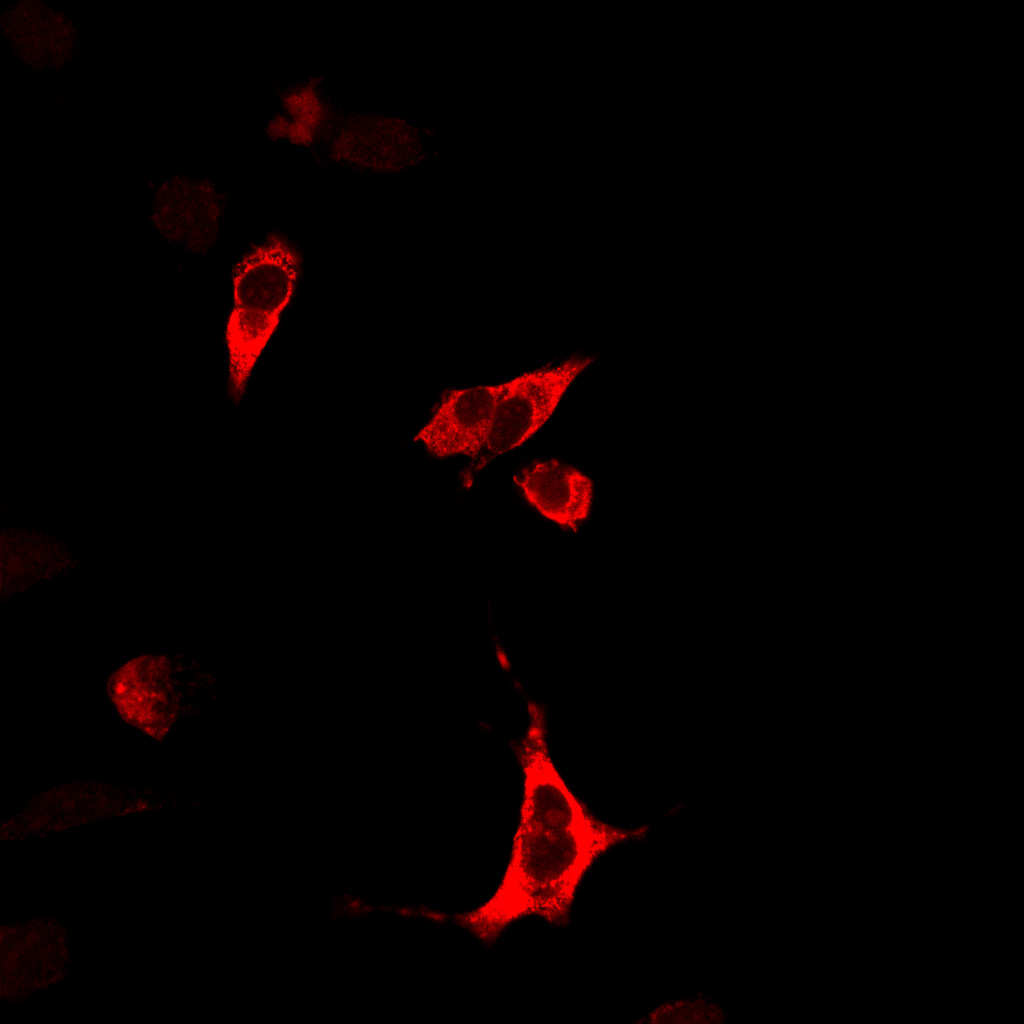

Supplement: Supplementary file 4 — Source data [file 41467_2024_53777_MOESM4_ESM.zip › Source-Data/FigureS10/source-data-Supplementary-Figure-S10A/Flag_p150dsRBD3-interface-mutant_TritC.jpg]

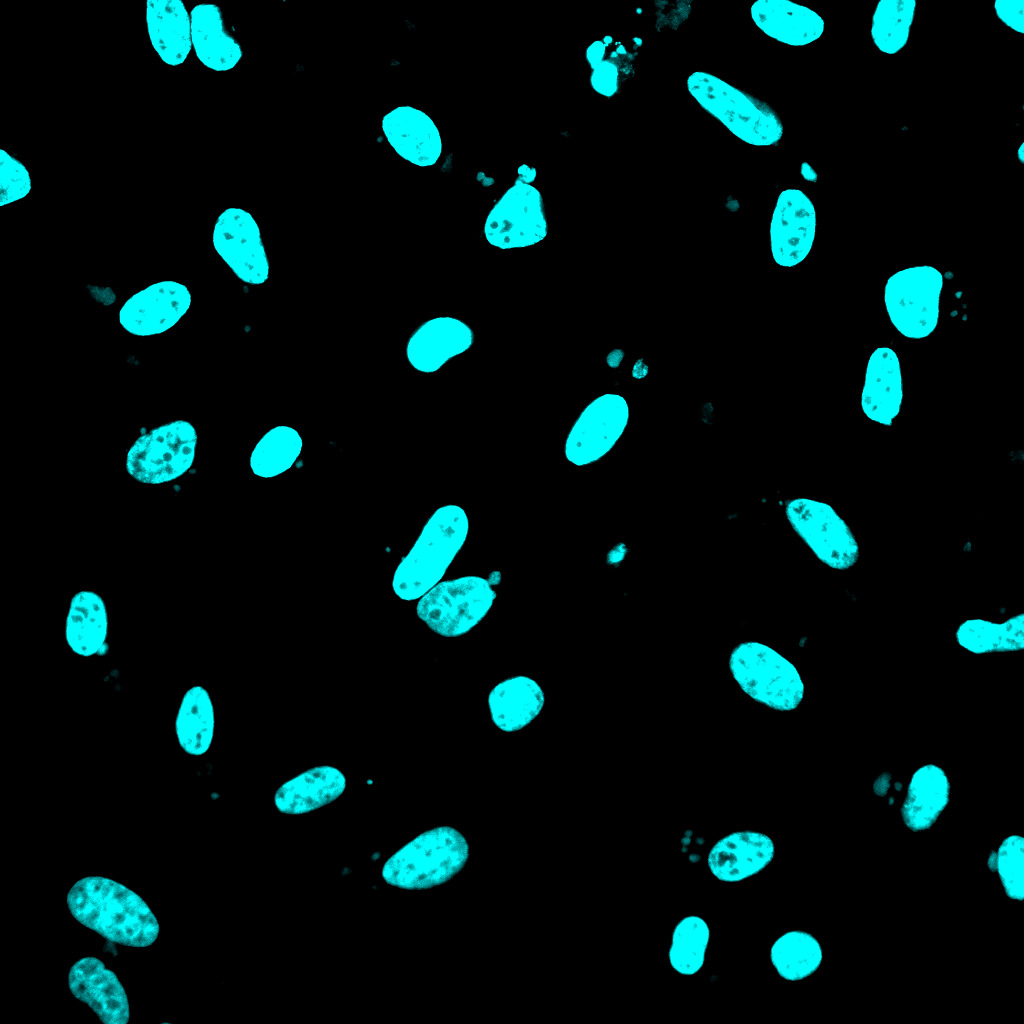

Supplement: Supplementary file 4 — Source data [file 41467_2024_53777_MOESM4_ESM.zip › Source-Data/FigureS10/source-data-Supplementary-Figure-S10A/Flag_p150_DAPI.jpg]

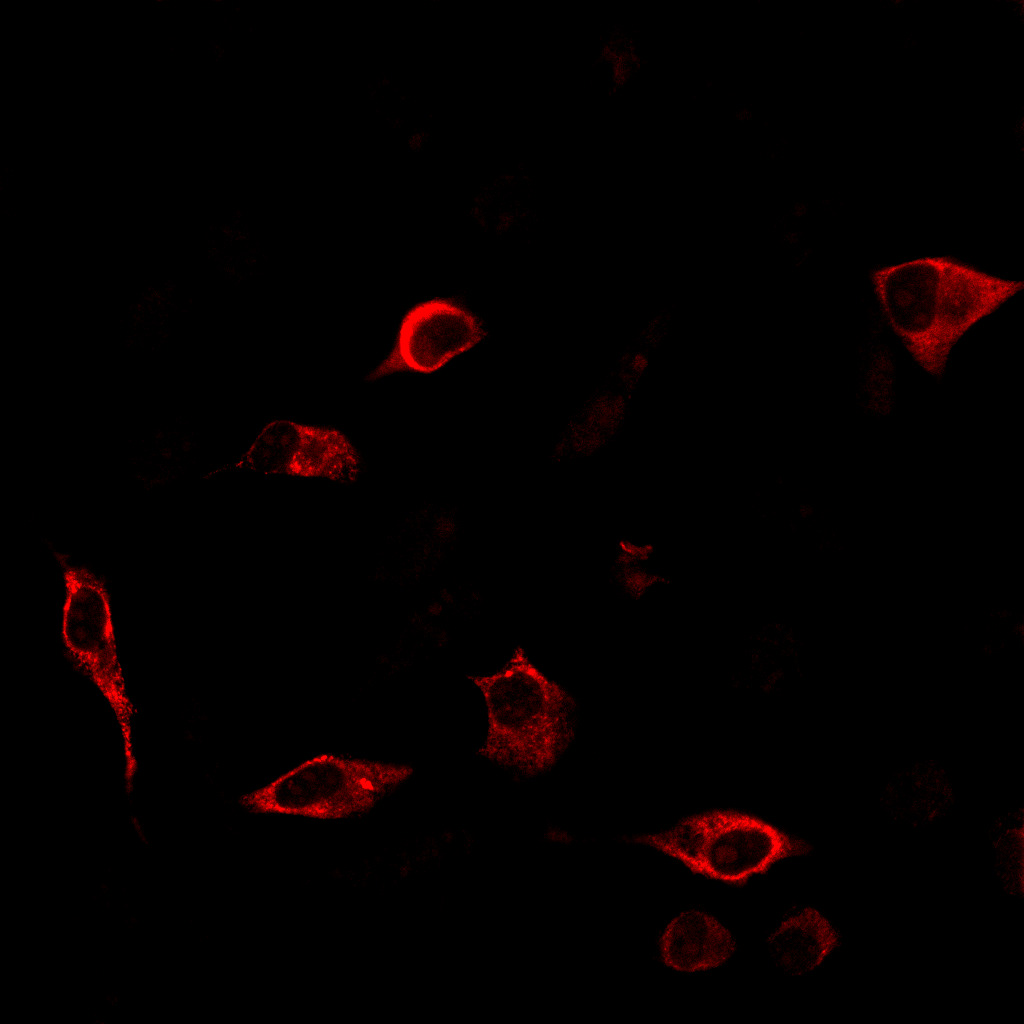

Supplement: Supplementary file 4 — Source data [file 41467_2024_53777_MOESM4_ESM.zip › Source-Data/FigureS10/source-data-Supplementary-Figure-S10A/Flag_p150_TritC.jpg]

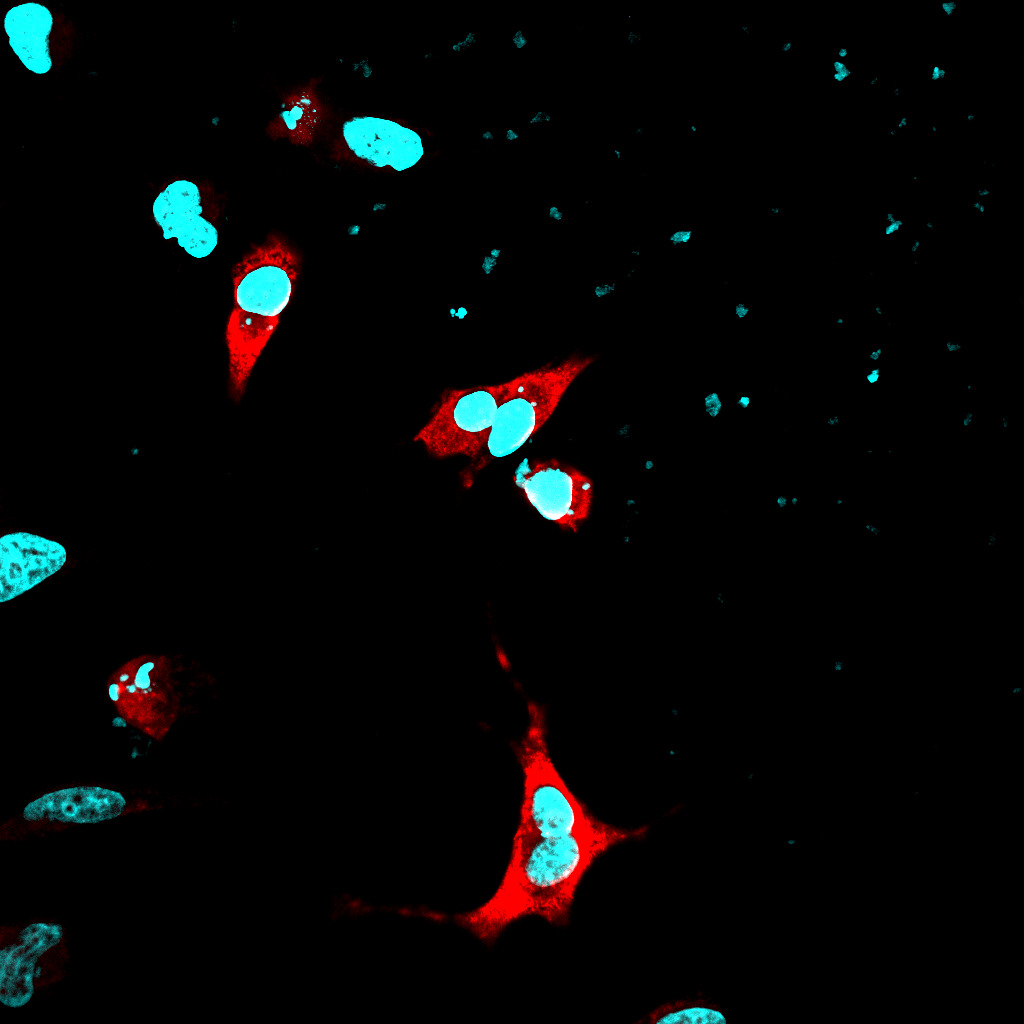

Supplement: Supplementary file 4 — Source data [file 41467_2024_53777_MOESM4_ESM.zip › Source-Data/FigureS10/source-data-Supplementary-Figure-S10A/Flag_p150dsRBD3-interface-mutant_merge.jpg]

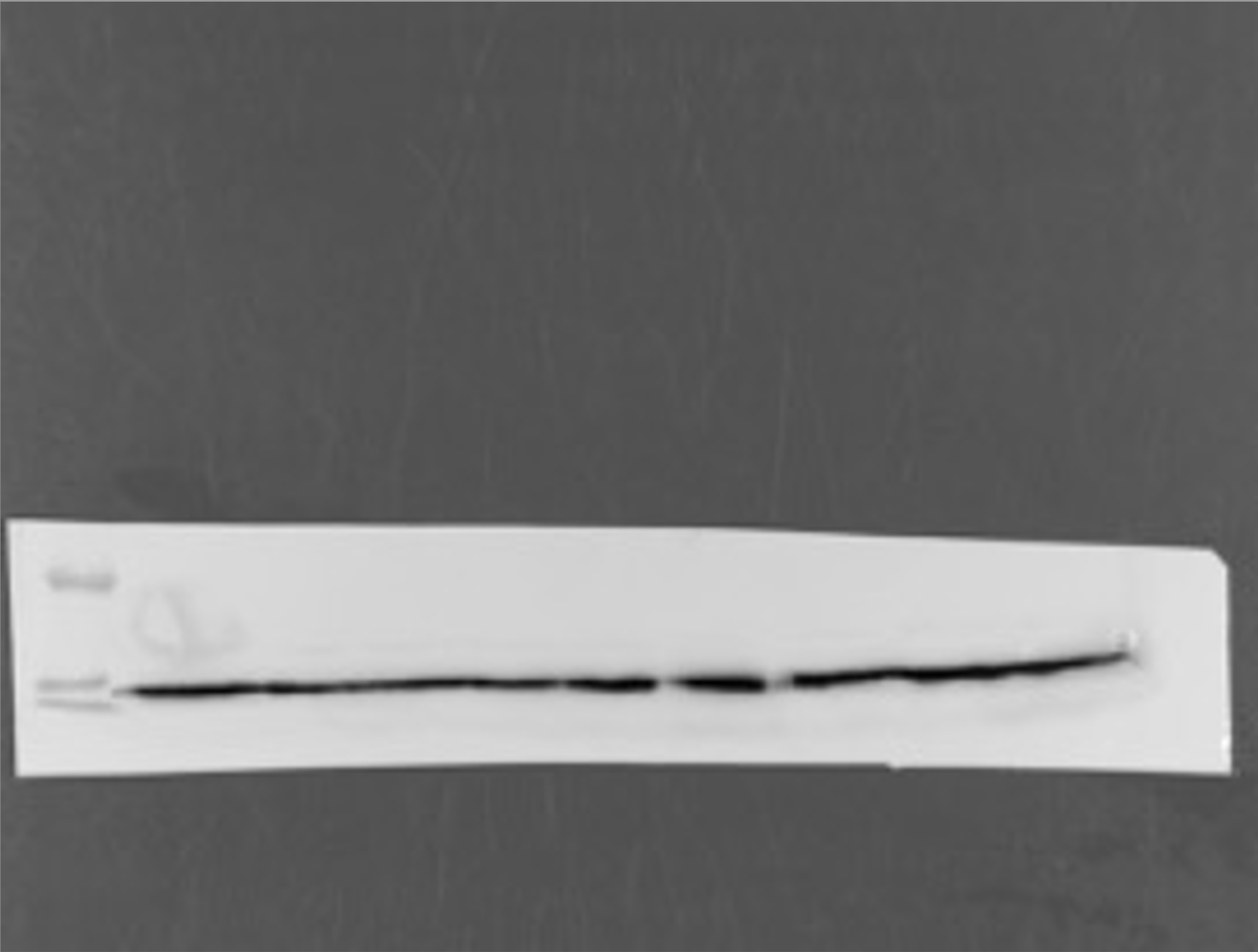

Supplement: Supplementary file 4 — Source data [file 41467_2024_53777_MOESM4_ESM.zip › Source-Data/FigureS10/raw-data-Supplementary-Figure-S10B/FigureS10B_GAPDH.jpg]

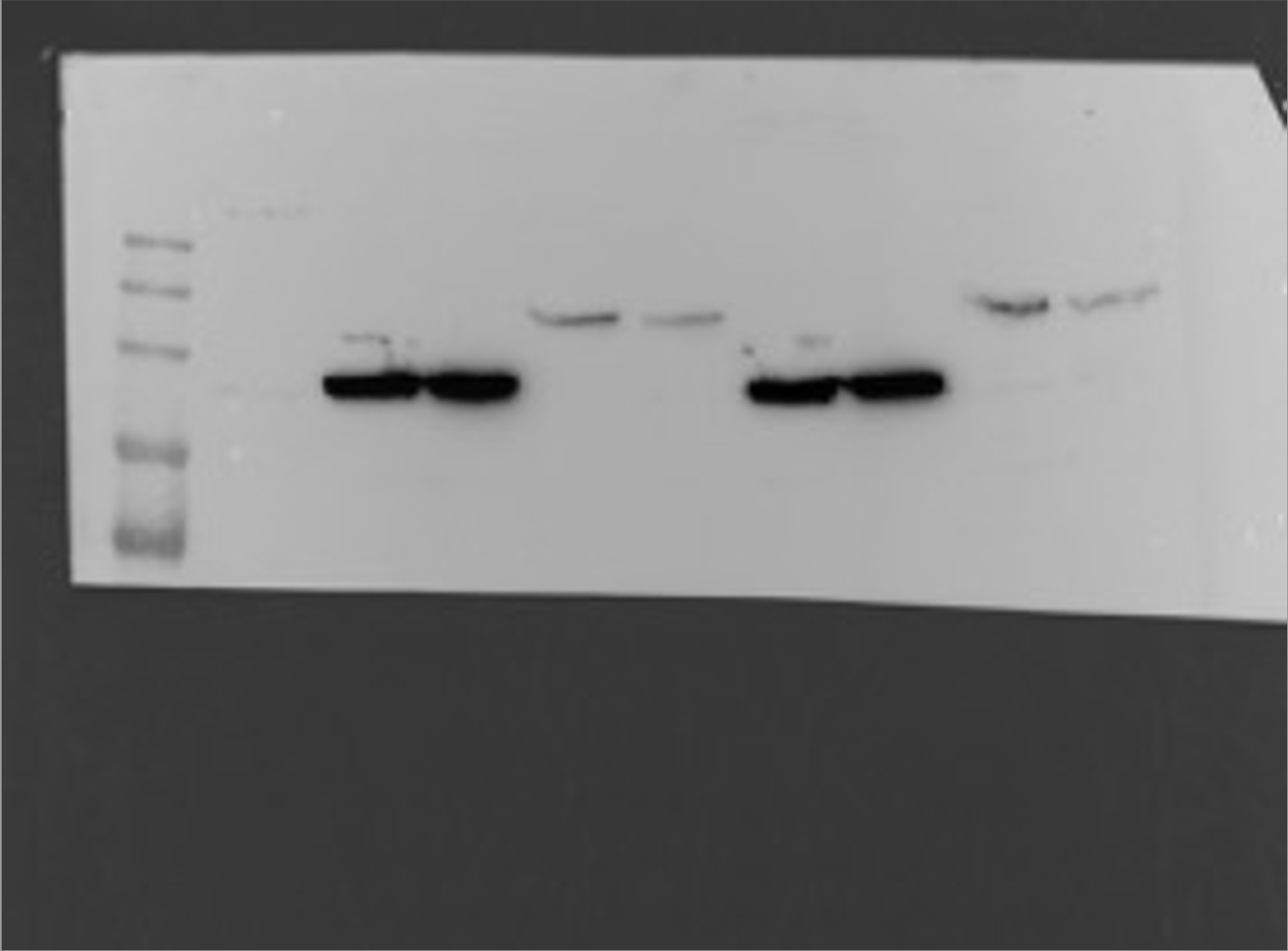

Supplement: Supplementary file 4 — Source data [file 41467_2024_53777_MOESM4_ESM.zip › Source-Data/FigureS10/raw-data-Supplementary-Figure-S10B/FigureS10B_FLAG.jpg]

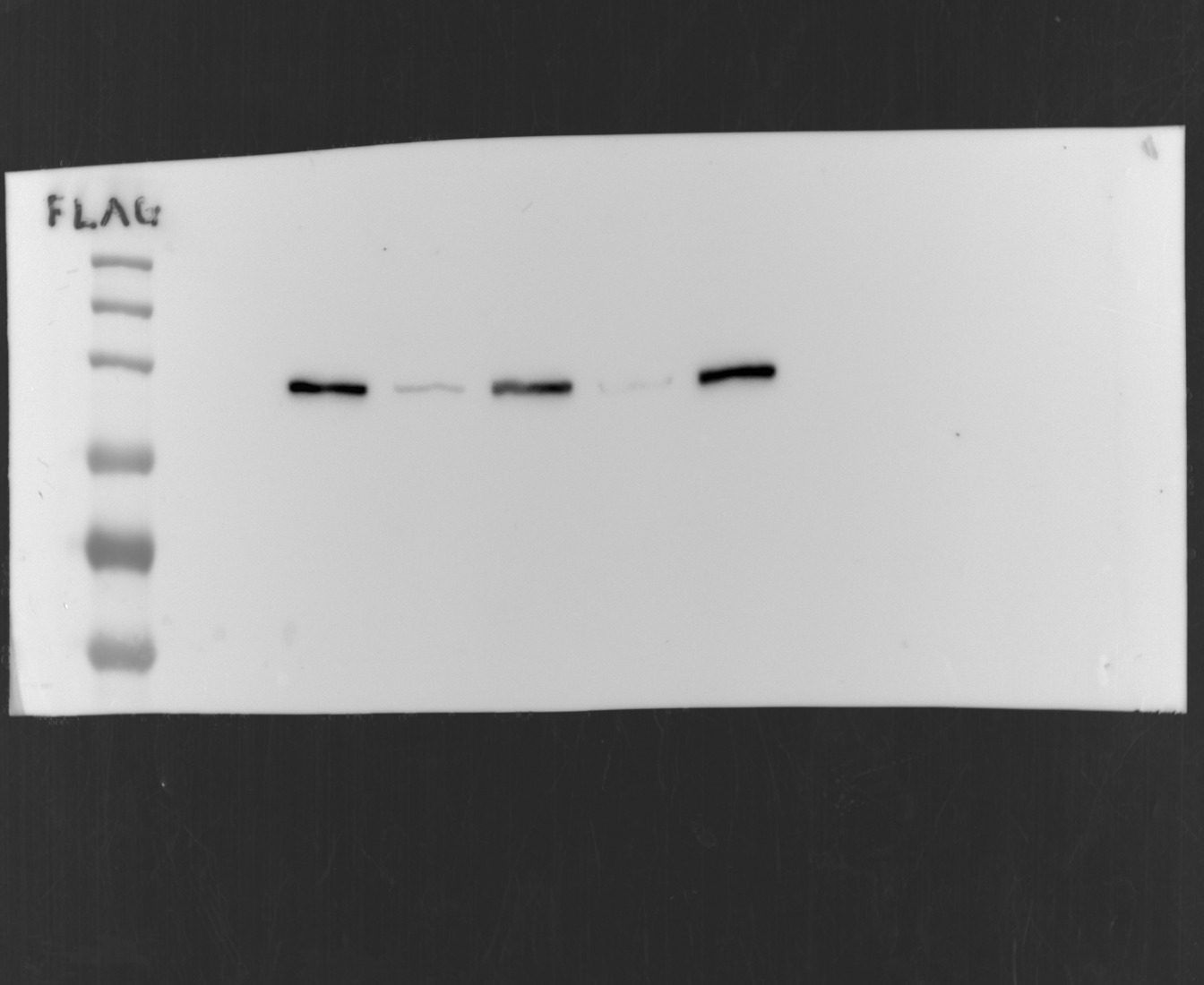

Supplement: Supplementary file 4 — Source data [file 41467_2024_53777_MOESM4_ESM.zip › Source-Data/FigureS11/Raw-data-FigureS11B/Replicate_3_Flag_Merge.jpg]

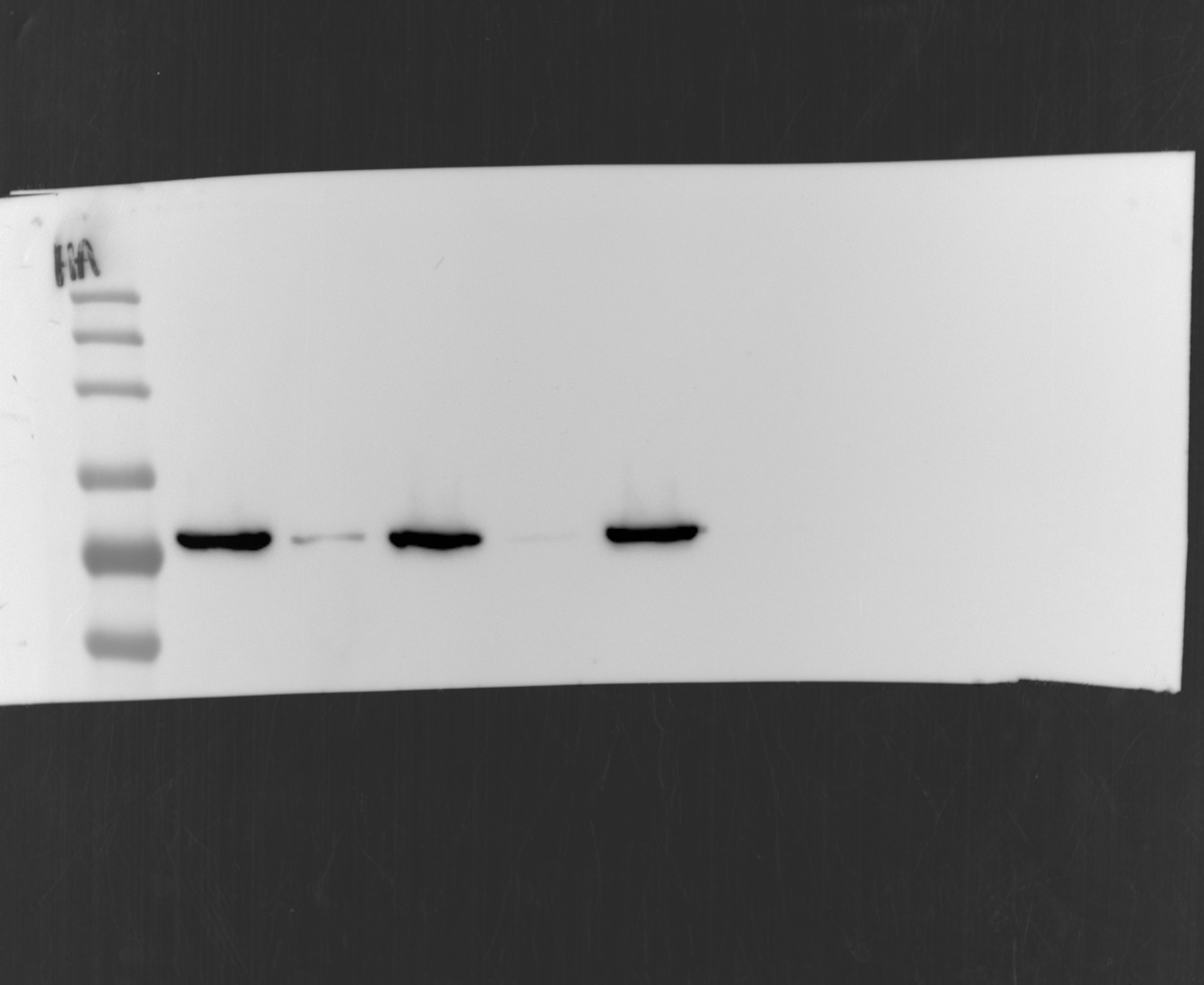

Supplement: Supplementary file 4 — Source data [file 41467_2024_53777_MOESM4_ESM.zip › Source-Data/FigureS11/Raw-data-FigureS11B/Replicate_3_HA-delta-deaminase_Merge.jpg]

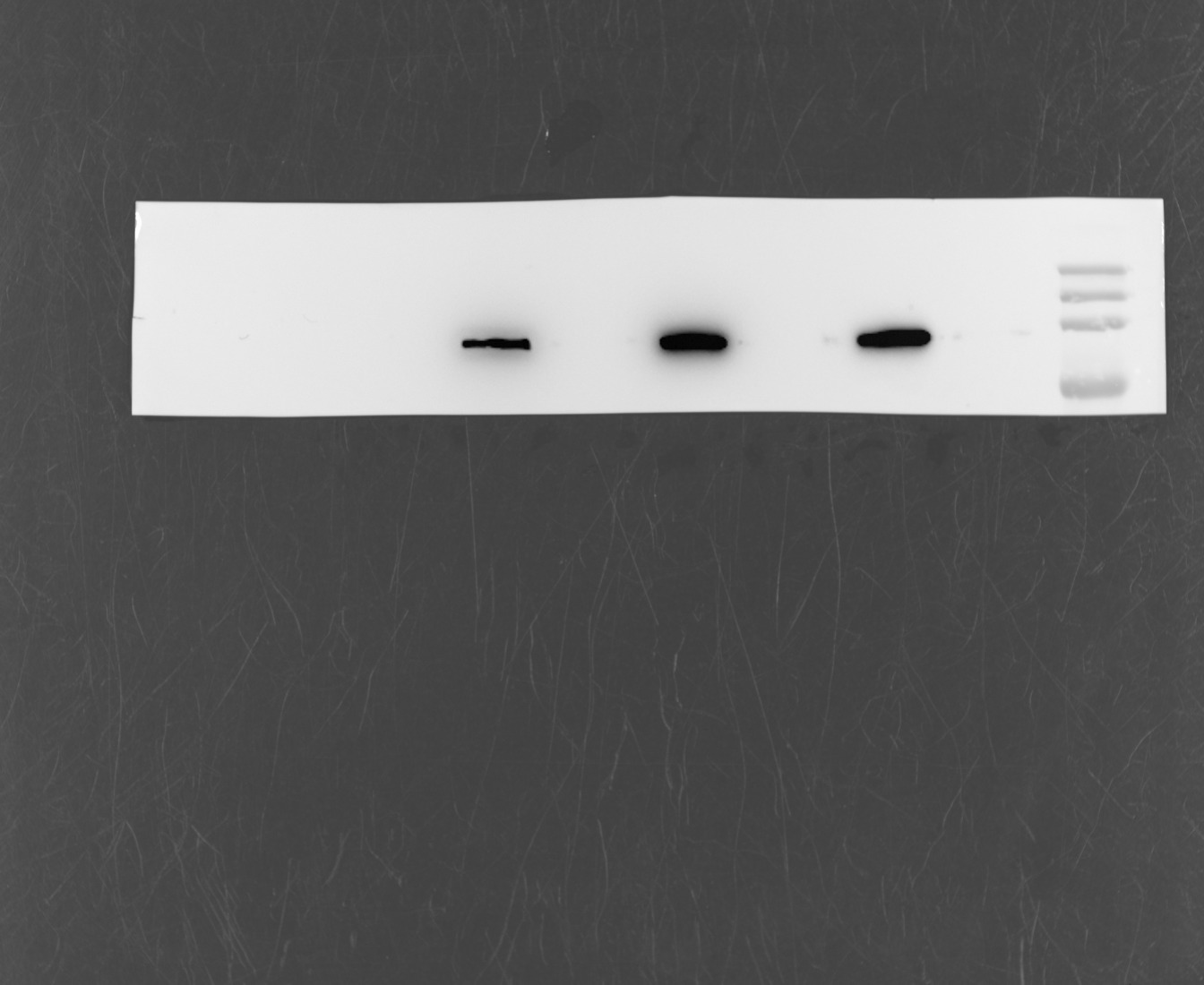

Supplement: Supplementary file 4 — Source data [file 41467_2024_53777_MOESM4_ESM.zip › Source-Data/FigureS11/Raw-data-FigureS11B/Replicate_2_Flag_Merge.jpg]

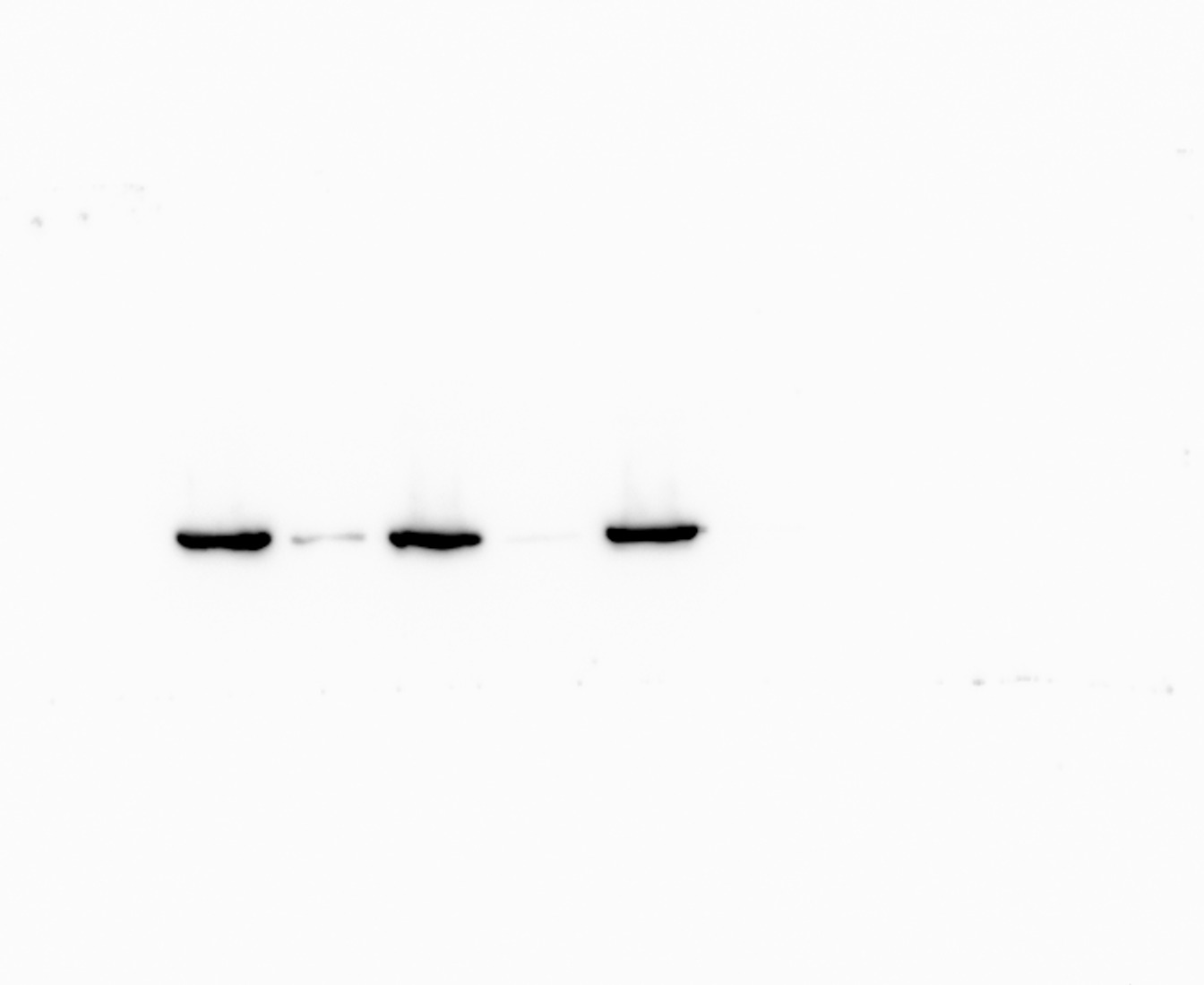

Supplement: Supplementary file 4 — Source data [file 41467_2024_53777_MOESM4_ESM.zip › Source-Data/FigureS11/Raw-data-FigureS11B/Replicate_3_HA-delta-deaminase_Bands.jpg]

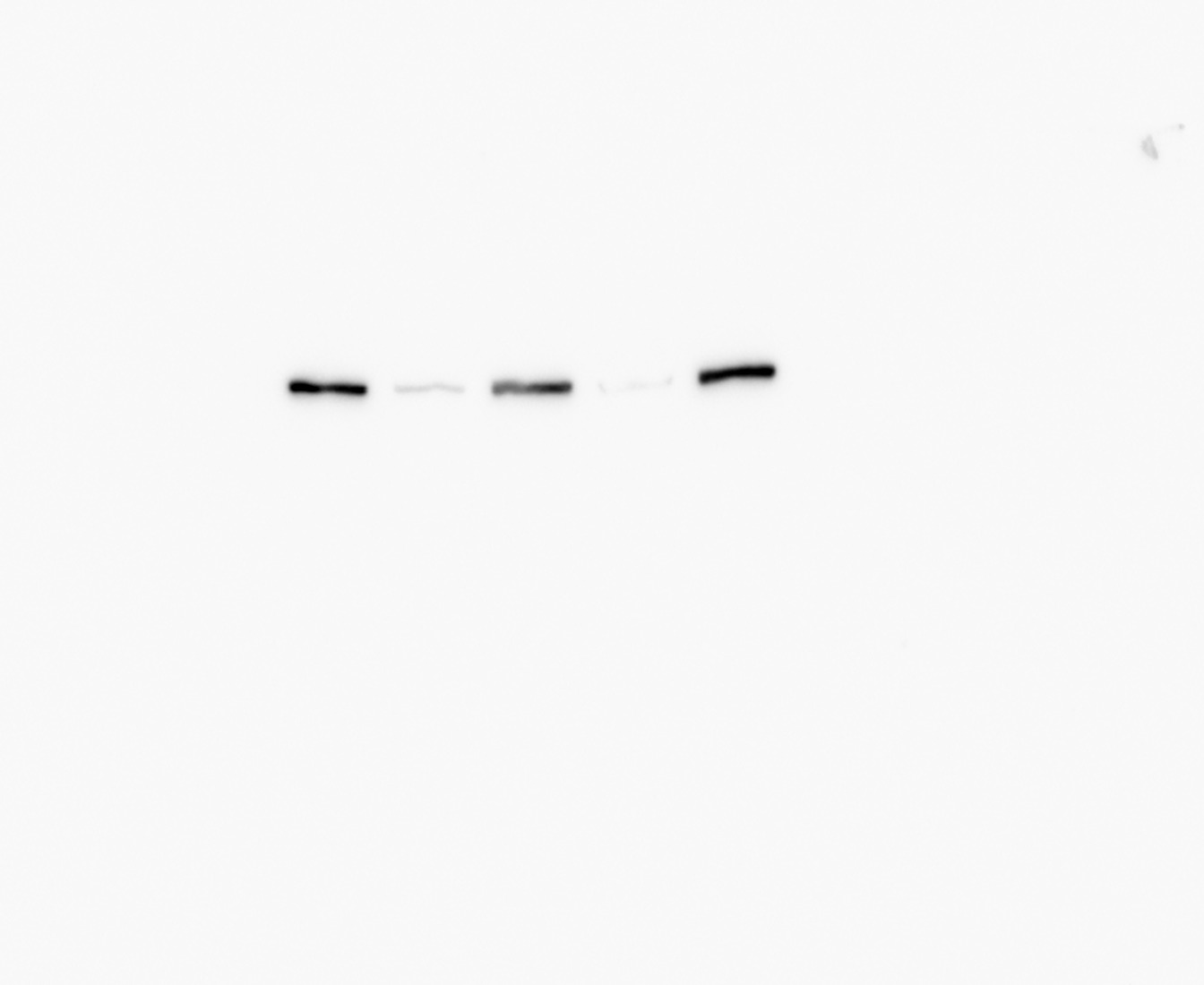

Supplement: Supplementary file 4 — Source data [file 41467_2024_53777_MOESM4_ESM.zip › Source-Data/FigureS11/Raw-data-FigureS11B/Replicate_3_Flag_Bands.jpg]

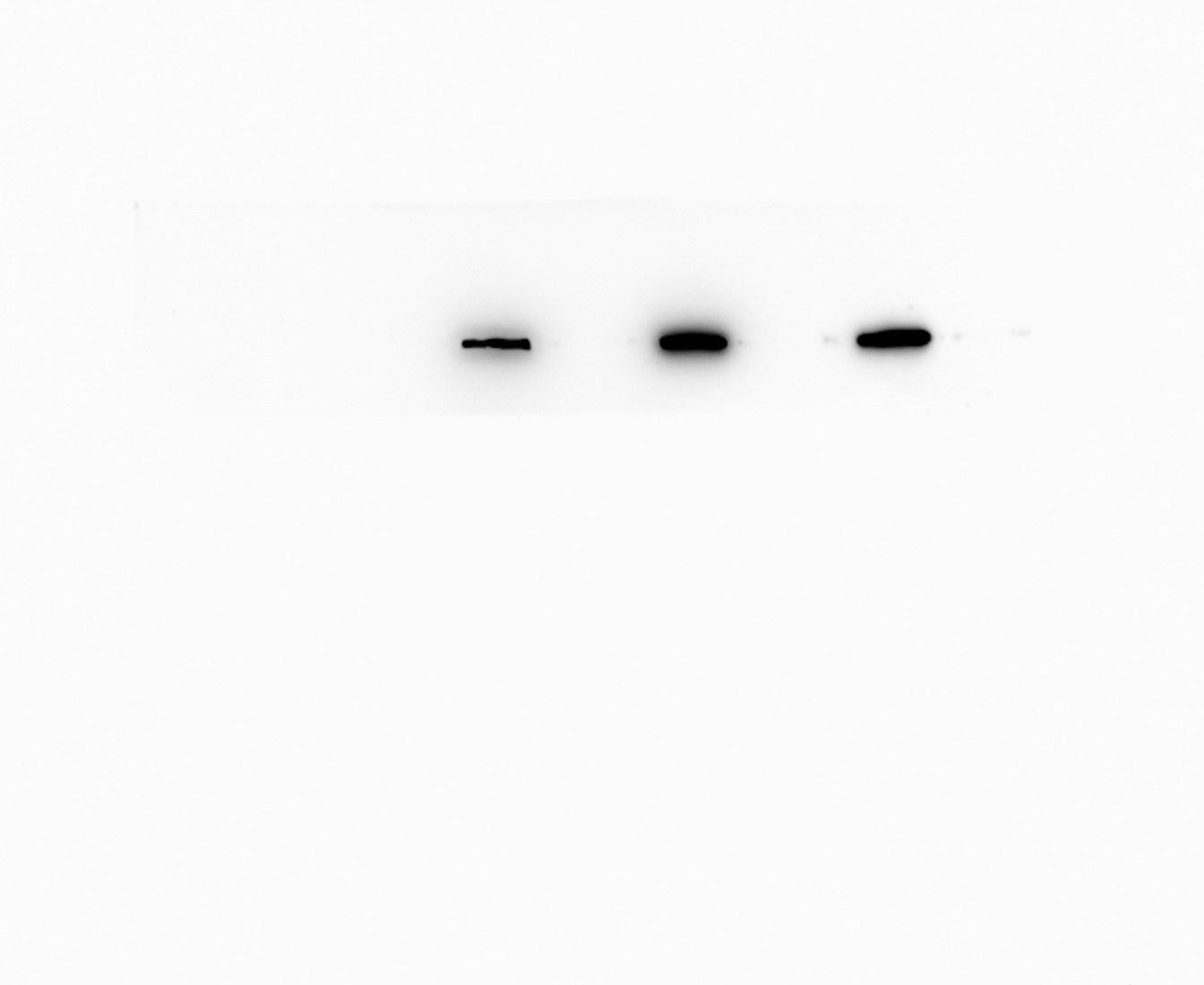

Supplement: Supplementary file 4 — Source data [file 41467_2024_53777_MOESM4_ESM.zip › Source-Data/FigureS11/Raw-data-FigureS11B/Replicate_2_Flag_Bands.jpg]

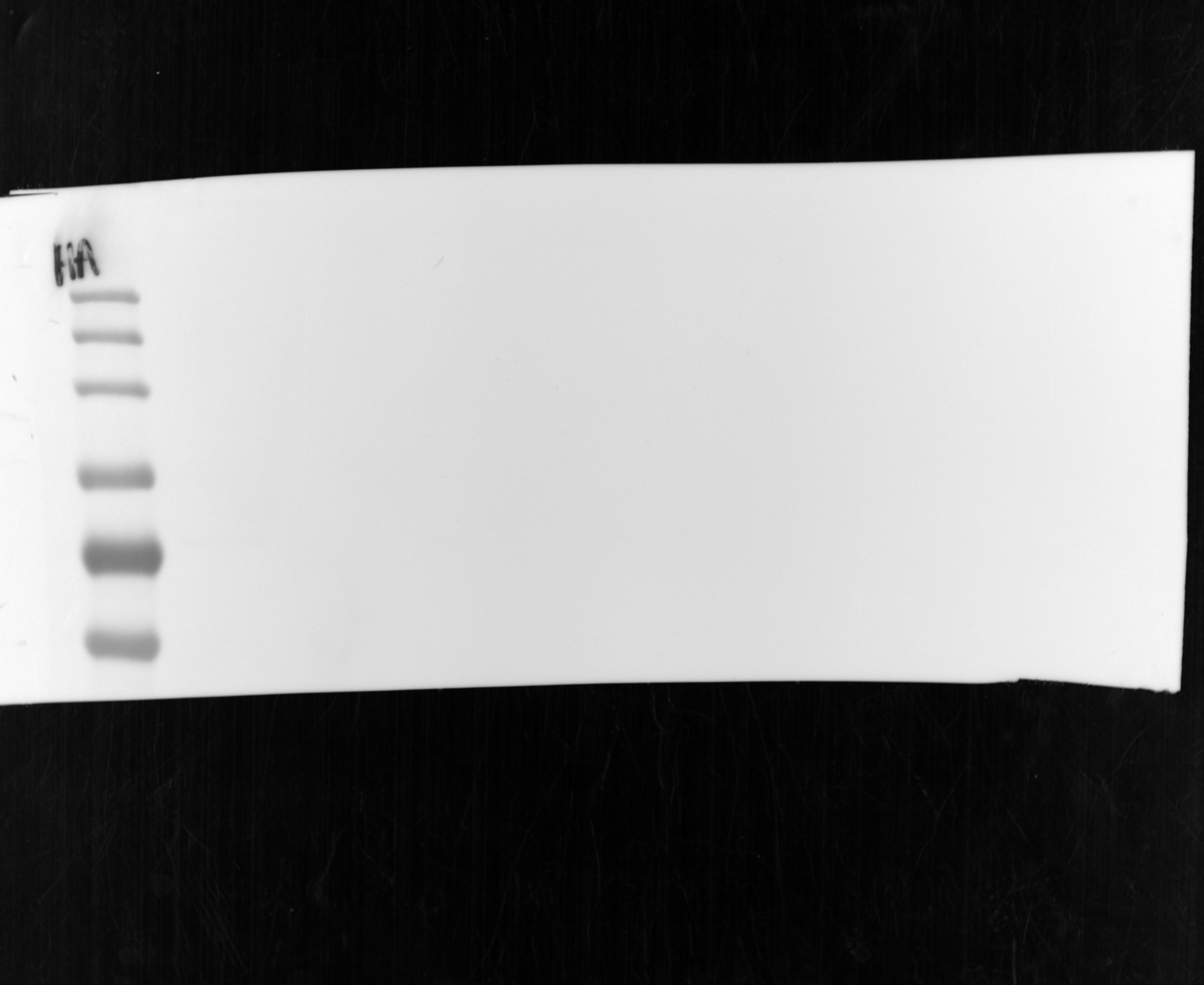

Supplement: Supplementary file 4 — Source data [file 41467_2024_53777_MOESM4_ESM.zip › Source-Data/FigureS11/Raw-data-FigureS11B/Replicate_3_HA-deltadeaminase_Marker.jpg]

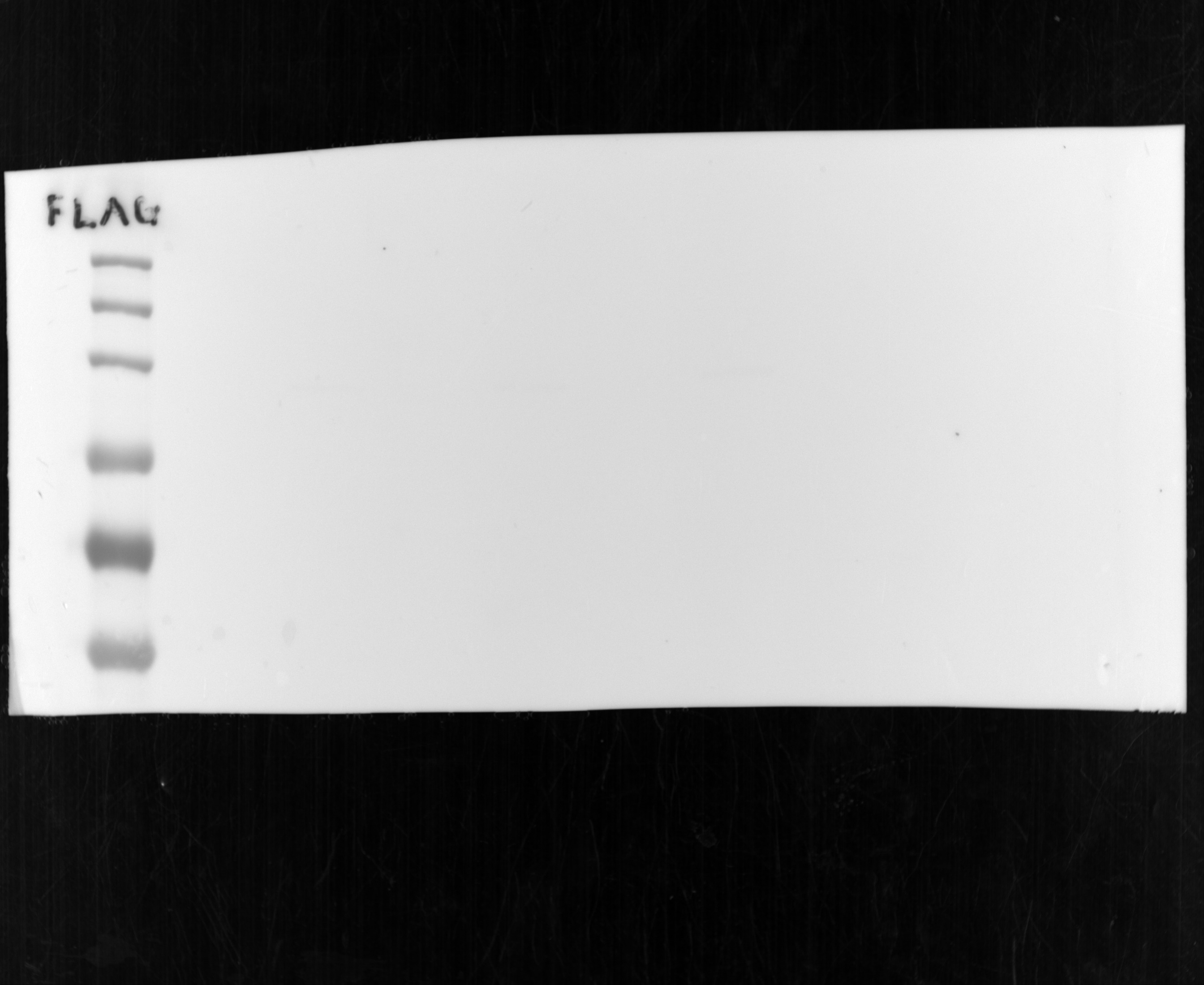

Supplement: Supplementary file 4 — Source data [file 41467_2024_53777_MOESM4_ESM.zip › Source-Data/FigureS11/Raw-data-FigureS11B/Replicate_3_Flag_Marker.jpg]

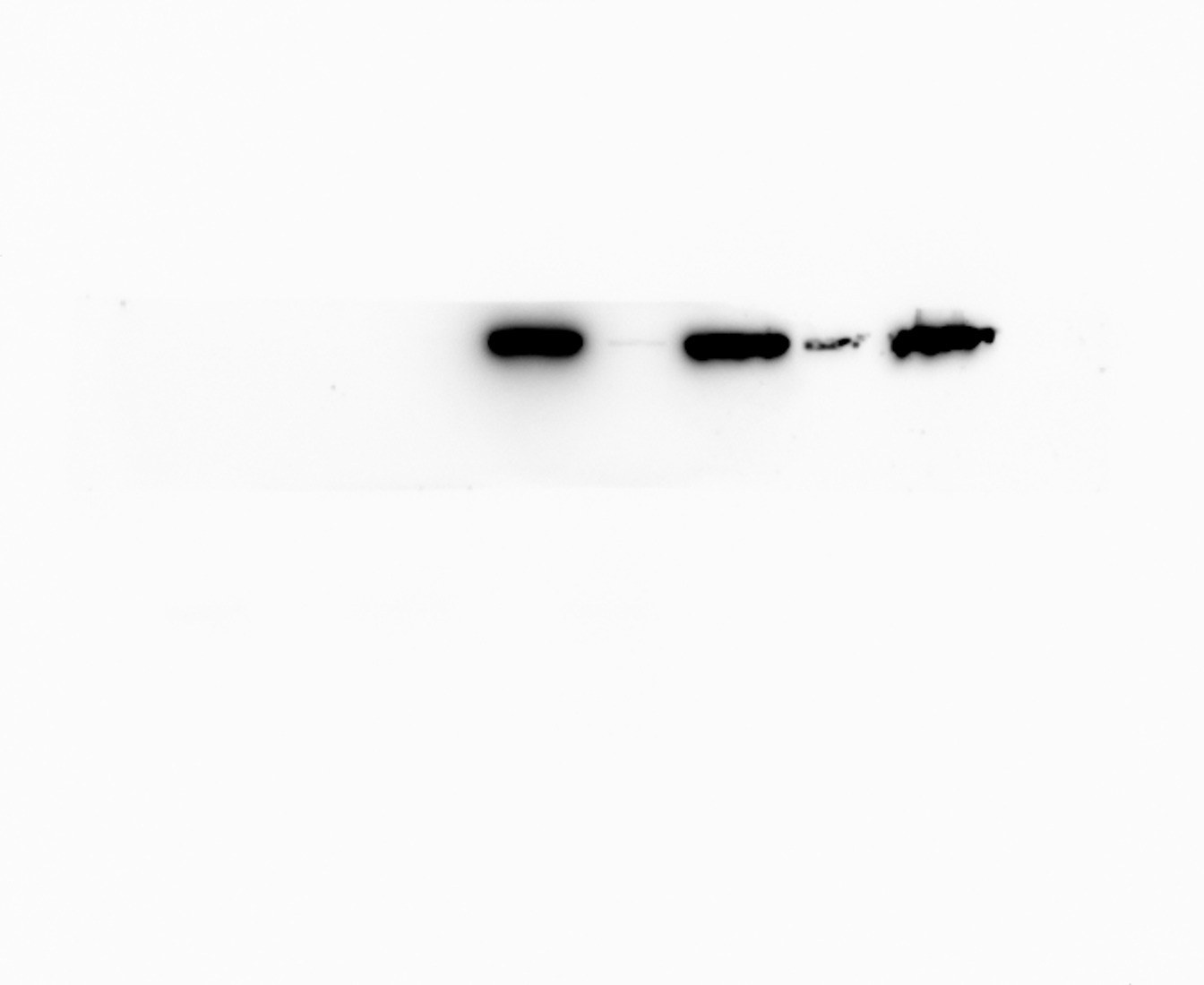

Supplement: Supplementary file 4 — Source data [file 41467_2024_53777_MOESM4_ESM.zip › Source-Data/FigureS11/Raw-data-FigureS11B/Replicate_2_HA-delta-deaminase_Bands.jpg]

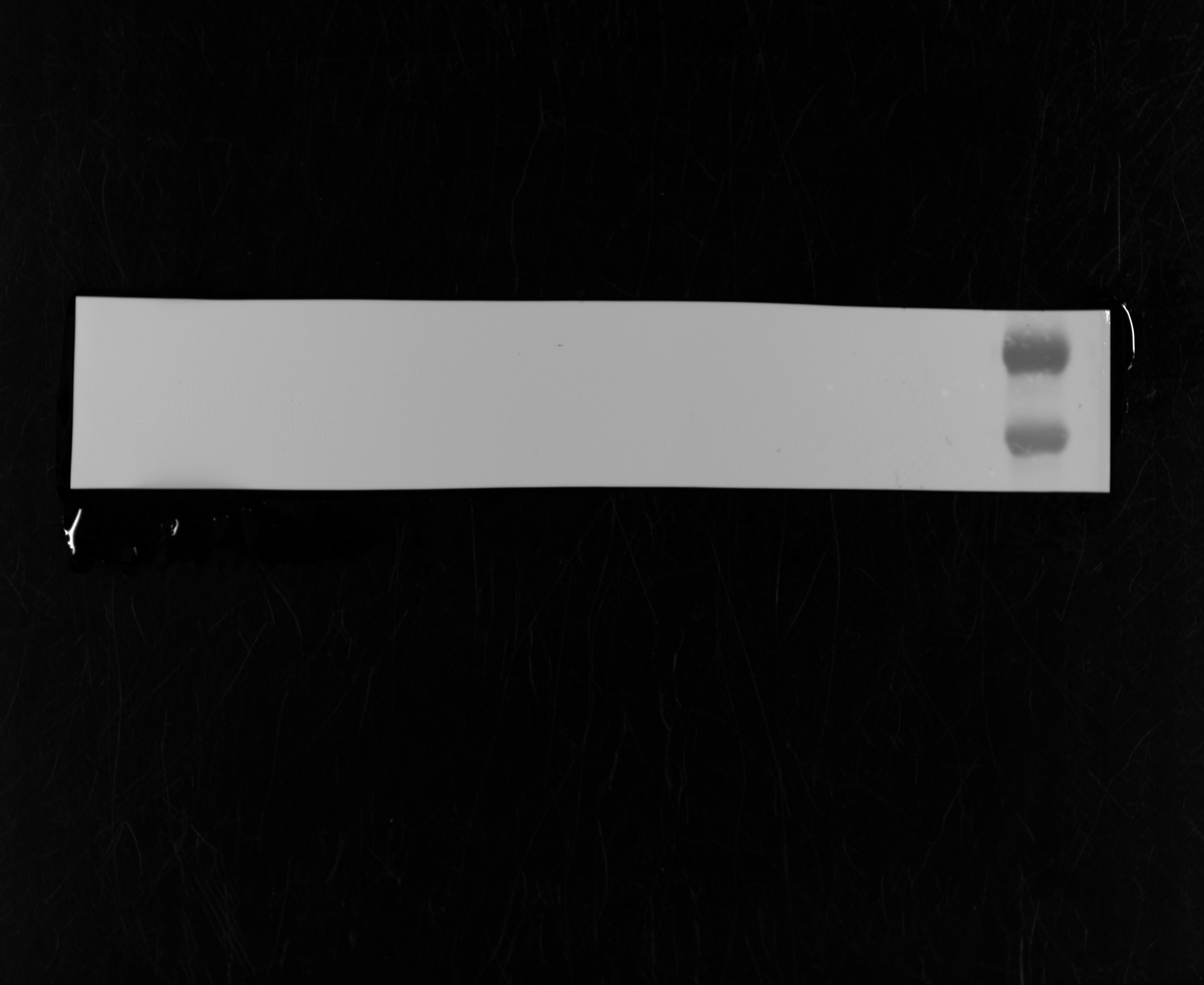

Supplement: Supplementary file 4 — Source data [file 41467_2024_53777_MOESM4_ESM.zip › Source-Data/FigureS11/Raw-data-FigureS11B/Replicate_2_HA-delta-deaminase_Marker.jpg]

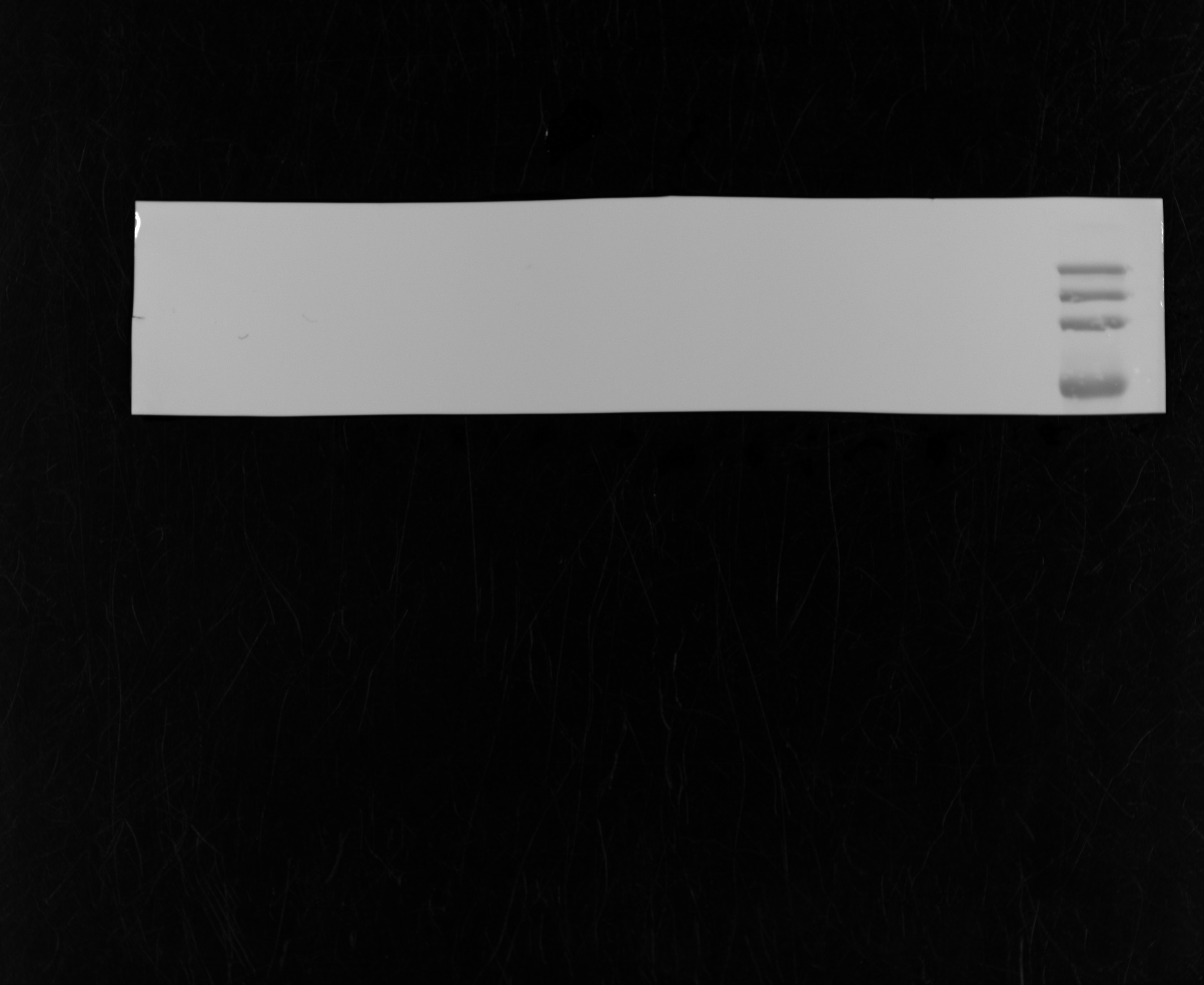

Supplement: Supplementary file 4 — Source data [file 41467_2024_53777_MOESM4_ESM.zip › Source-Data/FigureS11/Raw-data-FigureS11B/Replicate_2_Flag_Marker.jpg]

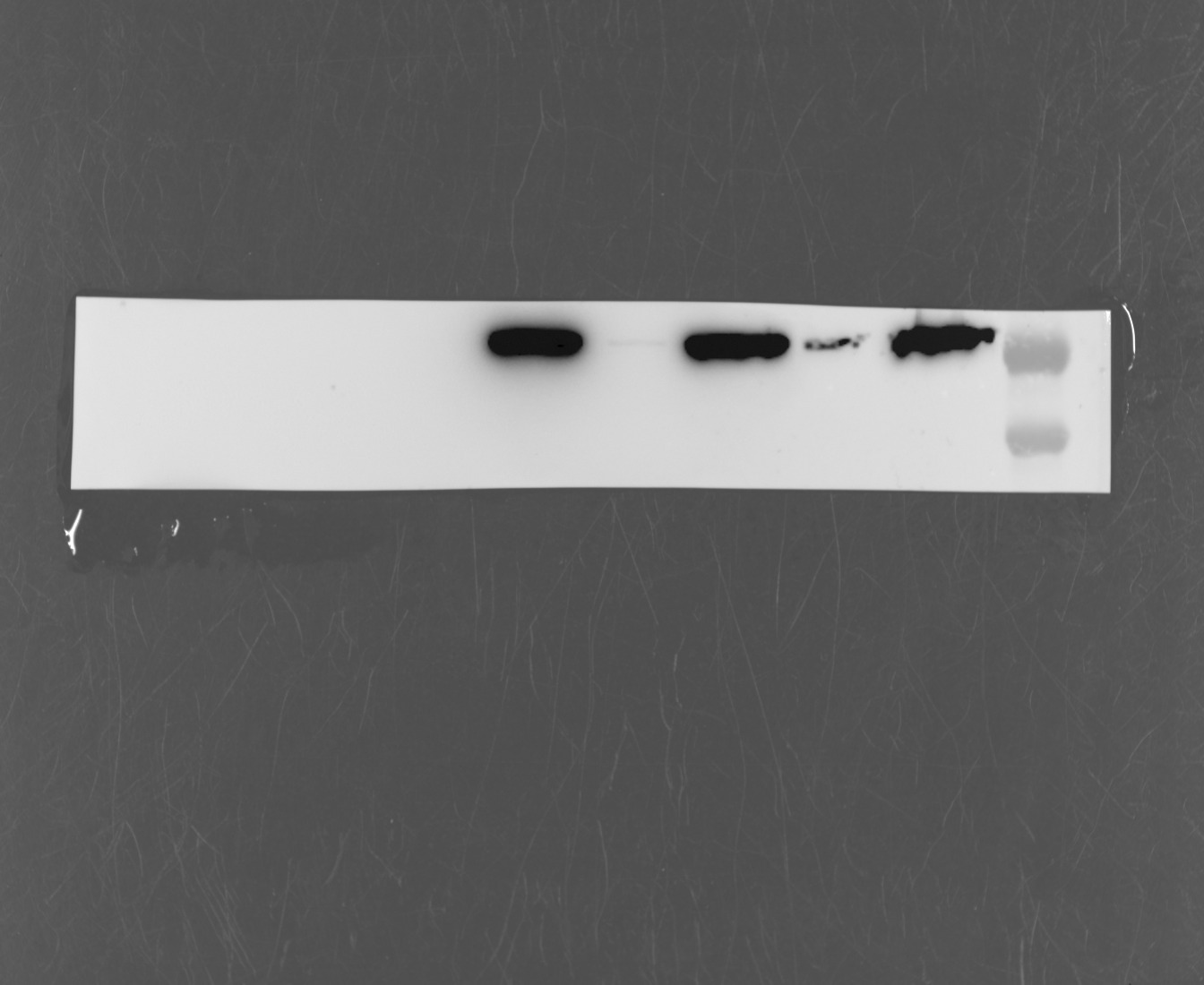

Supplement: Supplementary file 4 — Source data [file 41467_2024_53777_MOESM4_ESM.zip › Source-Data/FigureS11/Raw-data-FigureS11B/Replicate_2_HA-delta-deaminase_Merge.jpg]

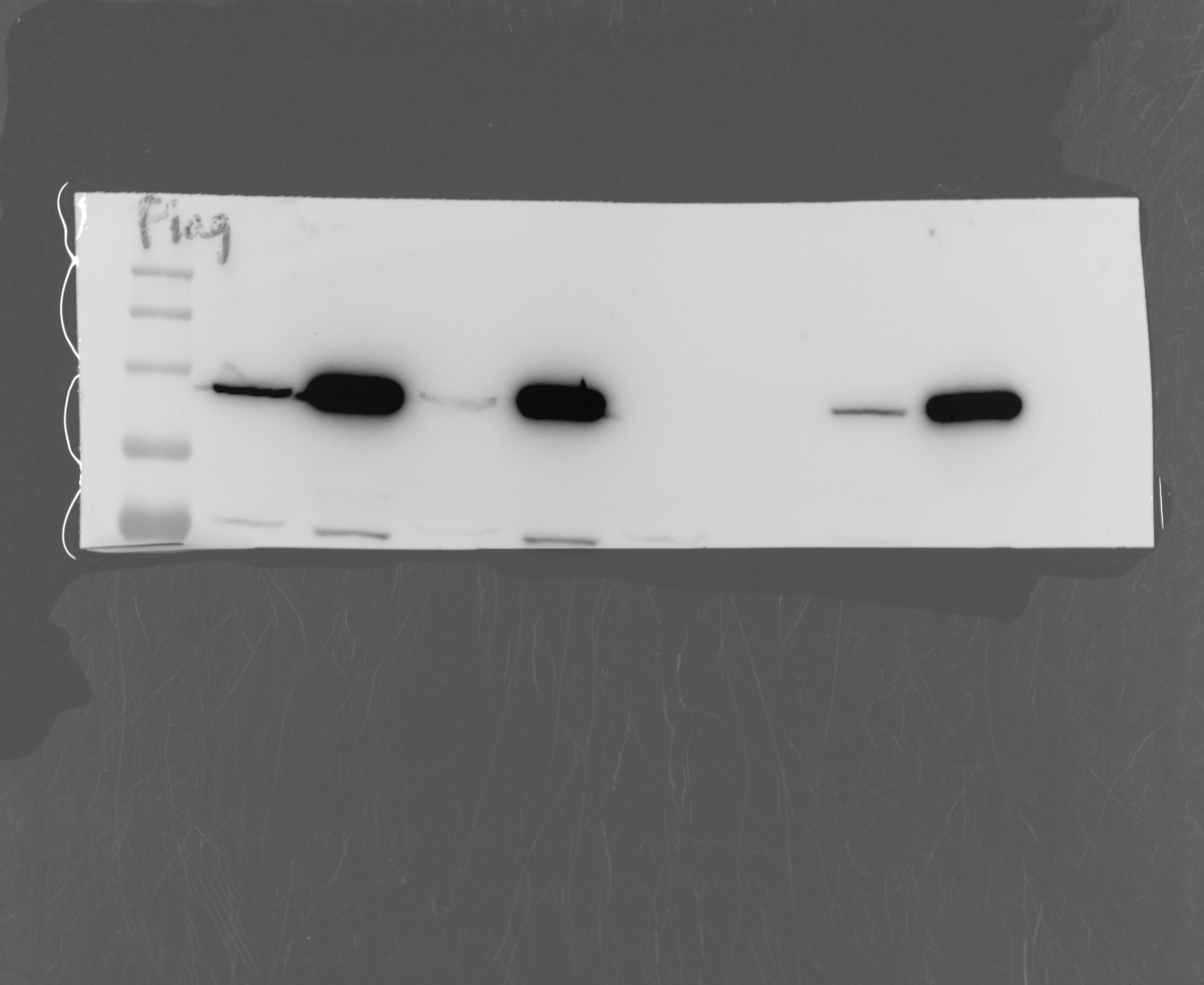

Supplement: Supplementary file 4 — Source data [file 41467_2024_53777_MOESM4_ESM.zip › Source-Data/FigureS11/Raw-data-FigureS11A/Replicate_3_Flag_Merge.jpg]

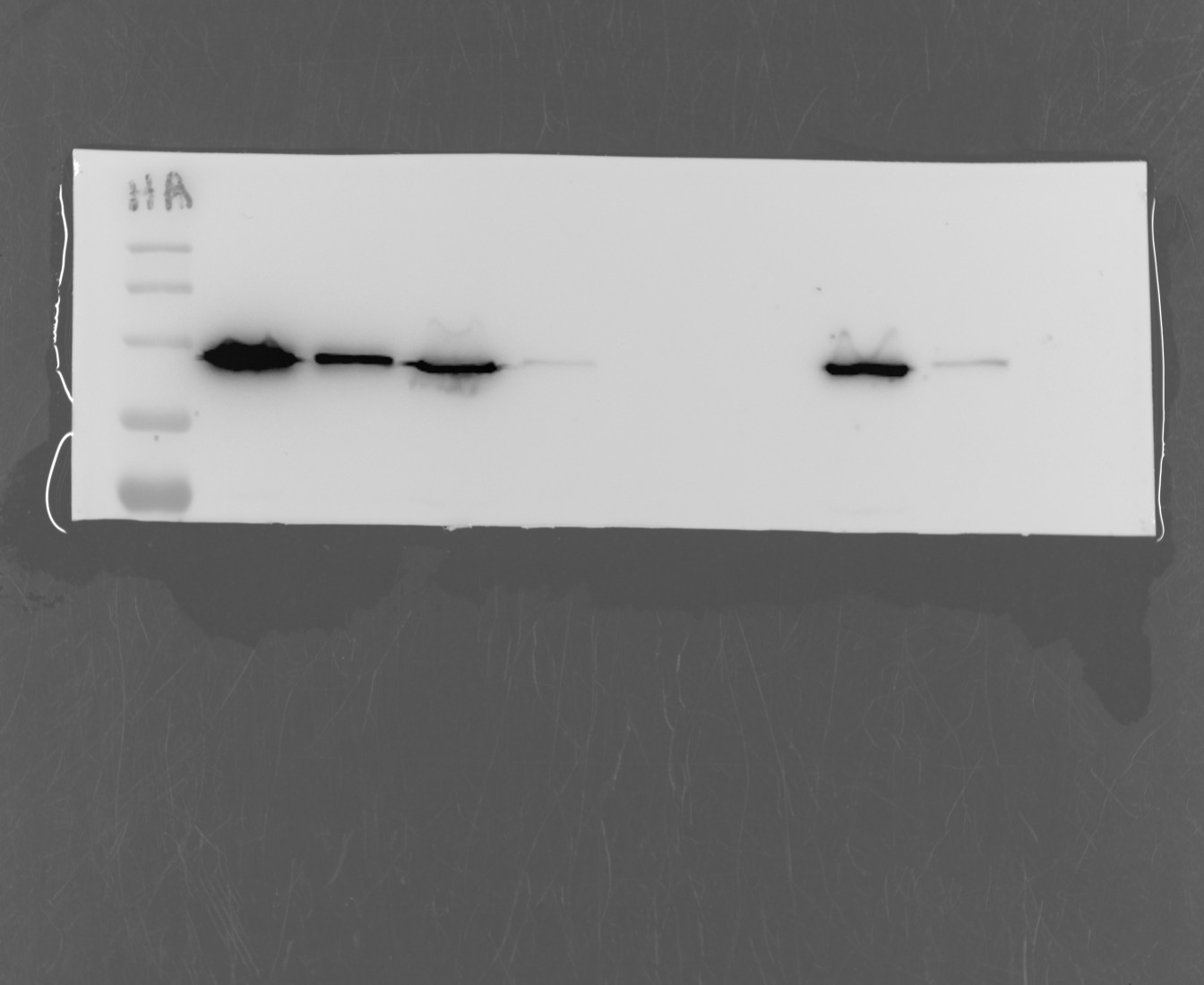

Supplement: Supplementary file 4 — Source data [file 41467_2024_53777_MOESM4_ESM.zip › Source-Data/FigureS11/Raw-data-FigureS11A/Replicate_3_HA_Merge.jpg]

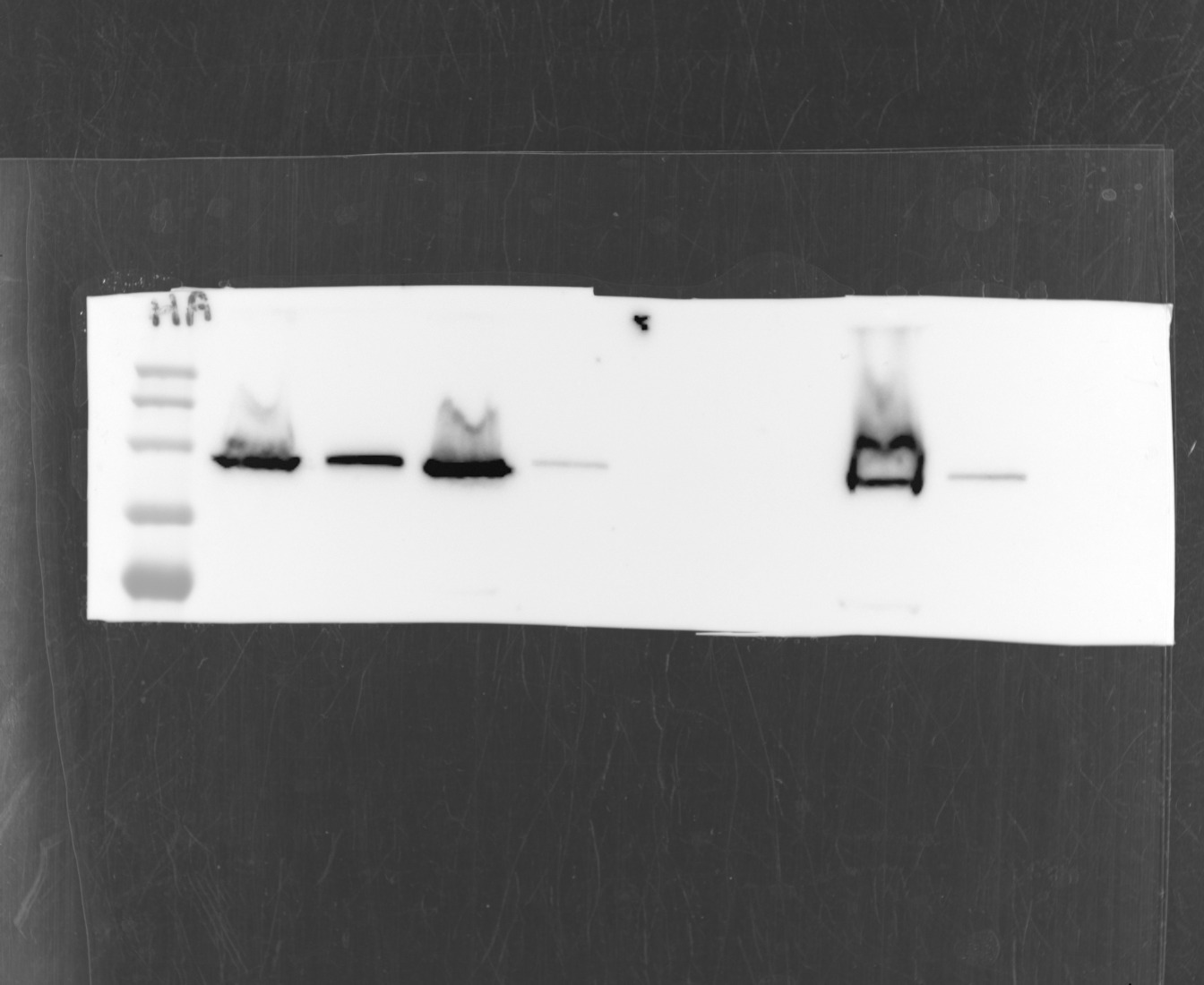

Supplement: Supplementary file 4 — Source data [file 41467_2024_53777_MOESM4_ESM.zip › Source-Data/FigureS11/Raw-data-FigureS11A/Replicate_2_HA_Merge.jpg]

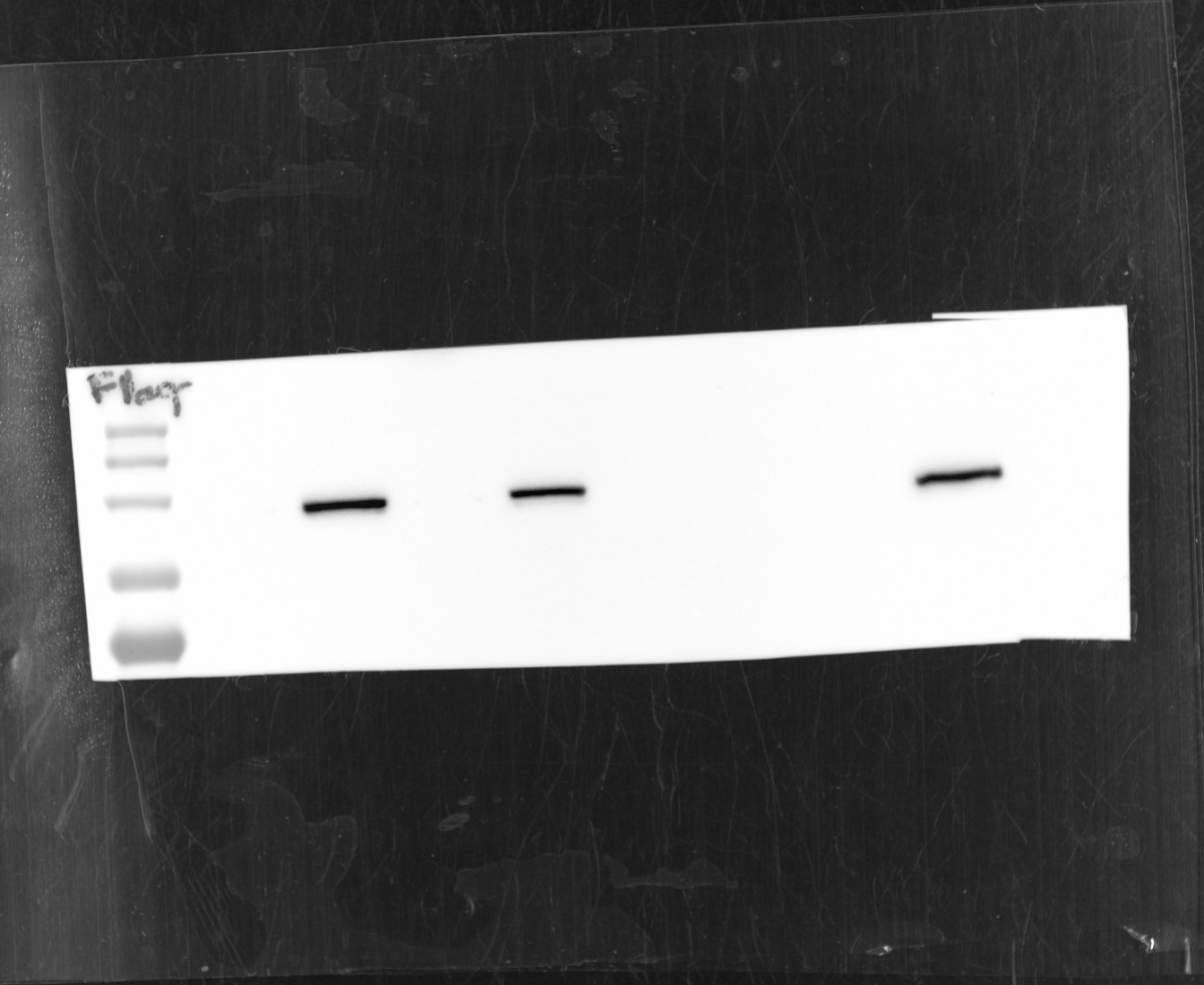

Supplement: Supplementary file 4 — Source data [file 41467_2024_53777_MOESM4_ESM.zip › Source-Data/FigureS11/Raw-data-FigureS11A/Replicate_2_Flag_Merge.jpg]

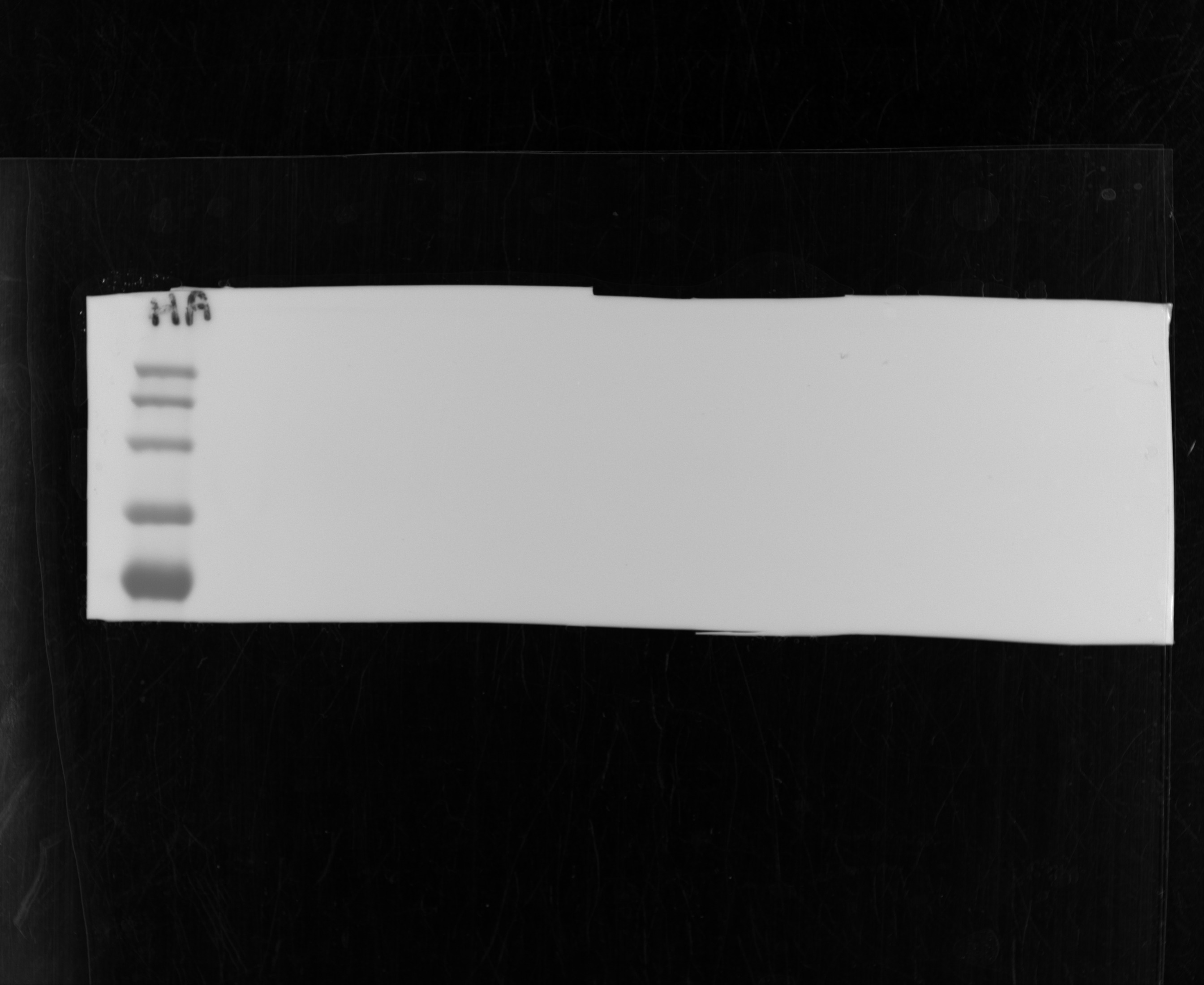

Supplement: Supplementary file 4 — Source data [file 41467_2024_53777_MOESM4_ESM.zip › Source-Data/FigureS11/Raw-data-FigureS11A/Replicate_2_HA_Marker.jpg]

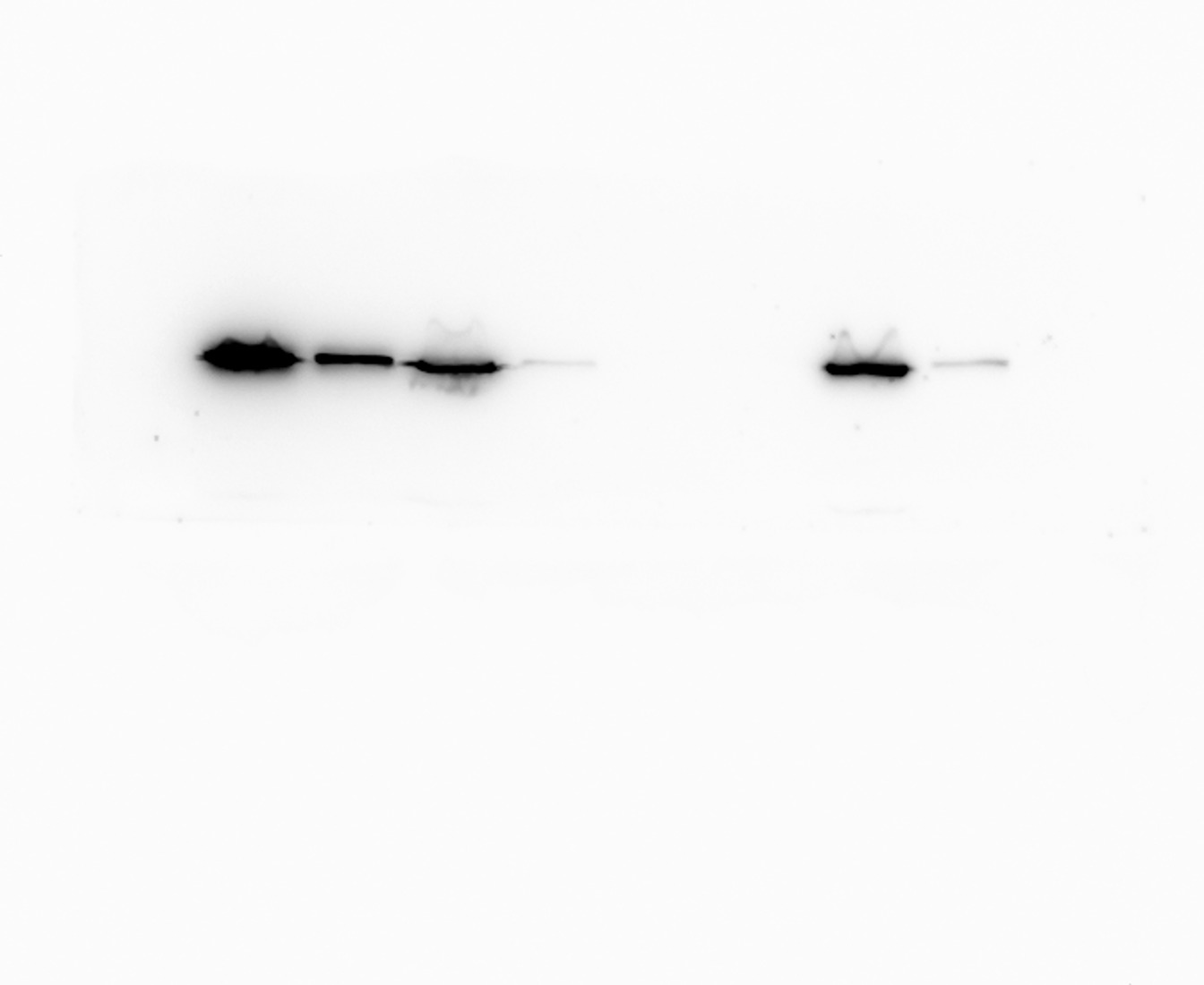

Supplement: Supplementary file 4 — Source data [file 41467_2024_53777_MOESM4_ESM.zip › Source-Data/FigureS11/Raw-data-FigureS11A/Replicate_3_HA_Bands.jpg]

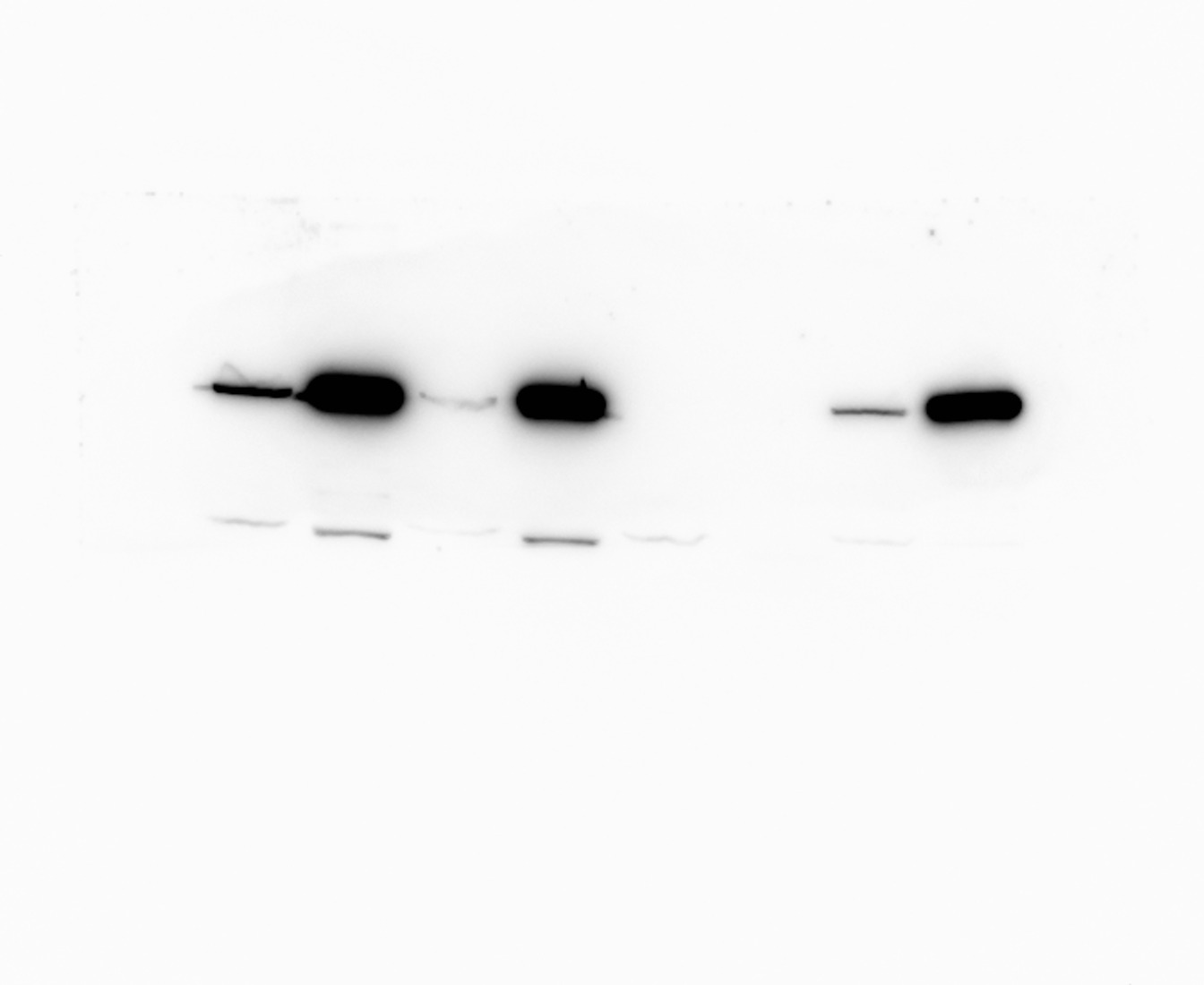

Supplement: Supplementary file 4 — Source data [file 41467_2024_53777_MOESM4_ESM.zip › Source-Data/FigureS11/Raw-data-FigureS11A/Replicate_3_Flag_Bands.jpg]

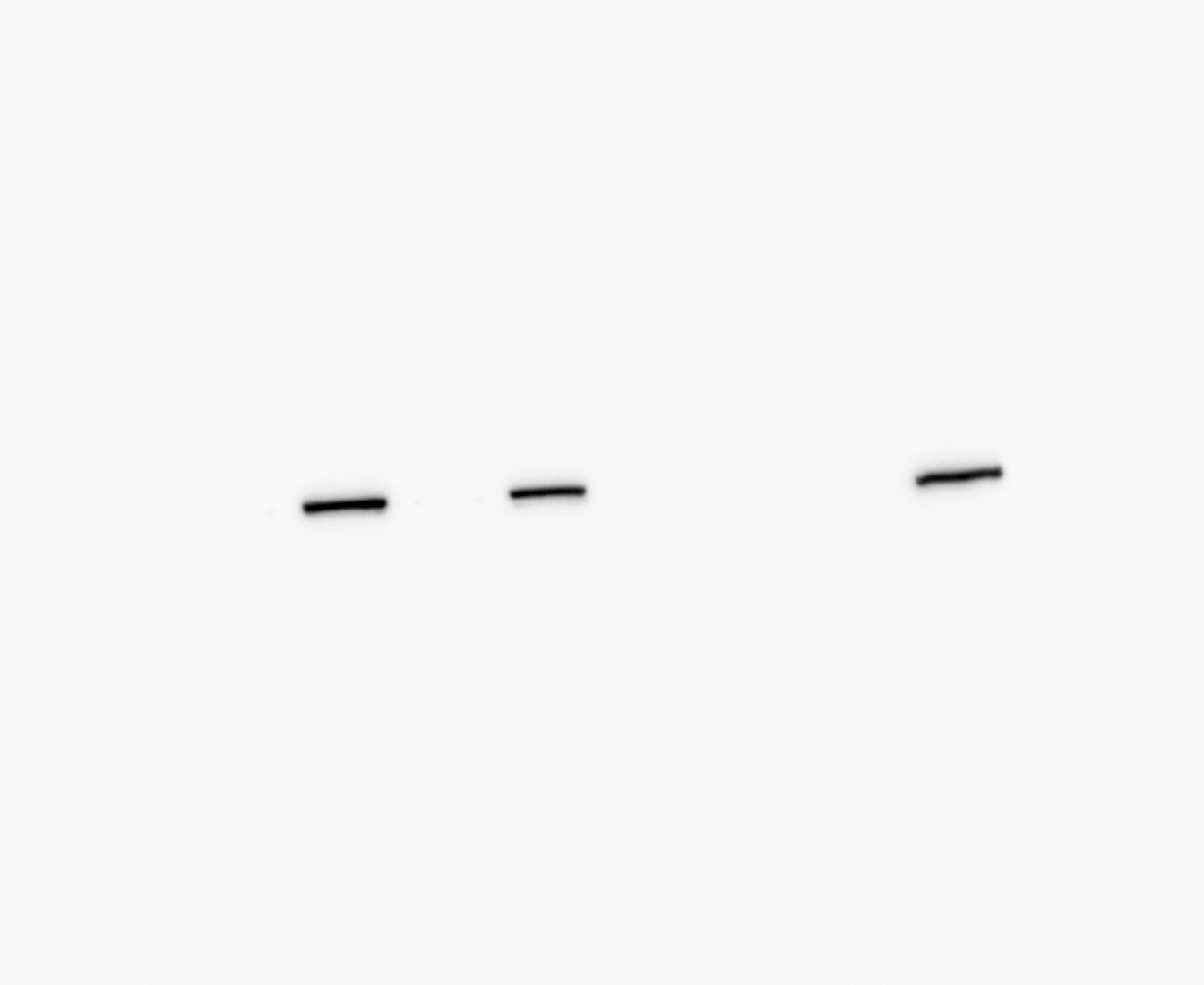

Supplement: Supplementary file 4 — Source data [file 41467_2024_53777_MOESM4_ESM.zip › Source-Data/FigureS11/Raw-data-FigureS11A/Replicate_2_Flag_Bands.jpg]

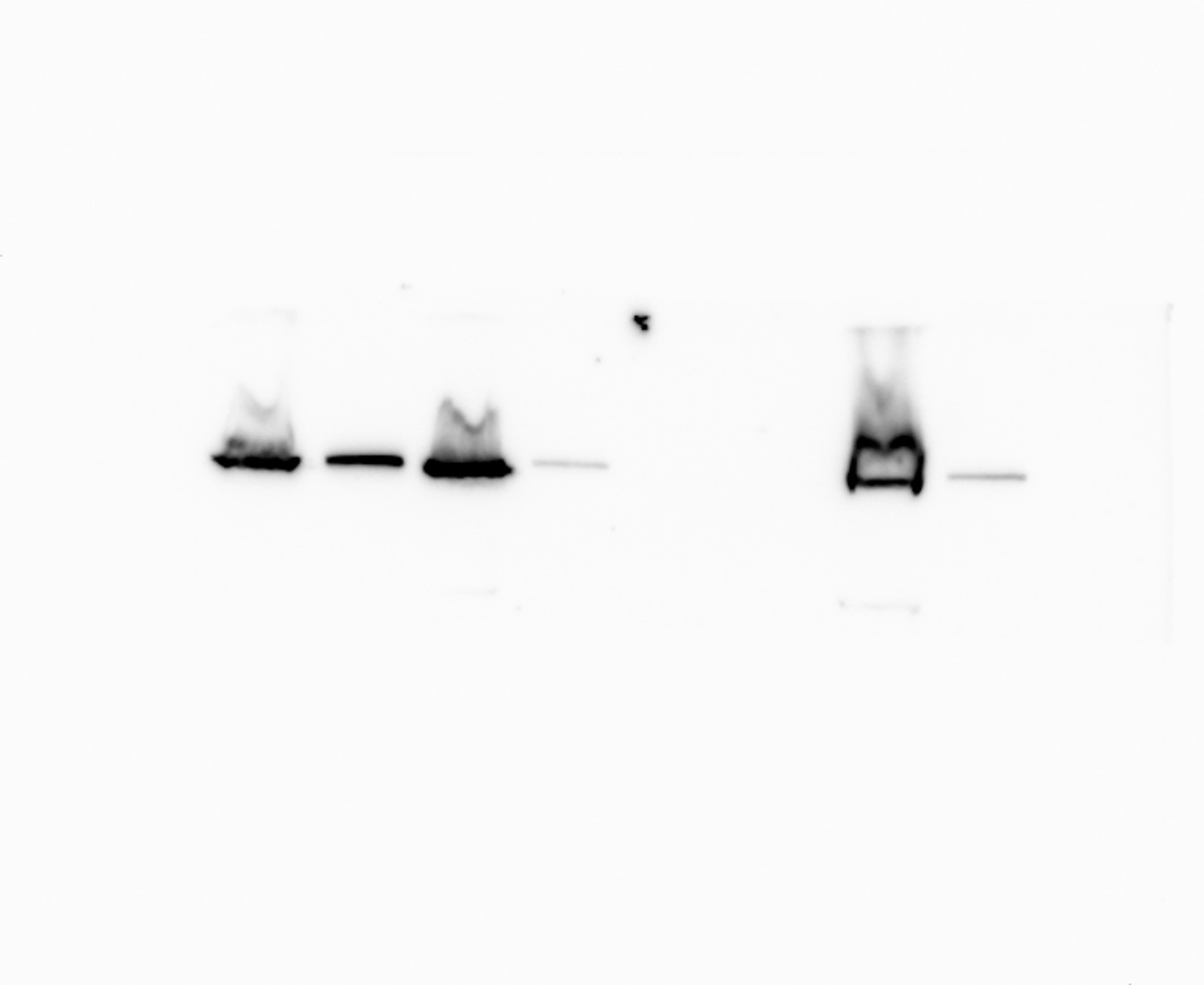

Supplement: Supplementary file 4 — Source data [file 41467_2024_53777_MOESM4_ESM.zip › Source-Data/FigureS11/Raw-data-FigureS11A/Replicate_2_HA_Bands.jpg]

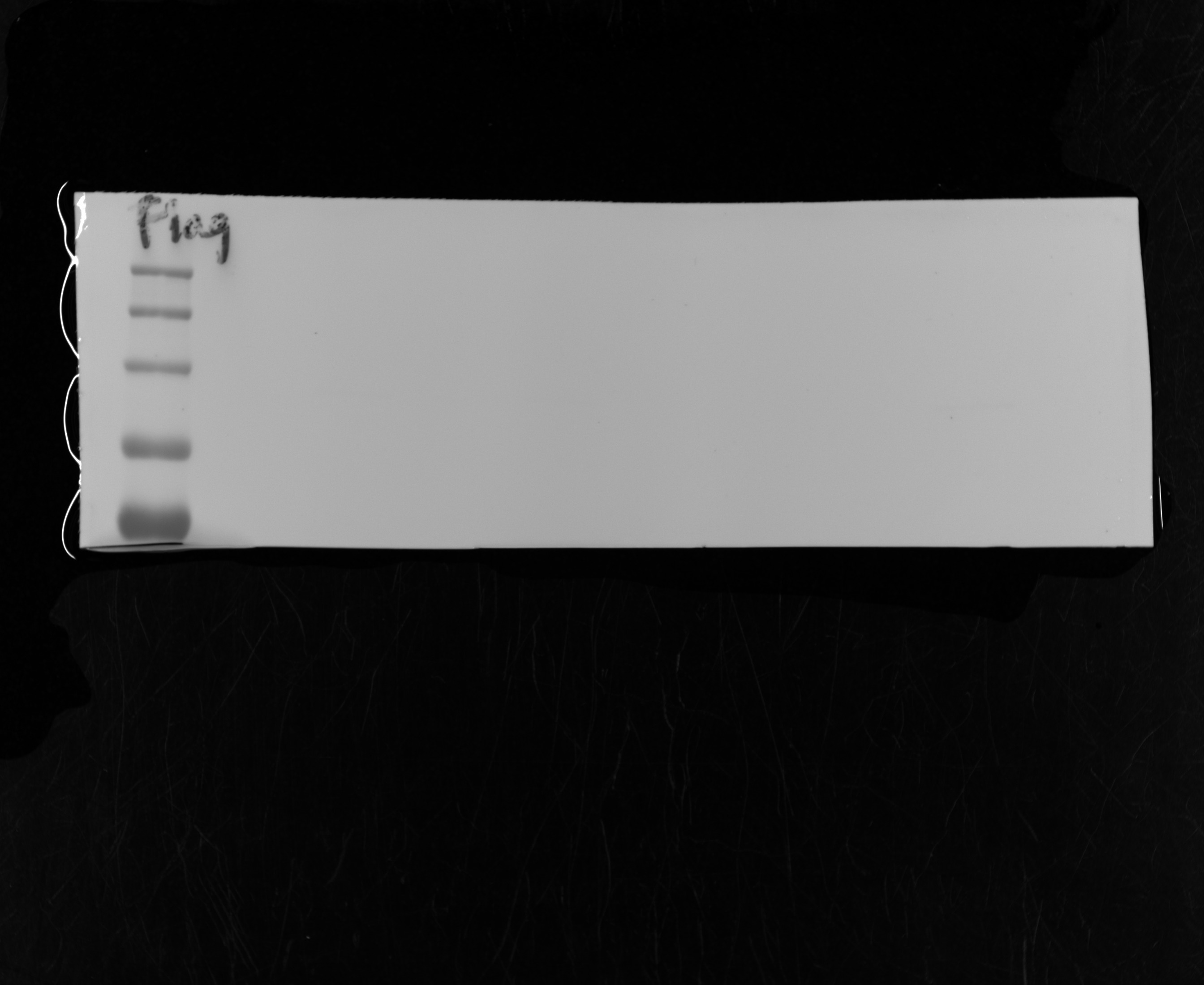

Supplement: Supplementary file 4 — Source data [file 41467_2024_53777_MOESM4_ESM.zip › Source-Data/FigureS11/Raw-data-FigureS11A/Replicate_3_Flag_Marker.jpg]

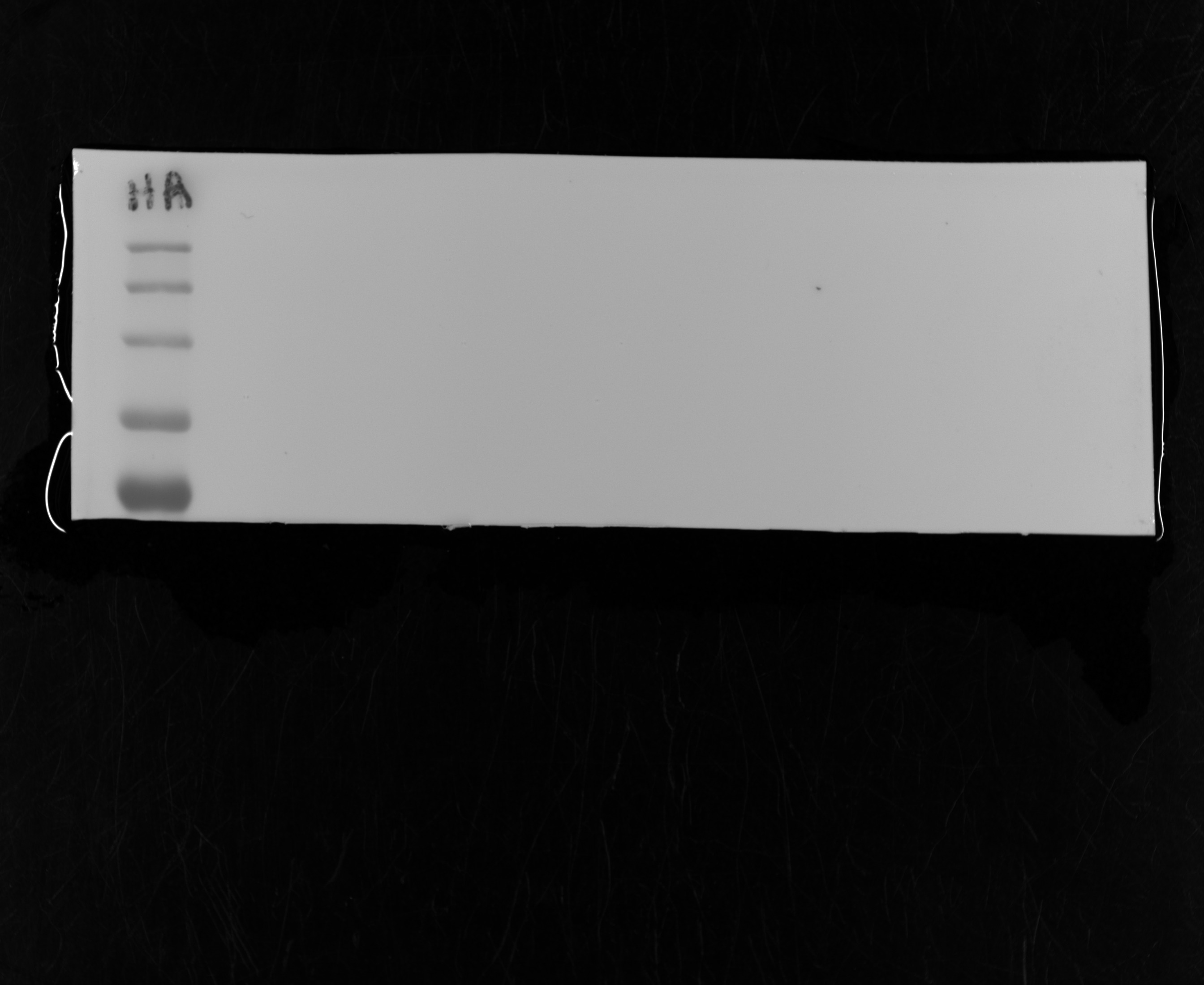

Supplement: Supplementary file 4 — Source data [file 41467_2024_53777_MOESM4_ESM.zip › Source-Data/FigureS11/Raw-data-FigureS11A/Replicate_3_HA_Marker.jpg]

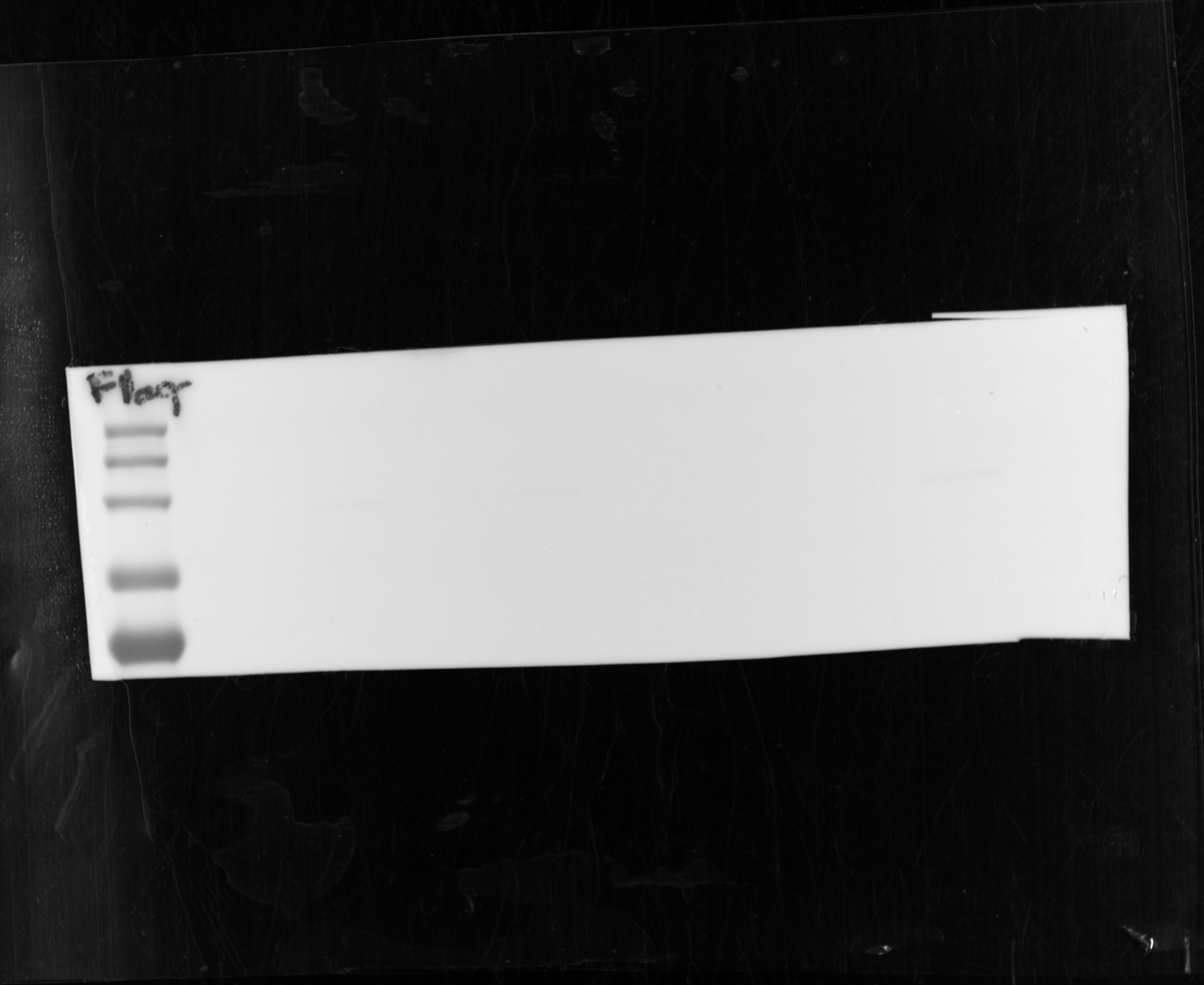

Supplement: Supplementary file 4 — Source data [file 41467_2024_53777_MOESM4_ESM.zip › Source-Data/FigureS11/Raw-data-FigureS11A/Replicate_2_Flag_Marker.jpg]
